# Supplementary material for: A High-Content Microscopy Screening Identifies New Genes Involved in Cell Width Control in Bacillus subtilis
Source: mSystems. 2021 Nov 30;6(6):e01017-21. doi: 10.1128/mSystems.01017-21 (PMC8631317; doi:10.1128/mSystems.01017-21)
Supplement: TABLE S1 [file msystems.01017-21-st001.pdf]

Sup. Table 1. Genes reported to affect cell width in *B. subtilis*

gene	fonction	link with CW	essentiality	effect[1]	references [2]	this study[3]
CW homeostasis						
mreB	rod complex regulation		no	+	Jones *et al.,* 01	na
mbl	rod complex regulation		no	+	Abhayawardhane *et al.,* 95, Jones *et al.,* 01	na
mreBH	rod complex regulation		no	-	Carballido-López *et al.* , 06; Sassine *et al.* , 20	no
mreC	rod complex transpeptidase regulator		no	R	Lee *et al.,* 03; Leaver *et al.,* 05	na
mreD	rod complex, unknown function		yes	R	Leaver *et al.,* 05	na
pbpA+pbpH	rod complex transpeptidases (bPBPs)		yes[4]	R	Wei *et al.,* 03	na
rodA	rod complex transglycosylase		yes	R	Henriques *et al.,* 98	na
rodZ	rod complex, regulator		no[5]	+	Muchova *et al.,* 13; Van Beilen *et al.* , 16	yes
murB	PG precursor synthetic pathway		yes	+	Peters *et al.,* 16	na
ponA	transglycosylase/transpeptidase (aPBP)		no	-	Popham *et al.,* 96; Claessen *et al.,* 08	yes
lytE	PG-hydrolase		no	?[6]	Dominguez-Cuevas *et al.,* 13; Carballido-López *et al.* , 06; Sassine *et al.* 20	no
cwlO	PG-hydrolase		no	+	Dominguez-Cuevas *et al.,* 13; Meisner *et al.,* 13	yes
ftsE	ABC transporter, activator of CwlO		no	+	Dominguez-Cuevas *et al.,* 13; Meisner *et al.,* 13	yes
ftsX	ABC transporter, activator of CwlO		no	+	Dominguez-Cuevas *et al.,* 13; Meisner *et al.,* 13	yes
tagT+TagV	Teichoic acid synthesis		no	+	Kawai *et al* ., 11	na
tagO	TA synthesis (first step)		no[8]	R	D'Elia *et al.,* 06	na
tagA	TA synthesis (first commited step)		no[8]	R	D'Elia *et al.,* 09	na
ltaS (yflE)	lipoteichoic acid synthase		no	-[9]	Sassine *et al.* , 20	na
yqgS	lipoteichoic acid synthase		no	-[9]	Sassine *et al.* , 20	na
yfnI (ltaSA)	lipoteichoic acid synthase		no	-[9]	Sassine *et al.* , 20	na
Other processes		link with CW				
glmR (yvcK)	regulation of C flux	stimulates PG precursor synthetic pathway	yes[7]	+[7]	Foulquier *et al.,* 11	na
rny (ymdA)	Rnase Y	affect *rodA* , *mreBCD* and *mreBH* expression	no	-	Figaro *et al.,* 13	no
rnJA	Rnase J1	affect *rodA* , *mreBCD* and *mreBH* expression	no	+	Figaro *et al.,* 13	na
cpgA	ribosome assembly & detoxification of erythronate-4P	detoxification prevents downstream depletion of CW precursors	no	+[9]	Cladiere *et al.* , 06	na
ezrA+gpsB	FtsZ inhibitor and late divisome protein	*ponA* localization	no	-	Claessen *et al.,* 08	na

1: + or - impact on cell width upon gene deletion or depletion; R stands for round cell, a consequence of the absence of elongation
2: references for measurement of width
3: mutants selected in the present screen; na (not available) indicates that the mutant could not be be find in this screen du to its absence from the BKK library, its essentiality or because the phenotype is synthetic
4: synthetically lethal
5: essential in Muchova *et al.,* 13 but inactivated in *B . subtilis* in Van Beilen *et al.,* 16, Koo *et al.* , 17, Kobayashi *et al.,* 03, and this study
6: thinner in Dominguez *et al.,* 13, Carballido *et al.,* 06, and Sassine *et al.,* 20 but wild type width in Meisner *et al.* , 13
7: only during neoglucogenic condition
8: erroneously annotated as essential in the subtiwiki database (and therefore absent from the BKK library)
9: growth condition-dependent
