# Supplementary material for: A High-Content Microscopy Screening Identifies New Genes Involved in Cell Width Control in Bacillus subtilis
Source: mSystems. 2021 Nov 30;6(6):e01017-21. doi: 10.1128/mSystems.01017-21 (PMC8631317; doi:10.1128/mSystems.01017-21)
Supplement: TABLE S2 [file msystems.01017-21-st002.pdf]

Sup. Table 2. Settings used for the MicrobeJ plugin

Particle selection parameters	
Area [µm²]:	3.7-20
Length [µm]:	0-max
Width [µm]:	0.5-2
- Range [µm]:	0-max
- Variation:	0-0.15
Circularity:	0.3-0.7
Curvature:	0-max
Sinuosity:	0-max
Angularity [rad]:	0-0.25
Solidity:	0.85-max
Intensity:	0-max
Segmentation method	
Dark, Otsu, median axis	
Treatment	
Thresholding:	Use ROI (enabled)
Resampling:	Resolution: 0.5, Method: Bilinear
Other:	Include Holes (enabled)
Pre-Processing (enabled):	Subtract Background, rolling=12
Threshold Calculator (enabled):	Binary, Dark, Otsu, offset: 0, area: 1000-max
