# Supplementary material for: A High-Content Microscopy Screening Identifies New Genes Involved in Cell Width Control in Bacillus subtilis
Source: mSystems. 2021 Nov 30;6(6):e01017-21. doi: 10.1128/mSystems.01017-21 (PMC8631317; doi:10.1128/mSystems.01017-21)
Supplement: TABLE S3 [file msystems.01017-21-st003.pdf]

Sup. Table 3. Average width differences (%) across replicates[1]

	WT 1	WT 2	WT 3	WT 4	WT 5	WT 6
WT 1	-	+1.7 %	+0.8 %	+0.5 %	+0.2 %	+0.1 %
WT 2	-	-	-0.9 %	-1.2 %	-1.5 %	-1.6 %
WT 3	-	-	-	-0.3 %	-0.6 %	-0.7 %
WT 4	-	-	-	-	-0.4 %	-0.4 %
WT 5	-	-	-	-	-	-0.1 %
width (µm)	1.01	1.028	1.012	1.016	1.013	1.012
+/-	0.076	0.080	0.071	0.076	0.089	0.071

1: ordinary one-way ANOVA test between replicates conclude to no significant differences
