# Supplementary figures and images for: A High-Content Microscopy Screening Identifies New Genes Involved in Cell Width Control in Bacillus subtilis
Source: mSystems. 2021 Nov 30;6(6):e01017-21. doi: 10.1128/mSystems.01017-21 (PMC8631317; doi:10.1128/mSystems.01017-21)

Fig. Sup. 1.

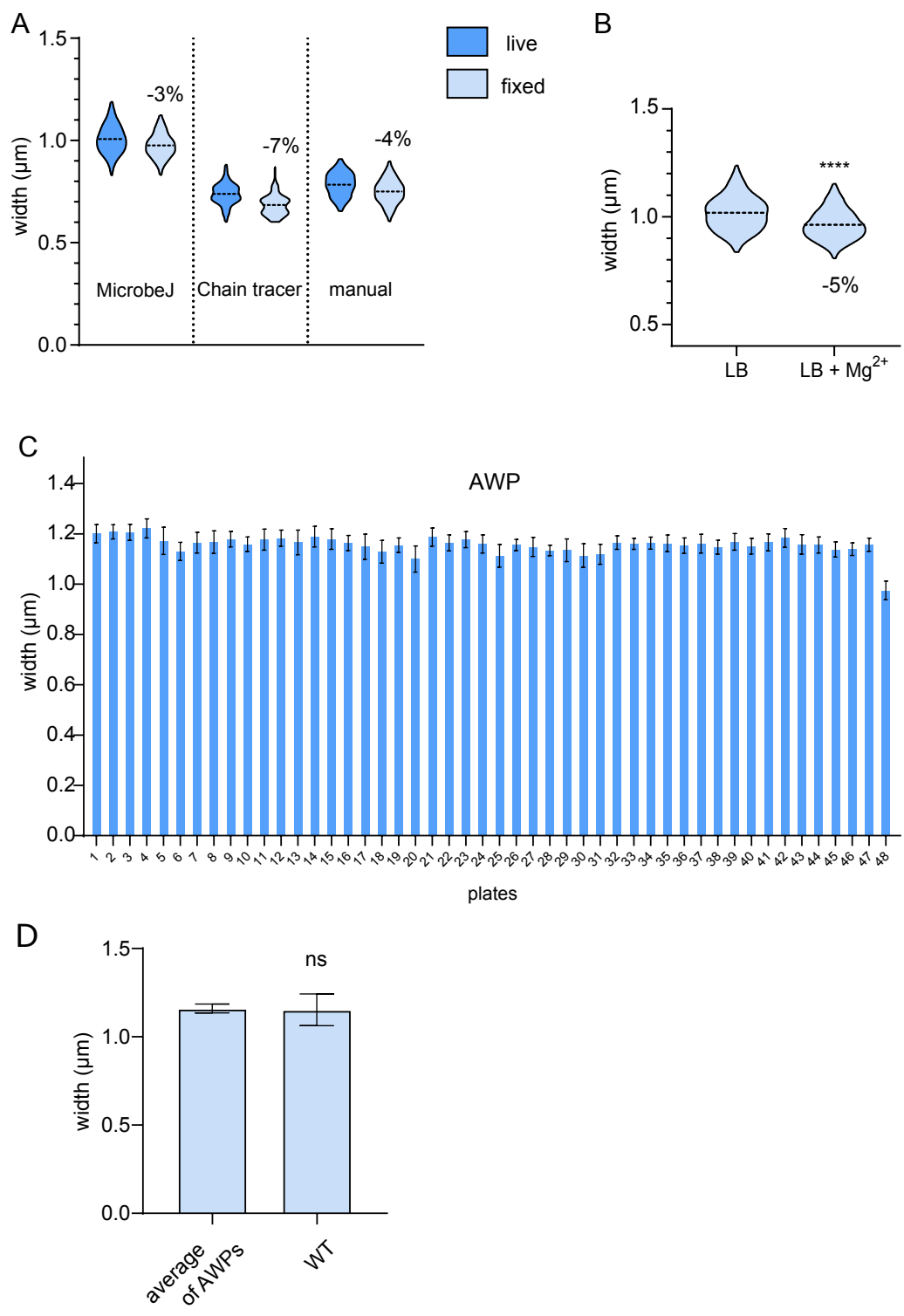

Supplement: FIG S1 [file msystems.01017-21-sf001.pdf]

Fig. Sup. 2.

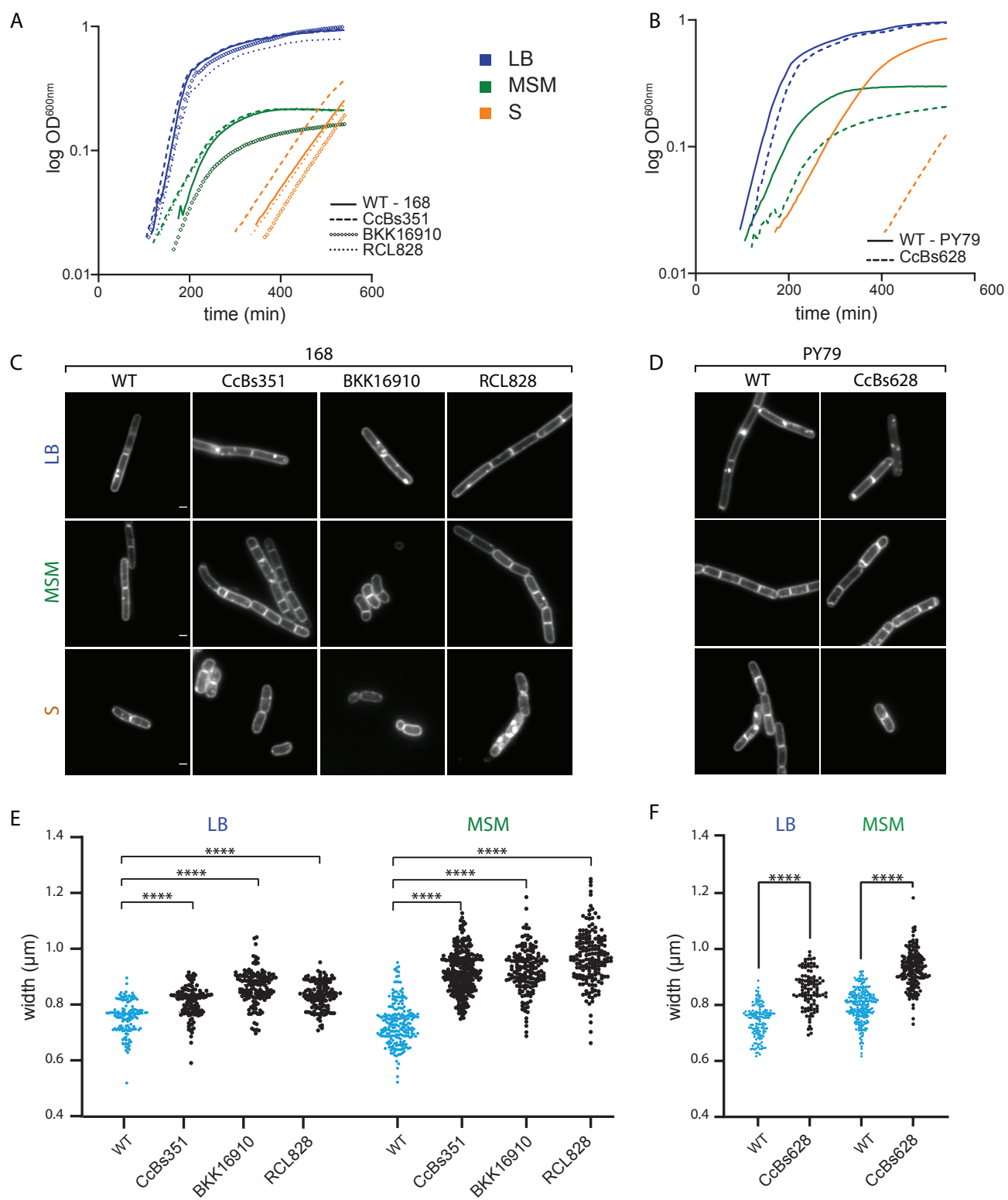

Supplement: FIG S2 [file msystems.01017-21-sf002.pdf]
