# Supplementary material for: A High-Content Microscopy Screening Identifies New Genes Involved in Cell Width Control in Bacillus subtilis
Source: mSystems. 2021 Nov 30;6(6):e01017-21. doi: 10.1128/mSystems.01017-21 (PMC8631317; doi:10.1128/mSystems.01017-21)
Supplement: TABLE S4 [file msystems.01017-21-st004.pdf]

Sup. Table 4. Cell width of mutants of the BKK collection

BKK name [1]	gene	screening delta[2] (%)	average width (µm)	+/-	nb	AWP
**BKK34800**	*cwlO*	23.357	1.418	0.136	301	1.149
**BKK12760**	*xkdW*	21.842	1.421	0.125	150	1.166
**BKK35260**	*ftsE*	19.51	1.383	0.153	109	1.158
**BKK22849**	*ypzH*	15.994	1.276	0.133	401	1.100
**BKK00100**	*dacA*	15.94	1.342	0.149	107	1.158
**BKK35250**	*ftsX*	15.586	1.329	0.183	286	1.149
**BKK20680**	*yoqC*	13.238	1.323	0.180	411	1.168
**BKK16825**	*ymfD*	12.668	1.309	0.172	82	1.162
**BKK27320**	*greA*	12.654	1.098	0.100	40	0.975
**BKK35450**	*comFC*	11.474	1.242	0.102	90	1.114
**BKK16910**	*rodZ*	11.399	1.294	0.108	287	1.162
**BKK31070**	*yuaC*	11.327	1.293	0.130	238	1.161
**BKK12770**	*xkdX*	10.489	1.289	0.121	135	1.166
**BKK00030**	*yaaA*	9.941	1.283	0.122	189	1.167
**BKK17000**	*kbl*	9.825	1.276	0.164	152	1.162
**BKK35440**	*yvyF*	9.578	1.221	0.111	161	1.114
**BKK23990**	*yqiW*	9.495	1.263	0.141	142	1.153
**BKK35220**	*minJ*	9.381	1.242	0.131	104	1.135
**BKK13030**	*ykhA*	9.169	1.273	0.183	109	1.166
**BKK02750**	*natA*	9.096	1.311	0.124	127	1.201
BKK19550	*yodC*	9.083	1.232	0.110	425	1.129
BKK18230	*yngG*	8.914	1.276	0.084	255	1.172
BKK36280	*ywqA*	8.891	1.213	0.146	347	1.114
BKK15450	*lspA*	8.741	1.210	0.108	210	1.113
BKK28800	*araA*	8.593	1.209	0.100	31	1.113
BKK15730	*fmt*	8.544	1.058	0.086	72	0.975
BKK08690	*ygaD*	8.54	1.312	0.134	34	1.208
BKK05344	*ydzS*	8.47	1.266	0.099	161	1.167
BKK35520	*yvhJ*	8.434	1.246	0.130	196	1.149
BKK16660	*truB*	8.397	1.261	0.121	75	1.164
BKK34110	*rsbP*	8.384	1.230	0.109	124	1.135
BKK36130	*ywrA*	8.371	1.057	0.089	33	0.975
BKK19440	*norM*	8.352	1.224	0.130	613	1.129
BKK23520	*fur*	8.293	1.249	0.140	93	1.153
BKK36269	*ywzD*	8.241	1.252	0.112	101	1.157
BKK29280	*ytnM*	8.103	1.203	0.118	155	1.113
BKK12619	*ykzL*	7.983	1.282	0.140	81	1.187
BKK14750	*ylaE*	7.862	1.272	0.183	72	1.179
BKK09840	*hemZ*	7.816	1.269	0.127	140	1.177
BKK27290	*yrzA*	7.775	1.268	0.128	45	1.177
BKK34760	*yvcK*	7.743	1.223	0.105	271	1.135
BKK12850	*ykaA*	7.694	1.252	0.130	271	1.163
BKK19370	*odhA*	7.657	1.216	0.124	315	1.129
BKK31580	*maeN*	7.642	1.236	0.099	103	1.148
BKK13280	*ykoJ*	7.628	1.255	0.166	168	1.166
BKK31570	*yufQ*	7.616	1.315	0.129	119	1.222
BKK38229	*slrA*	7.495	1.203	0.112	144	1.119
BKK23250	*ribH*	7.444	1.275	0.102	339	1.187
BKK18320	*ppsC*	7.406	1.258	0.113	91	1.172
BKK04410	*ydbB*	7.404	1.251	0.109	372	1.165
BKK01950	*skfE*	7.358	1.222	0.097	31	1.138
BKK00330	*yabA*	7.321	1.265	0.127	430	1.179
BKK18150	*xynC*	7.264	1.257	0.112	231	1.172
BKK18130	*eglS*	7.223	1.256	0.116	231	1.172
BKK33710	*opuBC*	7.139	1.232	0.108	486	1.149
BKK39020	*yxjA*	7.131	1.293	0.161	103	1.206
BKK14570	*ykyA*	7.126	1.263	0.180	32	1.179
BKK29730	*ytxD*	7.116	1.192	0.093	30	1.113
BKK30050	*ytgP*	7.092	1.309	0.213	112	1.222
BKK22770	*mtrB*	7.077	1.235	0.143	232	1.153
BKK24930	*yqzD*	7.065	1.246	0.104	415	1.164
BKK01700	*murQ*	7.045	1.218	0.146	34	1.138
BKK12672	*xkzB*	7.034	1.273	0.176	40	1.189
BKK26720	*azlB*	6.983	1.259	0.109	392	1.177
BKK22620	*hisC*	6.963	1.177	0.121	304	1.100
BKK05330	*aseR*	6.954	1.248	0.068	122	1.167
BKK19510	*yojB*	6.876	1.242	0.141	243	1.162
BKK03040	*amyE*	6.864	1.208	0.137	60	1.130
BKK03651	*bsdD*	6.852	1.245	0.102	150	1.165
BKK34130	*ganA*	6.791	1.212	0.115	30	1.135
BKK02610	*ycbR*	6.782	1.207	0.128	56	1.130
BKK04770	*ydcH*	6.741	1.243	0.138	298	1.165
BKK22530	*ypjA*	6.735	1.216	0.103	76	1.139
BKK28450	*sdhC*	6.734	1.238	0.097	32	1.160
BKK31370	*yugJ*	6.723	1.225	0.095	618	1.148
BKK34560	*malL*	6.715	1.211	0.142	116	1.135
BKK39360	*hutU*	6.694	1.244	0.152	105	1.166
BKK04060	*ycsG*	6.653	1.242	0.082	146	1.165
BKK35150	*yvzB*	6.63	1.210	0.141	62	1.135

BKK name [1]	gene	screening delta[2] (%)	average width (µm)	+/-	nb	AWP
BKK19390	*yojN*	6.61	1.204	0.116	324	1.129
BKK36380	*rapD*	6.61	1.188	0.116	769	1.114
BKK10100	*yhgC*	6.591	1.255	0.115	206	1.177
BKK37680	*ywfH*	6.58	1.192	0.099	116	1.119
BKK25250	*ccpN*	6.574	1.240	0.106	98	1.164
BKK19530	*yodA*	6.542	1.203	0.155	502	1.129
BKK36390	*flhP*	6.537	1.187	0.123	160	1.114
BKK36860	*atpE*	6.487	1.222	0.128	253	1.147
BKK25840	*phrE*	6.454	1.038	0.095	43	0.975
BKK23160	*rluB*	6.451	1.264	0.105	212	1.187
BKK13840	*stoA*	6.443	1.242	0.123	39	1.166
BKK31150	*uppP*	6.429	1.236	0.110	286	1.161
BKK11590	*yjbL*	6.402	1.265	0.169	516	1.189
BKK26590	*blt*	6.37	1.300	0.088	72	1.222
BKK14090	*ykuI*	6.306	1.240	0.133	387	1.166
BKK29400	*ytlI*	6.306	1.235	0.108	257	1.161
BKK10210	*yhfF*	6.304	1.251	0.109	116	1.177
BKK05450	*ydfK*	6.302	1.241	0.116	146	1.167
BKK21750	*scuA*	6.3	1.226	0.142	215	1.153
BKK15570	*cysH*	6.219	1.219	0.099	35	1.148
BKK15270	*sbp*	6.206	1.252	0.143	140	1.179
BKK32080	*yuiB*	6.197	1.219	0.079	152	1.148
BKK00610	*yabQ*	6.179	1.244	0.108	94	1.172
BKK13140	*ohrA*	6.162	1.234	0.146	212	1.163
BKK37350	*sboA*	6.153	1.188	0.110	116	1.119
BKK40290	*yycN*	6.108	1.221	0.122	299	1.151
BKK24220	*spo0A*	6.107	1.257	0.101	161	1.184
BKK15590	*sat*	6.105	1.239	0.130	241	1.168
BKK09490	*yhdJ*	6.083	1.249	0.121	86	1.177
BKK23030	*ypbB*	6.074	1.221	0.124	294	1.151
BKK09620	*yhdW*	6.056	1.248	0.117	148	1.177
BKK22990	*ypbF*	6.034	1.167	0.099	570	1.100
BKK05650	*ydgH*	6.024	1.238	0.126	149	1.167
BKK18400	*yoeD*	6.008	1.242	0.087	327	1.172
BKK18250	*yngI*	5.923	1.241	0.117	178	1.172
BKK38480	*ywaC*	5.912	1.185	0.127	30	1.119
BKK05390	*ydfF*	5.893	1.236	0.090	391	1.167
BKK04460	*dctR*	5.887	1.233	0.128	392	1.165
BKK24720	*comGB*	5.883	1.232	0.124	251	1.164
BKK23280	*ribD*	5.871	1.257	0.113	218	1.187
BKK15780	*cpgA*	5.86	1.032	0.156	41	0.975
BKK32100	*yumB*	5.847	1.215	0.095	63	1.148
BKK17890	*tkt*	5.816	1.229	0.120	550	1.162
BKK34730	*yvcN*	5.799	1.201	0.124	592	1.135
BKK25700	*yqeF*	5.798	1.220	0.125	367	1.153
BKK40980	*yyaB*	5.775	1.253	0.116	97	1.184
BKK22660	*trpC*	5.766	1.217	0.127	348	1.151
BKK20650	*yoqF*	5.748	1.221	0.130	194	1.155
BKK03820	*yclP*	5.726	1.203	0.122	89	1.138
BKK15620	*sirB*	5.71	1.213	0.092	100	1.148
BKK06220	*ydjJ*	5.698	1.246	0.111	285	1.179
BKK23460	*spoIIAB*	5.696	1.275	0.161	93	1.206
BKK13810	*ykvS*	5.695	1.229	0.136	221	1.163
BKK23120	*resD*	5.674	1.163	0.099	350	1.100
BKK23510	*ripX*	5.671	1.254	0.095	148	1.187
BKK05490	*ydfO*	5.658	1.233	0.115	151	1.167
BKK29870	*ytzB*	5.644	1.030	0.079	100	0.975
BKK35820	*gerBC*	5.632	1.214	0.107	408	1.149
BKK31500	*yuxK*	5.625	1.212	0.147	56	1.148
BKK40810	*yyaM*	5.597	1.233	0.148	144	1.167
BKK00050	*yaaB*	5.574	1.245	0.126	178	1.179
BKK25650	*yqeI*	5.559	1.228	0.099	346	1.164
BKK08820	*katA*	5.529	1.242	0.124	162	1.177
BKK11300	*med*	5.512	1.249	0.132	67	1.183
BKK30740	*mntD*	5.498	1.214	0.123	218	1.151
BKK35670	*gtaB*	5.489	1.175	0.131	218	1.114
BKK26060	*yqbM*	5.483	1.241	0.104	292	1.177
BKK34910	*hisD*	5.475	1.197	0.092	377	1.135
BKK35570	*tuaE*	5.475	1.212	0.132	504	1.149
BKK37460	*rapF*	5.469	1.180	0.131	258	1.119
BKK10300	*aprE*	5.445	1.248	0.141	117	1.183
BKK03490	*srfAB*	5.444	1.028	0.085	80	0.975
BKK03290	*nasE*	5.431	1.191	0.145	37	1.130
BKK22700	*aroB*	5.422	1.249	0.114	132	1.184
BKK02670	*lmrB*	5.417	1.200	0.124	52	1.138
BKK21030	*yonP*	5.411	1.216	0.117	398	1.153
BKK27480	*yrrC*	5.403	1.222	0.105	146	1.160
BKK04130	*yczJ*	5.388	1.228	0.130	143	1.165
BKK22630	*trpA*	5.379	1.159	0.107	118	1.100

BKK name [1]	gene	screening delta[2] (%)	average width (µm)	+/-	nb	AWP
BKK39260	*bglH*	5.364	1.227	0.120	79	1.165
BKK34870	*hisF*	5.362	1.196	0.100	246	1.135
BKK40579	*yyzL*	5.346	1.229	0.128	85	1.166
BKK29410	*ytkL*	5.34	1.223	0.128	410	1.161
BKK40260	*yycQ*	5.338	1.213	0.115	203	1.151
BKK09340	*yhdA*	5.327	1.240	0.101	102	1.177
BKK33630	*secG*	5.322	1.211	0.090	354	1.149
BKK04070	*ycsI*	5.301	1.227	0.100	92	1.165
BKK02960	*yceJ*	5.29	1.265	0.148	47	1.201
BKK11580	*yjbK*	5.282	1.252	0.132	921	1.189
BKK10950	*yitD*	5.271	1.246	0.123	70	1.183
BKK09990	*scoC*	5.267	1.239	0.105	67	1.177
BKK19540	*yodB*	5.241	1.188	0.117	565	1.129
BKK04450	*dctS*	5.231	1.264	0.094	65	1.201
BKK25380	*yqfA*	5.211	1.224	0.105	148	1.164
BKK32820	*fadE*	5.205	1.227	0.114	96	1.166
BKK14810	*ylaK*	5.201	1.240	0.071	86	1.179
BKK12671	*xkdN*	5.181	1.251	0.174	77	1.189
BKK33130	*liaI*	5.172	1.193	0.118	62	1.134
BKK03410	*bglC*	5.146	1.188	0.133	38	1.130
BKK37110	*ywjH*	5.144	1.176	0.141	115	1.119
BKK19669	*yoyF*	5.14	1.187	0.133	315	1.129
BKK02880	*yceB*	5.132	1.188	0.130	67	1.130
BKK04930	*yddD*	5.113	1.217	0.092	96	1.158
BKK27490	*yrrB*	5.109	1.219	0.110	148	1.160
BKK39810	*csbC*	5.096	1.208	0.114	422	1.149
BKK01830	*ndhF*	5.086	1.219	0.107	274	1.160
BKK05720	*ydhE*	5.085	1.227	0.081	366	1.167
BKK14510	*ktrC*	5.078	1.239	0.097	359	1.179
BKK19520	*yojA*	5.07	1.187	0.116	686	1.129
BKK00170	*yaaI*	5.06	1.218	0.124	606	1.160
BKK18160	*xynD*	5.052	1.231	0.115	249	1.172
BKK05350	*ydfB*	5.036	1.226	0.075	179	1.167
BKK16990	*tdh*	5.036	1.222	0.105	110	1.164
BKK38440	*ywaF*	5.015	1.175	0.125	32	1.119
BKK29890	*ytnP*	5.002	1.169	0.121	39	1.113
BKK11630	*prpE*	4.999	1.248	0.140	803	1.189
BKK23402	*spoVAEB*	4.99	1.267	0.187	36	1.206
BKK14500	*ykqA*	4.976	1.238	0.116	301	1.179
BKK16860	*ymfH*	4.967	1.221	0.127	82	1.164
BKK22740	*hepT*	4.961	1.155	0.101	110	1.100
BKK37170	*acdA*	4.938	1.224	0.116	89	1.166
BKK01800	*alkA*	4.929	1.217	0.104	324	1.160
BKK09370	*lytF*	4.921	1.268	0.136	47	1.208
BKK17440	*ynbB*	4.909	1.221	0.114	160	1.164
BKK06630	*yerH*	4.908	1.260	0.073	89	1.201
BKK05130	*ydeB*	4.893	1.224	0.112	366	1.167
BKK28320	*ysnD*	4.891	1.216	0.103	447	1.160
BKK30170	*ytcP*	4.889	1.282	0.140	33	1.222
BKK26700	*azlD*	4.888	1.282	0.078	201	1.222
BKK34880	*hisA*	4.887	1.191	0.086	270	1.135
BKK24450	*efp*	4.884	1.245	0.108	402	1.187
BKK29510	*ytfI*	4.882	1.167	0.110	102	1.113
BKK31740	*yuxH*	4.882	1.204	0.092	121	1.148
BKK35020	*nagBA*	4.879	1.190	0.123	126	1.135
BKK35679	*yvzH*	4.867	1.205	0.122	239	1.149
BKK11700	*thiF*	4.86	1.247	0.143	929	1.189
BKK11990	*yjdB*	4.847	1.247	0.180	70	1.189
BKK28750	*araN*	4.846	1.281	0.078	69	1.222
BKK03810	*yclO*	4.844	1.260	0.073	113	1.201
BKK22040	*ypbQ*	4.843	1.154	0.147	35	1.100
BKK21040	*yonO*	4.833	1.209	0.108	308	1.153
BKK18430	*yogA*	4.827	1.228	0.086	84	1.172
BKK09900	*natB*	4.82	1.234	0.091	98	1.177
BKK08920	*yhbB*	4.802	1.218	0.120	207	1.163
BKK09965	*yhaJ*	4.797	1.233	0.131	93	1.177
BKK35390	*yviE*	4.79	1.189	0.123	430	1.135
BKK24510	*yqhO*	4.783	1.206	0.112	311	1.151
BKK40000	*yxnA*	4.779	1.202	0.121	256	1.147
BKK00320	*yaaT*	4.777	1.235	0.114	214	1.179
BKK29500	*ytfJ*	4.769	1.166	0.100	181	1.113
BKK16120	*topA*	4.768	1.241	0.126	110	1.184
BKK34400	*padC*	4.765	1.189	0.096	68	1.135
BKK24680	*comGF*	4.754	1.208	0.140	178	1.153
BKK12090	*cotT*	4.749	1.245	0.203	112	1.189
BKK24500	*yqhP*	4.746	1.206	0.106	404	1.151
BKK33670	*rghRB*	4.743	1.188	0.139	33	1.134
BKK03050	*ldh*	4.742	1.184	0.127	46	1.130
BKK21720	*ypmT*	4.739	1.206	0.132	237	1.151

BKK name [1]	gene	screening delta[2] (%)	average width (µm)	+/-	nb	AWP
BKK10690	*gerPD*	4.734	1.239	0.107	93	1.183
BKK27580	*yrvJ*	4.725	1.280	0.064	102	1.222
BKK27710	*tgt*	4.718	1.214	0.101	352	1.160
BKK29920	*ytmP*	4.71	1.166	0.128	41	1.113
BKK20690	*yoqB*	4.7	1.209	0.112	317	1.155
BKK26580	*bltR*	4.698	1.279	0.110	56	1.222
BKK06076	*ydzW*	4.693	1.222	0.146	449	1.167
BKK05408	*ydzP*	4.67	1.193	0.138	32	1.139
BKK12380	*uxaB*	4.666	1.244	0.170	666	1.189
BKK06200	*ydjH*	4.662	1.234	0.129	191	1.179
BKK05640	*ydgG*	4.653	1.222	0.092	289	1.167
BKK27930	*spo0B*	4.648	1.207	0.145	126	1.153
BKK12600	*xkdF*	4.635	1.244	0.125	181	1.189
BKK27400	*yrzL*	4.631	1.213	0.118	464	1.160
BKK14830	*glsB*	4.626	1.234	0.082	201	1.179
BKK34180	*yvfI*	4.619	1.188	0.101	230	1.135
BKK09020	*yhcB*	4.618	1.231	0.109	97	1.177
BKK33180	*yvrC*	4.613	1.278	0.109	77	1.222
BKK27600	*relA*	4.606	1.213	0.098	421	1.160
BKK38530	*dltD*	4.605	1.202	0.166	142	1.149
BKK01845	*ybcC*	4.599	1.213	0.104	268	1.160
BKK37320	*narK*	4.597	1.200	0.108	349	1.147
BKK28650	*ysgA*	4.59	1.164	0.117	121	1.113
BKK18019	*ynzL*	4.588	1.217	0.119	105	1.164
BKK07480	*yfmG*	4.582	1.212	0.114	333	1.159
BKK24950	*pstBB*	4.578	1.262	0.148	150	1.206
BKK34380	*slrR*	4.578	1.187	0.087	234	1.135
BKK04480	*ydbI*	4.563	1.256	0.091	43	1.201
BKK28620	*rnhC*	4.561	1.164	0.099	170	1.113
BKK30390	*bceS*	4.561	1.278	0.090	74	1.222
BKK13450	*sigI*	4.549	1.219	0.136	43	1.166
BKK02760	*natB*	4.547	1.256	0.177	90	1.201
BKK37960	*ywdH*	4.547	1.200	0.114	436	1.147
BKK20310	*yorO*	4.516	1.214	0.109	191	1.162
BKK23260	*ribAB*	4.514	1.241	0.121	254	1.187
BKK17430	*ynbA*	4.513	1.216	0.109	71	1.164
BKK35410	*flgK*	4.507	1.165	0.107	1066	1.114
BKK02980	*opuAA*	4.495	1.255	0.132	69	1.201
BKK06180	*pspA*	4.493	1.232	0.117	192	1.179
BKK40560	*yybP*	4.489	1.219	0.123	132	1.166
BKK03840	*ycnB*	4.477	1.217	0.105	221	1.165
BKK33660	*rghRA*	4.473	1.019	0.076	55	0.975
BKK09230	*yhcV*	4.472	1.230	0.096	153	1.177
BKK16000	*ylqC*	4.461	1.169	0.120	241	1.119
BKK22380	*ypmB*	4.45	1.149	0.105	218	1.100
BKK14270	*moeB*	4.448	1.232	0.109	344	1.179
BKK31900	*yukD*	4.448	1.199	0.111	148	1.148
BKK23680	*yqjZ*	4.446	1.202	0.112	340	1.151
BKK20700	*yoqA*	4.444	1.206	0.116	398	1.155
BKK31700	*comX*	4.443	1.218	0.102	223	1.166
BKK04830	*sacV*	4.439	1.217	0.091	355	1.165
BKK02230	*purT*	4.437	1.180	0.123	85	1.130
BKK11670	*thiO*	4.437	1.242	0.153	883	1.189
BKK04810	*immA*	4.435	1.217	0.123	156	1.165
BKK40210	*yydC*	4.434	1.218	0.193	47	1.166
BKK12080	*ctaO*	4.427	1.242	0.183	156	1.189
BKK17010	*ymcB*	4.416	1.215	0.120	146	1.164
BKK06340	*yeaD*	4.414	1.198	0.134	301	1.147
BKK23270	*ribE*	4.409	1.239	0.103	363	1.187
BKK27370	*yrrL*	4.406	1.211	0.105	221	1.160
BKK05290	*ydeP*	4.398	1.219	0.084	214	1.167
BKK03380	*yckB*	4.396	1.254	0.130	282	1.201
BKK25460	*dnaJ*	4.382	1.215	0.126	211	1.164
BKK01900	*ybcM*	4.373	1.162	0.112	35	1.113
BKK06380	*yebC*	4.371	1.230	0.144	366	1.179
BKK26259	*yqzO*	4.358	1.228	0.100	128	1.177
BKK35210	*yvkA*	4.356	1.200	0.117	376	1.149
BKK37000	*prmC*	4.352	1.163	0.130	78	1.114
BKK38018	*ywzG*	4.346	1.217	0.127	71	1.166
BKK28700	*ysfE*	4.331	1.161	0.078	32	1.113
BKK38850	*yxkC*	4.329	1.215	0.125	58	1.165
BKK10390	*yhfW*	4.319	1.234	0.091	176	1.183
BKK03788	*yczM*	4.309	1.207	0.138	43	1.158
BKK14590	*pdhB*	4.304	1.230	0.148	301	1.179
BKK02780	*ycdA*	4.3	1.253	0.212	33	1.201
BKK08860	*ssuD*	4.292	1.227	0.109	234	1.177
BKK16740	*spoVFB*	4.285	1.213	0.122	89	1.164
BKK20670	*yoqD*	4.284	1.204	0.124	286	1.155
BKK34150	*ganP*	4.283	1.184	0.115	62	1.135

Sup. Table 4: Cell width of mutants of the BKK collection (continued)

BKK name [1]	gene	screening delta[2] (%)	average width (µm)	+/-	nb	AWP
BKK00340	*yabB*	4.272	1.230	0.152	79	1.179
BKK26150	*yqbD*	4.272	1.017	0.091	96	0.975
BKK29040	*ytbD*	4.268	1.274	0.076	114	1.222
BKK39070	*bglS*	4.253	1.214	0.137	96	1.165
BKK01990	*ybdG*	4.252	1.178	0.132	69	1.130
BKK35940	*rbsA*	4.231	1.198	0.130	336	1.149
BKK29050	*ytbE*	4.217	1.160	0.111	70	1.113
BKK14370	*yknZ*	4.216	1.229	0.120	77	1.179
BKK27785	*yrzF*	4.209	1.208	0.099	405	1.160
BKK25900	*cwlA*	4.196	1.273	0.073	192	1.222
BKK20250	*mtbP*	4.195	1.211	0.127	199	1.162
BKK13680	*motB*	4.193	1.217	0.122	296	1.168
BKK31230	*tlpB*	4.193	1.204	0.131	144	1.156
BKK17330	*miaA*	4.187	1.212	0.128	109	1.164
BKK34729	*yvzJ*	4.186	1.198	0.122	289	1.149
BKK22000	*sspL*	4.185	1.203	0.115	238	1.155
BKK09130	*tcyP*	4.177	1.226	0.117	77	1.177
BKK10060	*ecsC*	4.17	1.217	0.120	300	1.168
BKK38400	*epr*	4.17	1.195	0.112	399	1.147
BKK15370	*ylmD*	4.169	1.187	0.122	53	1.139
BKK25760	*spoIVCB*	4.165	1.273	0.102	232	1.222
BKK00810	*dusB*	4.162	1.208	0.126	361	1.160
BKK31350	*pgi*	4.162	1.196	0.120	103	1.148
BKK12000	*manR*	4.158	1.238	0.171	444	1.189
BKK11180	*yitZ*	4.147	1.232	0.136	118	1.183
BKK24620	*tasA*	4.144	1.015	0.117	64	0.975
BKK05340	*ydfA*	4.14	1.015	0.085	47	0.975
BKK19400	*sodC*	4.136	1.176	0.131	489	1.129
BKK05140	*ydzE*	4.135	1.208	0.127	633	1.160
BKK20210	*yorY*	4.135	1.176	0.134	88	1.129
BKK36010	*alsS*	4.13	1.160	0.114	47	1.114
BKK37430	*albG*	4.125	1.197	0.114	403	1.149
BKK12400	*yjnA*	4.11	1.238	0.150	551	1.189
BKK19570	*yodE*	4.104	1.176	0.098	440	1.129
BKK39600	*yxeC*	4.103	1.195	0.107	270	1.147
BKK40529	*yyzH*	4.103	1.214	0.118	105	1.166
BKK30000	*ythQ*	4.095	1.272	0.174	141	1.222
BKK25110	*yqfU*	4.094	1.211	0.103	647	1.164
BKK07770	*yfkS*	4.086	1.216	0.129	357	1.168
BKK12190	*yjhB*	4.08	1.238	0.095	267	1.189
BKK07860	*yfkL*	4.073	1.258	0.161	100	1.208
BKK31100	*ktrB*	4.058	1.208	0.137	223	1.161
BKK02350	*nagP*	4.056	1.176	0.128	139	1.130
BKK08880	*rpsNB*	4.054	1.225	0.099	151	1.177
BKK33210	*yvrG*	4.052	1.214	0.105	121	1.166
BKK29070	*ytaF*	4.045	1.203	0.099	79	1.156
BKK13819	*ykzS*	4.043	1.214	0.108	259	1.166
BKK24970	*pstA*	4.033	1.255	0.156	51	1.206
BKK23210	*scpB*	4.03	1.232	0.111	79	1.184
BKK02010	*ybdK*	4.027	1.184	0.128	137	1.138
BKK28310	*ilvB*	4.024	1.206	0.119	295	1.160
BKK34210	*yvfG*	4.022	1.181	0.108	85	1.135
BKK27910	*yszB*	4.011	1.206	0.121	200	1.160
BKK12980	*ykfB*	4.008	1.257	0.101	35	1.208
BKK23320	*ypzC*	4.001	1.235	0.111	107	1.187
BKK26890	*csn*	3.998	1.224	0.108	318	1.177
BKK24890	*yqgN*	3.985	1.210	0.099	280	1.164
BKK40970	*parA*	3.973	1.232	0.104	130	1.184
BKK32120	*yuzG*	3.965	1.193	0.110	30	1.148
BKK10009	*yhzF*	3.952	1.223	0.105	148	1.177
BKK07680	*yflH*	3.95	1.205	0.119	206	1.159
BKK33830	*opuCA*	3.95	1.195	0.110	332	1.149
BKK24560	*gcvPA*	3.945	1.234	0.099	271	1.187
BKK30430	*ytrD*	3.931	1.207	0.113	280	1.161
BKK30340	*ytvA*	3.929	1.207	0.113	277	1.161
BKK07750	*yflA*	3.917	1.214	0.129	253	1.168
BKK09889	*yhzD*	3.905	1.223	0.126	134	1.177
BKK37560	*thrZ*	3.9	1.162	0.101	154	1.119
BKK08800	*ygaK*	3.887	1.204	0.127	394	1.159
BKK14260	*mobA*	3.883	1.225	0.121	282	1.179
BKK11540	*pepF*	3.879	1.229	0.113	89	1.183
BKK25289	*yqzL*	3.878	1.209	0.094	332	1.164
BKK07280	*yfnG*	3.874	1.225	0.149	108	1.179
BKK10620	*addB*	3.874	1.229	0.102	114	1.183
BKK30490	*ytqB*	3.873	1.201	0.104	140	1.156
BKK08210	*malQ*	3.87	1.255	0.111	31	1.208
BKK20460	*yoqZ*	3.87	1.173	0.157	67	1.129
BKK18610	*yoaH*	3.867	1.217	0.113	138	1.172
BKK33430	*cysI*	3.864	1.013	0.078	41	0.975

BKK name [1]	gene	screening delta[2] (%)	average width (µm)	+/-	nb	AWP
BKK06160	*gutP*	3.856	1.224	0.143	116	1.179
BKK03220	*ycgO*	3.843	1.173	0.104	114	1.130
BKK35460	*comFB*	3.826	1.157	0.090	103	1.114
BKK18239	*yngHB*	3.824	1.216	0.086	342	1.172
BKK15390	*sepF*	3.82	1.213	0.119	153	1.168
BKK08050	*yfjL*	3.818	1.254	0.139	74	1.208
BKK26260	*yqaM*	3.811	1.222	0.111	255	1.177
BKK07250	*yetO*	3.808	1.202	0.092	78	1.158
BKK08740	*ygzB*	3.808	1.203	0.110	507	1.159
BKK19670	*yodN*	3.804	1.172	0.145	185	1.129
BKK04359	*ydzK*	3.799	1.209	0.091	552	1.165
BKK05630	*dinB*	3.797	1.212	0.075	188	1.167
BKK04720	*rsbW*	3.788	1.209	0.106	403	1.165
BKK10310	*yhfO*	3.786	1.228	0.112	141	1.183
BKK18220	*yngF*	3.784	1.216	0.099	132	1.172
BKK34840	*yvcB*	3.784	1.178	0.113	132	1.135
BKK37180	*fadF*	3.778	1.161	0.130	401	1.119
BKK14550	*ykrA*	3.775	1.224	0.154	69	1.179
BKK38310	*ywbI*	3.775	1.161	0.102	43	1.119
BKK06130	*ydjC*	3.769	1.223	0.141	198	1.179
BKK12230	*yjjA*	3.761	1.234	0.090	127	1.189
BKK29590	*iscS*	3.761	1.155	0.127	70	1.113
BKK02990	*opuAB*	3.756	1.247	0.101	134	1.201
BKK06370	*pbuG*	3.753	1.223	0.136	384	1.179
BKK28950	*thrS*	3.749	1.155	0.097	32	1.113
BKK03640	*ubiD*	3.748	1.209	0.112	170	1.165
BKK13610	*mtnB*	3.744	1.210	0.145	74	1.166
BKK32660	*yurT*	3.744	1.177	0.088	90	1.134
BKK24150	*mmgC*	3.741	1.231	0.103	395	1.187
BKK09760	*yheE*	3.733	1.221	0.118	133	1.177
BKK12260	*yjlA*	3.732	1.233	0.065	115	1.189
BKK15970	*ylxM*	3.729	1.160	0.132	73	1.119
BKK11560	*yjbI*	3.718	1.211	0.114	200	1.168
BKK13750	*queF*	3.704	1.210	0.133	258	1.166
BKK07760	*yfkT*	3.698	1.202	0.123	371	1.159
BKK18620	*yoaI*	3.698	1.215	0.081	250	1.172
BKK26920	*yraJ*	3.693	1.220	0.110	292	1.177
BKK15260	*ylxX*	3.681	1.223	0.110	622	1.179
BKK29650	*ytrP*	3.678	1.154	0.082	59	1.113
BKK40350	*rocR*	3.667	1.193	0.121	257	1.151
BKK38090	*vpr*	3.665	1.160	0.125	302	1.119
BKK23010	*ypbD*	3.663	1.141	0.114	591	1.100
BKK23560	*mleN*	3.653	1.230	0.104	191	1.187
BKK29820	*ytpR*	3.65	1.154	0.108	88	1.113
BKK11650	*tenA*	3.647	1.211	0.129	253	1.168
BKK09720	*yheH*	3.638	1.252	0.163	44	1.208
BKK33000	*htrB*	3.629	1.203	0.107	475	1.161
BKK06410	*yebG*	3.618	1.189	0.111	296	1.147
BKK13509	*ykzP*	3.604	1.208	0.138	159	1.166
BKK21520	*yolC*	3.601	1.196	0.115	408	1.155
BKK40790	*yyaO*	3.598	1.189	0.121	369	1.147
BKK09480	*yhdI*	3.596	1.219	0.123	129	1.177
BKK09010	*yhcA*	3.591	1.204	0.112	245	1.163
BKK16210	*fliF*	3.591	1.204	0.111	224	1.163
BKK05160	*ydeD*	3.58	1.209	0.116	65	1.167
BKK39520	*yxeK*	3.577	1.206	0.115	67	1.165
BKK07900	*yfkH*	3.576	1.200	0.124	95	1.159
BKK18270	*ynzE*	3.559	1.213	0.099	191	1.172
BKK26980	*yraE*	3.559	1.219	0.106	284	1.177
BKK12060	*yjdI*	3.557	1.231	0.133	340	1.189
BKK10640	*sbcD*	3.556	1.225	0.100	164	1.183
BKK20910	*yopF*	3.553	1.196	0.106	336	1.155
BKK11640	*yjbQ*	3.547	1.231	0.137	824	1.189
BKK07720	*yflD*	3.544	1.200	0.116	327	1.159
BKK36270	*ywqB*	3.538	1.208	0.103	95	1.166
BKK38770	*cimH*	3.533	1.178	0.118	53	1.138
BKK09520	*sigM*	3.531	1.219	0.150	65	1.177
BKK27390	*yrrK*	3.521	1.200	0.099	448	1.160
BKK40650	*yybG*	3.521	1.226	0.116	154	1.184
BKK40990	*noc*	3.518	1.226	0.100	70	1.184
BKK07630	*nosA*	3.514	1.200	0.111	433	1.159
BKK06320	*yeaB*	3.513	1.200	0.114	382	1.160
BKK29780	*ytxG*	3.513	1.179	0.086	114	1.139
BKK15380	*ylmE*	3.511	1.221	0.117	47	1.179
BKK31321	*mstX*	3.508	1.197	0.108	92	1.156
BKK01740	*rsiW*	3.506	1.244	0.073	33	1.201
BKK08660	*sspE*	3.503	1.199	0.113	358	1.159
BKK38410	*sacX*	3.502	1.197	0.106	134	1.157
BKK34420	*yveF*	3.499	1.175	0.106	150	1.135

BKK name [1]	gene	screening delta[2] (%)	average width (µm)	+/-	nb	AWP
BKK09740	*yheF*	3.49	1.218	0.113	105	1.177
BKK16640	*ylxP*	3.487	1.204	0.112	110	1.164
BKK37990	*ywdE*	3.475	1.187	0.110	391	1.147
BKK39480	*yxeO*	3.474	1.264	0.091	72	1.222
BKK01650	*ybbC*	3.467	1.243	0.105	34	1.201
BKK12450	*yjpA*	3.466	1.179	0.128	64	1.139
BKK39320	*yxiB*	3.458	1.205	0.113	144	1.165
BKK21530	*yolB*	3.455	1.195	0.128	462	1.155
BKK03280	*nasF*	3.452	1.169	0.134	64	1.130
BKK08425	*mprF*	3.446	1.208	0.116	230	1.168
BKK21598	*yoyK*	3.446	1.191	0.117	243	1.151
BKK23190	*dacB*	3.446	1.248	0.168	48	1.206
BKK38600	*licR*	3.442	1.205	0.112	189	1.165
BKK18340	*ppsA*	3.441	1.212	0.085	43	1.172
BKK24830	*yqgT*	3.441	1.248	0.165	154	1.206
BKK21160	*yonA*	3.435	1.195	0.114	404	1.155
BKK07350	*yfmT*	3.429	1.208	0.124	257	1.168
BKK38380	*ywbB*	3.425	1.196	0.120	64	1.157
BKK15410	*ylmH*	3.412	1.219	0.153	72	1.179
BKK00490	*spoVG*	3.402	1.177	0.124	51	1.138
BKK15820	*rpmB*	3.397	1.174	0.102	30	1.135
BKK04990	*yddJ*	3.396	1.199	0.104	98	1.160
BKK06269	*ydzJ*	3.393	1.219	0.125	242	1.179
BKK31180	*yulE*	3.387	1.195	0.129	89	1.156
BKK04220	*ydaG*	3.382	1.204	0.115	274	1.165
BKK06270	*ydjO*	3.377	1.219	0.115	143	1.179
BKK06730	*yefA*	3.372	1.219	0.132	263	1.179
BKK22760	*hepS*	3.371	1.192	0.109	161	1.153
BKK25300	*cdd*	3.365	1.203	0.102	299	1.164
BKK25470	*dnaK*	3.365	1.203	0.103	324	1.164
BKK14009	*ykzT*	3.364	1.229	0.158	168	1.189
BKK20800	*yopQ*	3.364	1.201	0.110	349	1.162
BKK40140	*yydJ*	3.363	1.206	0.140	78	1.166
BKK26040	*yqbN*	3.362	1.216	0.094	148	1.177
BKK00700	*coaX*	3.358	1.178	0.097	106	1.139
BKK01620	*feuB*	3.358	1.216	0.107	517	1.177
BKK22830	*gpsA*	3.352	1.137	0.115	44	1.100
BKK20440	*yorB*	3.336	1.167	0.145	88	1.129
BKK24330	*yqhY*	3.333	1.189	0.123	307	1.151
BKK30750	*mntC*	3.328	1.263	0.088	47	1.222
BKK13060	*ykjA*	3.323	1.205	0.098	44	1.166
BKK09730	*yheG*	3.32	1.216	0.123	146	1.177
BKK26050	*yqdB*	3.32	1.192	0.124	242	1.153
BKK11450	*oppC*	3.318	1.207	0.122	237	1.168
BKK40280	*yycO*	3.318	1.189	0.114	311	1.151
BKK26180	*yqbA*	3.317	1.216	0.117	139	1.177
BKK09200	*yhcS*	3.316	1.176	0.111	59	1.138
BKK08970	*prkA*	3.298	1.216	0.121	163	1.177
BKK28460	*yslB*	3.296	1.196	0.097	87	1.158
BKK16200	*fliE*	3.292	1.201	0.109	347	1.163
BKK05730	*ydhF*	3.291	1.198	0.105	346	1.160
BKK29480	*ytxK*	3.29	1.150	0.132	82	1.113
BKK37830	*spsJ*	3.286	1.156	0.146	53	1.119
BKK05740	*phoB*	3.278	1.205	0.083	202	1.167
BKK14450	*ampS*	3.274	1.218	0.103	66	1.179
BKK39030	*yxiT*	3.271	1.203	0.133	117	1.165
BKK13799	*ykzR*	3.256	1.204	0.120	177	1.166
BKK21610	*yokF*	3.25	1.188	0.114	341	1.151
BKK08270	*yfiH*	3.247	1.196	0.117	410	1.159
BKK21970	*ypeP*	3.241	1.192	0.108	486	1.155
BKK17080	*pksA*	3.24	1.201	0.119	62	1.164
BKK34430	*racX*	3.238	1.187	0.114	381	1.149
BKK34320	*epsF*	3.232	1.187	0.109	371	1.149
BKK29270	*ytoI*	3.23	1.149	0.099	186	1.113
BKK34570	*malK*	3.223	1.172	0.123	31	1.135
BKK22680	*trpE*	3.222	1.188	0.114	242	1.151
BKK22700	*aroB*	3.222	1.136	0.094	42	1.100
BKK35470	*comFA*	3.222	1.199	0.118	398	1.161
BKK07880	*yfkJ*	3.209	1.206	0.116	272	1.168
BKK10630	*addA*	3.208	1.221	0.105	125	1.183
BKK14140	*ccpC*	3.208	1.204	0.117	884	1.166
BKK30700	*rpmEB*	3.206	1.195	0.104	119	1.158
BKK13970	*ykwD*	3.198	1.200	0.128	215	1.163
BKK32490	*pucC*	3.195	1.185	0.122	40	1.148
BKK30950	*glgA*	3.188	1.193	0.098	122	1.156
BKK37290	*arfM*	3.17	1.154	0.106	59	1.119
BKK04290	*ydaL*	3.166	1.202	0.099	156	1.165
BKK26760	*yrdC*	3.156	1.214	0.110	172	1.177
BKK11410	*yjbA*	3.155	1.221	0.134	94	1.183

BKK name [1]	gene	screening delta[2] (%)	average width (µm)	+/-	nb	AWP
BKK12680	*xkdO*	3.154	1.227	0.162	83	1.189
BKK35320	*fliT*	3.153	1.171	0.124	449	1.135
BKK36160	*ywqM*	3.153	1.149	0.126	92	1.114
BKK15350	*thiQ*	3.152	1.216	0.152	168	1.179
BKK09330	*yhcZ*	3.15	1.205	0.118	286	1.168
BKK11010	*yitJ*	3.134	1.220	0.094	130	1.183
BKK39129	*yxzI*	3.13	1.201	0.116	114	1.165
BKK14072	*ykzU*	3.128	1.203	0.153	401	1.166
BKK21070	*yonJ*	3.124	1.191	0.126	185	1.155
BKK40460	*yyzB*	3.116	1.203	0.105	128	1.166
BKK06210	*ydjI*	3.115	1.196	0.108	899	1.160
BKK32669	*yuzN*	3.114	1.170	0.103	160	1.134
BKK24790	*yqgX*	3.11	1.200	0.100	262	1.164
BKK09560	*cueR*	3.108	1.246	0.119	96	1.208
BKK07830	*yfkO*	3.104	1.195	0.120	398	1.159
BKK27620	*recJ*	3.104	1.196	0.104	151	1.160
BKK35130	*yvlA*	3.101	1.170	0.104	377	1.135
BKK39830	*yxcA*	3.097	1.193	0.102	115	1.158
BKK26630	*yrdQ*	3.093	1.189	0.119	303	1.153
BKK03570	*sfp*	3.091	1.193	0.107	80	1.158
BKK07840	*yfkN*	3.09	1.195	0.112	454	1.159
BKK25660	*aroD*	3.09	1.200	0.101	462	1.164
BKK38360	*ywbD*	3.085	1.153	0.135	37	1.119
BKK05610	*vmlR*	3.078	1.195	0.125	425	1.160
BKK40960	*parB*	3.071	1.221	0.101	118	1.184
BKK28560	*lcfA*	3.07	1.192	0.110	102	1.156
BKK29000	*nrdR*	3.063	1.193	0.094	114	1.158
BKK08870	*ygaN*	3.062	1.213	0.108	128	1.177
BKK10150	*yhgD*	3.062	1.213	0.103	138	1.177
BKK01550	*gerD*	3.061	1.173	0.112	35	1.138
BKK06600	*pcrB*	3.061	1.215	0.113	261	1.179
BKK27420	*yrrI*	3.06	1.189	0.112	290	1.153
BKK14890	*ctaC*	3.059	1.215	0.109	327	1.179
BKK14770	*bipA*	3.055	1.215	0.097	259	1.179
BKK09980	*yhaI*	3.054	1.245	0.158	82	1.208
BKK08250	*yfiF*	3.052	1.194	0.129	108	1.159
BKK28540	*fadB*	3.049	1.005	0.094	159	0.975
BKK11110	*yitS*	3.048	1.219	0.099	76	1.183
BKK27809	*yrzT*	3.048	1.195	0.103	164	1.160
BKK39010	*yxjB*	3.048	1.200	0.116	101	1.165
BKK27130	*rsiV*	3.047	1.259	0.067	80	1.222
BKK16180	*flgB*	3.045	1.204	0.101	348	1.168
BKK37620	*rsfA*	3.044	1.153	0.107	78	1.119
BKK31020	*yuaF*	3.043	1.197	0.120	371	1.161
BKK00750	*pabA*	3.041	1.134	0.116	53	1.100
BKK02920	*yceF*	3.039	1.238	0.152	67	1.201
BKK38590	*licB*	3.037	1.200	0.114	83	1.165
BKK08290	*yfiJ*	3.034	1.245	0.172	39	1.208
BKK13550	*mtnA*	3.034	1.202	0.126	162	1.166
BKK19450	*rsbRC*	3.027	1.163	0.118	459	1.129
BKK31710	*comQ*	3.02	1.183	0.123	52	1.148
BKK22870	*fni*	3.018	1.133	0.100	251	1.100
BKK07590	*citT*	3.013	1.203	0.103	261	1.168
BKK17320	*ymaF*	3.007	1.199	0.096	117	1.164
BKK33680	*yvaP*	2.985	1.168	0.109	71	1.134
BKK23640	*yqkD*	2.984	1.222	0.109	241	1.187
BKK00950	*mrnC*	2.982	1.222	0.099	150	1.187
BKK27670	*spoVB*	2.966	1.258	0.069	80	1.222
BKK24070	*buk*	2.959	1.222	0.113	356	1.187
BKK09210	*yhcT*	2.958	1.212	0.098	147	1.177
BKK20010	*yosT*	2.957	1.163	0.123	351	1.129
BKK26170	*yqbB*	2.955	1.211	0.100	159	1.177
BKK05360	*ydfC*	2.947	1.202	0.089	163	1.167
BKK23930	*yqjC*	2.942	1.222	0.097	343	1.187
BKK03630	*ubiX*	2.94	1.199	0.109	77	1.165
BKK10510	*yhjH*	2.939	1.218	0.093	161	1.183
BKK25520	*yqxA*	2.937	1.185	0.103	262	1.151
BKK09630	*yhdX*	2.931	1.211	0.120	85	1.177
BKK35350	*yvyC*	2.928	1.168	0.106	233	1.135
BKK06520	*purH*	2.922	1.213	0.122	487	1.179
BKK31959	*ybdZ*	2.92	1.181	0.107	230	1.148
BKK14200	*ykuS*	2.919	1.214	0.147	767	1.179
BKK38550	*ilvK*	2.917	1.191	0.135	85	1.157
BKK20930	*yopD*	2.914	1.189	0.122	528	1.155
BKK37920	*ywdL*	2.914	1.191	0.112	67	1.157
BKK06590	*yerD*	2.912	1.213	0.137	180	1.179
BKK23080	*aroC*	2.909	1.132	0.097	247	1.100
BKK05500	*ydfP*	2.908	1.201	0.092	309	1.167
BKK23440	*spoVAA*	2.906	1.242	0.157	38	1.206

Sup. Table 4: Cell width of mutants of the BKK collection (continued)

BKK name[1]	gene	screening delta[2] (%)	average width (µm)	+/-	nb	AWP
BKK09360	*yhdC*	2.9	1.211	0.092	94	1.177
BKK29080	*mutM*	2.9	1.195	0.120	488	1.161
BKK08730	*perR*	2.899	1.191	0.103	116	1.158
BKK35080	*yvmB*	2.895	1.168	0.125	76	1.135
BKK03950	*ycnJ*	2.894	1.199	0.085	135	1.165
BKK39550	*yxeH*	2.888	1.198	0.101	161	1.165
BKK09460	*ctrA*	2.887	1.196	0.111	268	1.163
BKK18390	*yoeC*	2.885	1.195	0.110	289	1.162
BKK18680	*yoaO*	2.883	1.205	0.101	61	1.172
BKK00080	*yaaC*	2.872	1.193	0.126	254	1.160
BKK06560	*yerA*	2.872	1.213	0.104	402	1.179
BKK38640	*yxlH*	2.869	1.191	0.108	155	1.158
BKK24660	*yqzE*	2.868	1.197	0.106	173	1.164
BKK06033	*ydzT*	2.867	1.201	0.088	410	1.167
BKK06880	*yesF*	2.866	1.213	0.142	279	1.179
BKK32190	*yuzB*	2.864	1.181	0.138	110	1.148
BKK13950	*mcpC*	2.857	1.200	0.144	76	1.166
BKK23620	*yqkF*	2.844	1.186	0.125	296	1.153
BKK32600	*frlO*	2.843	1.257	0.129	176	1.222
BKK27210	*yrhF*	2.841	1.210	0.105	358	1.177
BKK06720	*dagK*	2.84	1.218	0.125	80	1.184
BKK05790	*ydhK*	2.839	1.200	0.092	148	1.167
BKK13920	*splA*	2.839	1.199	0.124	135	1.166
BKK28330	*ysnE*	2.838	1.186	0.124	521	1.153
BKK40820	*yyaL*	2.836	1.218	0.102	168	1.184
BKK27720	*queA*	2.831	1.192	0.120	201	1.160
BKK14160	*ykuO*	2.83	1.213	0.135	1011	1.179
BKK14670	*suhB*	2.829	1.213	0.125	249	1.179
BKK33040	*fumC*	2.827	1.166	0.087	48	1.134
BKK10530	*ntdC*	2.821	1.201	0.107	252	1.168
BKK07000	*yesR*	2.82	1.212	0.125	227	1.179
BKK01460	*ybaE*	2.819	1.170	0.116	62	1.138
BKK18830	*pps*	2.819	1.170	0.090	121	1.138
BKK39500	*yxeM*	2.818	1.256	0.074	65	1.222
BKK21790	*yplQ*	2.809	1.186	0.110	344	1.153
BKK06490	*purF*	2.803	1.212	0.121	358	1.179
BKK18260	*yngJ*	2.798	1.204	0.116	59	1.172
BKK33950	*cggR*	2.798	1.166	0.099	107	1.134
BKK19240	*yocK*	2.796	1.194	0.121	410	1.162
BKK20760	*yopU*	2.796	1.187	0.105	503	1.155
BKK12300	*uxaC*	2.795	1.222	0.142	347	1.189
BKK20720	*yopY*	2.791	1.201	0.124	192	1.168
BKK26730	*yrdF*	2.79	1.210	0.094	77	1.177
BKK38930	*yxjJ*	2.787	1.197	0.130	46	1.165
BKK18290	*yngL*	2.786	1.194	0.118	340	1.162
BKK26010	*yqbQ*	2.786	1.196	0.115	72	1.164
BKK38300	*thiM*	2.781	1.199	0.110	115	1.166
BKK18420	*ftsR*	2.779	1.204	0.083	219	1.172
BKK25569	*yqzM*	2.779	1.196	0.102	333	1.164
BKK38720	*yxkO*	2.777	1.179	0.110	397	1.147
BKK34990	*lgt*	2.773	1.181	0.108	535	1.149
BKK01730	*sigW*	2.77	1.192	0.087	103	1.160
BKK23370	*ypuA*	2.77	1.185	0.124	247	1.153
BKK08770	*ygzA*	2.769	1.191	0.122	552	1.159
BKK25950	*yqcC*	2.766	1.002	0.100	88	0.975
BKK29150	*ytwI*	2.765	1.193	0.116	466	1.161
BKK22400	*dinG*	2.757	1.131	0.117	87	1.100
BKK40720	*yyaT*	2.754	1.217	0.096	113	1.184
BKK09165	*yhcO*	2.751	1.200	0.117	336	1.168
BKK40710	*yybA*	2.751	1.217	0.096	171	1.184
BKK04590	*ydbS*	2.75	1.197	0.091	424	1.165
BKK18669	*yozS*	2.75	1.194	0.120	196	1.162
BKK20530	*yoqS*	2.749	1.200	0.105	292	1.168
BKK15830	*yloU*	2.745	1.145	0.096	102	1.114
BKK16730	*spoVFA*	2.74	1.240	0.125	96	1.206
BKK24940	*yqzC*	2.732	1.195	0.114	222	1.164
BKK28200	*lonA*	2.732	1.183	0.120	278	1.151
BKK07930	*yfkD*	2.729	1.190	0.109	450	1.159
BKK12040	*yjdG*	2.727	1.221	0.157	285	1.189
BKK12130	*yjfC*	2.727	1.221	0.157	285	1.189
BKK03020	*ycgA*	2.726	1.234	0.075	101	1.201
BKK34100	*rsbQ*	2.726	1.166	0.153	96	1.135
BKK22540	*qcrC*	2.721	1.130	0.119	419	1.100
BKK15530	*pyrK*	2.72	1.187	0.103	87	1.156
BKK34010	*yvbW*	2.719	1.189	0.097	77	1.158
BKK16440	*cheW*	2.714	1.193	0.106	399	1.161
BKK19930	*yotC*	2.704	1.193	0.131	428	1.162
BKK13180	*metE*	2.696	1.170	0.130	39	1.139
BKK07800	*treP*	2.69	1.190	0.115	388	1.159

BKK name[1]	gene	screening delta[2] (%)	average width (µm)	+/-	nb	AWP
BKK30560	*pckA*	2.685	1.187	0.120	102	1.156
BKK30460	*ytrA*	2.676	1.187	0.130	108	1.156
BKK03170	*ycgK*	2.668	1.191	0.111	325	1.160
BKK03700	*gerKA*	2.667	1.196	0.090	126	1.165
BKK26039	*yqbN*	2.667	1.208	0.103	545	1.177
BKK13640	*spo0E*	2.664	1.241	0.155	58	1.208
BKK13860	*ykvY*	2.664	1.197	0.151	329	1.166
BKK01140	*ybaC*	2.661	1.195	0.112	228	1.164
BKK32290	*yutF*	2.652	1.192	0.116	377	1.161
BKK08440	*yfiY*	2.651	1.240	0.172	67	1.208
BKK09880	*yhaR*	2.641	1.208	0.103	153	1.177
BKK21300	*yomM*	2.633	1.181	0.105	141	1.151
BKK09100	*cspB*	2.629	1.208	0.098	129	1.177
BKK27650	*secDF*	2.629	1.254	0.084	135	1.222
BKK00180	*tadA*	2.627	1.208	0.098	98	1.177
BKK31310	*yugP*	2.625	1.186	0.112	75	1.156
BKK21460	*bdbA*	2.622	1.181	0.120	280	1.151
BKK30010	*ythP*	2.621	1.186	0.096	92	1.156
BKK08120	*yfjF*	2.617	1.189	0.110	158	1.159
BKK04540	*ydbO*	2.616	1.233	0.097	318	1.201
BKK15120	*yllA*	2.614	1.210	0.126	188	1.179
BKK03880	*yczG*	2.606	1.195	0.104	478	1.165
BKK28070	*comC*	2.606	1.190	0.116	242	1.160
BKK31040	*yuaD*	2.603	1.186	0.117	118	1.156
BKK07120	*lplC*	2.602	1.240	0.154	538	1.208
BKK18380	*iseA*	2.601	1.238	0.146	84	1.206
BKK40570	*yybO*	2.599	1.197	0.115	141	1.166
BKK22060	*pbuX*	2.598	1.169	0.122	97	1.139
BKK27200	*yrhG*	2.596	1.207	0.097	296	1.177
BKK18140	*ynfE*	2.595	1.202	0.124	213	1.172
BKK40250	*yycR*	2.594	1.177	0.110	255	1.147
BKK21060	*yonK*	2.593	1.185	0.113	539	1.155
BKK25180	*trmK*	2.58	1.181	0.113	344	1.151
BKK10110	*pbpF*	2.579	1.207	0.110	107	1.177
BKK04320	*ydaO*	2.576	1.232	0.070	42	1.201
BKK29420	*ytkK*	2.576	1.000	0.061	39	0.975
BKK39380	*hutG*	2.576	1.195	0.136	88	1.165
BKK28240	*ysoA*	2.573	1.189	0.103	185	1.160
BKK29190	*pfkA*	2.573	1.142	0.099	199	1.113
BKK15850	*sdaAB*	2.564	1.143	0.077	79	1.114
BKK01870	*ybcH*	2.559	1.000	0.090	59	0.975
BKK26450	*yrkN*	2.559	1.183	0.119	334	1.153
BKK04610	*ydcA*	2.55	1.195	0.101	135	1.165
BKK02380	*ybgB*	2.546	1.159	0.086	63	1.130
BKK11549	*yizD*	2.543	1.213	0.106	84	1.183
BKK04100	*kipR*	2.536	1.194	0.080	126	1.165
BKK17100	*pksC*	2.535	1.193	0.106	145	1.164
BKK28810	*abnA*	2.533	1.168	0.104	123	1.139
BKK09240	*yhcW*	2.532	1.207	0.107	99	1.177
BKK03190	*ycgL*	2.53	1.159	0.128	72	1.130
BKK24410	*spoIIIAC*	2.53	1.237	0.122	84	1.206
BKK19710	*yodQ*	2.527	1.158	0.134	190	1.129
BKK26190	*yqaT*	2.527	1.180	0.135	334	1.151
BKK03800	*yclN*	2.52	1.232	0.152	603	1.201
BKK33700	*opuBD*	2.515	1.163	0.094	94	1.134
BKK13490	*htpX*	2.507	1.196	0.129	69	1.166
BKK08400	*yfiU*	2.499	1.197	0.121	249	1.168
BKK23401	*spoVAEA*	2.495	1.237	0.158	95	1.206
BKK01540	*salA*	2.494	1.206	0.108	231	1.177
BKK24060	*lpdV*	2.494	1.217	0.116	246	1.187
BKK19680	*yozE*	2.486	1.157	0.124	673	1.129
BKK07370	*yfmR*	2.485	1.238	0.174	111	1.208
BKK28050	*maf*	2.476	1.188	0.095	158	1.160
BKK25320	*yqfG*	2.474	1.194	0.111	400	1.165
BKK29310	*ytnJ*	2.466	1.141	0.093	67	1.113
BKK21210	*yomV*	2.463	1.183	0.121	218	1.155
BKK09140	*yhcM*	2.46	1.206	0.108	110	1.177
BKK14380	*fruR*	2.458	1.208	0.156	69	1.179
BKK34230	*epsN*	2.455	1.163	0.114	198	1.135
BKK39140	*yxiK*	2.453	1.193	0.123	112	1.165
BKK00390	*yabD*	2.448	1.167	0.137	58	1.139
BKK31990	*dhbC*	2.448	1.186	0.093	116	1.158
BKK27729	*yrzS*	2.446	1.188	0.101	429	1.160
BKK15840	*yloV*	2.437	1.141	0.096	124	1.114
BKK30960	*glgD*	2.436	1.184	0.098	87	1.156
BKK23890	*oxaAB*	2.434	1.236	0.193	31	1.206
BKK04440	*dctB*	2.43	1.193	0.117	267	1.165
BKK35190	*yvkC*	2.43	1.163	0.094	354	1.135
BKK09750	*sspB*	2.429	1.206	0.094	76	1.177

BKK name[1]	gene	screening delta[2] (%)	average width (µm)	+/-	nb	AWP
BKK11100	*nprB*	2.429	1.212	0.120	122	1.183
BKK20330	*yorM*	2.428	1.190	0.102	219	1.162
BKK01520	*ybaK*	2.427	0.999	0.056	30	0.975
BKK36170	*ywqL*	2.427	1.141	0.139	31	1.114
BKK36630	*ywnA*	2.427	1.175	0.113	275	1.147
BKK34370	*epsA*	2.426	1.163	0.083	192	1.135
BKK34480	*yvdT*	2.426	1.163	0.083	192	1.135
BKK21229	*youA*	2.423	1.183	0.105	382	1.155
BKK35580	*tuaD*	2.416	1.177	0.124	516	1.149
BKK14350	*yknX*	2.415	1.165	0.092	154	1.138
BKK16190	*flgC*	2.412	1.191	0.106	426	1.163
BKK26770	*yrdB*	2.411	1.205	0.103	262	1.177
BKK31810	*yuzE*	2.411	1.176	0.120	271	1.148
BKK20929	*yoyI*	2.41	1.183	0.112	505	1.155
BKK31460	*kapB*	2.409	1.176	0.127	31	1.148
BKK21220	*yomU*	2.407	1.179	0.119	403	1.151
BKK03350	*yciB*	2.389	1.230	0.104	61	1.201
BKK13940	*ykwB*	2.385	1.190	0.112	280	1.163
BKK38940	*yxjI*	2.385	1.192	0.124	88	1.165
BKK00450	*sspF*	2.384	1.191	0.101	123	1.164
BKK31920	*yukF*	2.384	1.175	0.126	89	1.148
BKK10790	*asnO*	2.382	1.211	0.116	85	1.183
BKK13290	*ykzD*	2.377	1.190	0.118	220	1.163
BKK31430	*yugE*	2.375	1.175	0.128	61	1.148
BKK26990	*yraD*	2.374	1.205	0.105	205	1.177
BKK37160	*rpoE*	2.37	1.145	0.118	331	1.119
BKK07320	*yfnC*	2.354	1.237	0.153	522	1.208
BKK06980	*yesP*	2.349	1.165	0.101	30	1.138
BKK08930	*cspR*	2.349	1.190	0.114	213	1.163
BKK18810	*yobA*	2.347	1.235	0.138	54	1.206
BKK03440	*tlpC*	2.344	1.187	0.127	413	1.160
BKK09220	*yhcU*	2.344	1.205	0.111	165	1.177
BKK37670	*ywfI*	2.339	1.212	0.113	98	1.184
BKK02660	*ycbU*	2.334	0.998	0.098	50	0.975
BKK02410	*ybgG*	2.326	1.156	0.088	126	1.130
BKK38990	*scoA*	2.326	1.192	0.107	74	1.165
BKK36980	*ywlA*	2.315	1.184	0.114	126	1.158
BKK07100	*lplA*	2.314	1.236	0.119	105	1.208
BKK05520	*ydzH*	2.313	1.194	0.083	333	1.167
BKK38970	*yxjF*	2.312	1.193	0.107	82	1.166
BKK05470	*ydfM*	2.31	1.229	0.099	95	1.201
BKK33800	*opuCD*	2.309	1.176	0.115	336	1.149
BKK20830	*yopN*	2.307	1.182	0.122	343	1.155
BKK31279	*yuzH*	2.303	1.183	0.112	152	1.156
BKK00410	*rnmV*	2.3	1.190	0.117	75	1.164
BKK33490	*copB*	2.298	1.188	0.112	291	1.161
BKK37420	*albF*	2.296	1.193	0.109	119	1.166
BKK17280	*ymaD*	2.295	1.190	0.117	96	1.164
BKK12650	*xkdK*	2.292	1.216	0.152	131	1.189
BKK03300	*nasD*	2.29	1.156	0.132	58	1.130
BKK25360	*yqfC*	2.29	1.190	0.103	151	1.164
BKK08110	*sspH*	2.289	1.185	0.122	149	1.159
BKK24980	*pstC*	2.289	1.177	0.124	413	1.151
BKK40580	*yybN*	2.288	1.193	0.136	109	1.166
BKK36510	*amtB*	2.286	1.176	0.108	331	1.149
BKK12290	*ndh*	2.285	1.216	0.127	932	1.189
BKK28230	*tig*	2.284	1.186	0.101	191	1.160
BKK02240	*mpr*	2.283	1.156	0.113	144	1.130
BKK35230	*swrAA*	2.282	1.161	0.116	321	1.135
BKK09080	*yhcH*	2.281	1.195	0.121	420	1.168
BKK00590	*yabO*	2.271	1.198	0.128	138	1.172
BKK36440	*ywoH*	2.271	1.184	0.092	98	1.158
BKK37520	*ywhD*	2.271	1.193	0.109	91	1.166
BKK28400	*ysmB*	2.268	1.186	0.091	267	1.160
BKK40850	*maa*	2.264	1.211	0.105	118	1.184
BKK10230	*yhfH*	2.262	1.204	0.116	112	1.177
BKK38890	*yxjN*	2.261	1.191	0.111	166	1.165
BKK20610	*yoqJ*	2.255	1.181	0.135	124	1.155
BKK26650	*czcD*	2.254	1.203	0.105	277	1.177
BKK29805	*ytpS*	2.254	1.250	0.055	36	1.222
BKK36050	*cotB*	2.252	1.184	0.094	106	1.158
BKK31970	*dhbB*	2.25	1.174	0.110	112	1.148
BKK06540	*yezC*	2.248	1.205	0.130	198	1.179
BKK17830	*yndM*	2.243	1.190	0.110	80	1.164
BKK25780	*arsC*	2.241	1.177	0.122	361	1.151
BKK07780	*yfkR*	2.238	1.185	0.122	258	1.159
BKK12140	*yjgA*	2.234	1.216	0.159	160	1.189
BKK23530	*spoIIM*	2.231	1.179	0.115	310	1.153
BKK35780	*lytD*	2.227	1.175	0.123	251	1.149

BKK name[1]	gene	screening delta[2] (%)	average width (µm)	+/-	nb	AWP
BKK40700	*yybB*	2.224	1.211	0.092	101	1.184
BKK34470	*yveA*	2.223	1.187	0.108	461	1.161
BKK04020	*yczH*	2.219	1.191	0.079	160	1.165
BKK24600	*sinI*	2.218	0.997	0.082	103	0.975
BKK07660	*yflJ*	2.217	1.184	0.131	307	1.159
BKK34930	*hisZ*	2.217	1.192	0.109	191	1.166
BKK04190	*ydaD*	2.216	1.191	0.106	383	1.165
BKK10990	*yitH*	2.214	1.209	0.122	132	1.183
BKK01510	*ybaJ*	2.211	0.997	0.066	53	0.975
BKK12590	*xkdE*	2.21	1.215	0.188	155	1.189
BKK15320	*sigE*	2.207	1.205	0.122	164	1.179
BKK05430	*ydfJ*	2.205	1.228	0.096	75	1.201
BKK04400	*gsiB*	2.204	1.191	0.094	188	1.165
BKK32620	*yurQ*	2.203	1.159	0.109	110	1.134
BKK19380	*yojO*	2.202	1.154	0.125	293	1.129
BKK22100	*kdgA*	2.202	1.125	0.123	117	1.100
BKK34330	*epsE*	2.202	1.192	0.111	214	1.166
BKK17110	*pksD*	2.2	1.189	0.106	174	1.164
BKK04030	*ycsD*	2.19	1.185	0.117	345	1.160
BKK31980	*dhbE*	2.188	1.187	0.105	422	1.161
BKK25770	*spoIVCA*	2.185	1.249	0.090	124	1.222
BKK37980	*ywdF*	2.183	1.143	0.114	141	1.119
BKK31510	*yufK*	2.182	1.164	0.093	67	1.139
BKK12890	*ykcC*	2.176	1.235	0.151	56	1.208
BKK06580	*yerC*	2.169	1.204	0.111	334	1.179
BKK13000	*ykfD*	2.169	1.188	0.117	283	1.163
BKK40574	*yyzJ*	2.168	1.181	0.098	139	1.156
BKK13880	*glcT*	2.165	1.192	0.127	241	1.166
BKK38610	*yxaF*	2.163	1.190	0.099	56	1.165
BKK13480	*ykrK*	2.162	1.192	0.148	66	1.166
BKK39200	*yxzC*	2.157	1.190	0.131	96	1.165
BKK37450	*ywhK*	2.156	1.172	0.113	303	1.147
BKK01450	*ybxA*	2.154	1.189	0.096	175	1.164
BKK08540	*yfhI*	2.153	1.234	0.155	165	1.208
BKK08899	*ygzD*	2.146	1.202	0.086	107	1.177
BKK34940	*yvpB*	2.146	1.159	0.105	429	1.135
BKK39150	*yxiJ*	2.144	1.190	0.132	66	1.165
BKK11799	*yjzK*	2.142	1.214	0.086	338	1.189
BKK37550	*ywhA*	2.139	1.143	0.103	168	1.119
BKK08160	*yfjB*	2.138	1.184	0.112	284	1.159
BKK37210	*ywjC*	2.138	1.143	0.110	111	1.119
BKK23950	*yqjA*	2.137	1.178	0.120	335	1.153
BKK28490	*uvrC*	2.136	0.996	0.066	43	0.975
BKK24570	*gcvT*	2.135	1.212	0.095	102	1.187
BKK31410	*yugG*	2.135	1.172	0.097	63	1.148
BKK10720	*gerPA*	2.132	1.209	0.099	170	1.183
BKK05180	*ydeF*	2.123	1.192	0.100	251	1.167
BKK07980	*pdaA*	2.122	1.183	0.103	419	1.159
BKK20990	*yonU*	2.12	1.179	0.111	447	1.155
BKK07550	*yflT*	2.115	1.183	0.114	217	1.159
BKK20390	*yorG*	2.115	1.186	0.102	481	1.162
BKK18010	*yneN*	2.113	1.188	0.137	83	1.164
BKK20030	*yosR*	2.113	1.153	0.160	73	1.129
BKK18970	*yobI*	2.109	1.186	0.103	478	1.162
BKK04390	*ydbA*	2.105	1.184	0.114	397	1.160
BKK15520	*pyrAB*	2.104	1.180	0.106	63	1.156
BKK26160	*yqbC*	2.1	1.201	0.123	146	1.177
BKK07220	*yetL*	2.097	0.995	0.086	60	0.975
BKK24730	*comGA*	2.096	1.178	0.121	248	1.153
BKK06860	*yezE*	2.092	1.204	0.127	476	1.179
BKK38020	*pdxK*	2.092	1.181	0.112	101	1.157
BKK23150	*resA*	2.088	1.232	0.182	124	1.206
BKK37480	*ywhH*	2.088	1.142	0.096	370	1.119
BKK16220	*fliG*	2.081	1.187	0.100	283	1.163
BKK38560	*licH*	2.081	1.189	0.137	35	1.165
BKK40160	*yydH*	2.079	1.191	0.145	85	1.166
BKK06839	*phrH*	2.077	1.203	0.113	285	1.179
BKK35480	*yviA*	2.077	1.137	0.139	71	1.114
BKK23610	*nudF*	2.075	1.212	0.090	174	1.187
BKK15960	*ylqB*	2.074	1.142	0.113	31	1.119
BKK31550	*yufO*	2.072	1.247	0.071	88	1.222
BKK08230	*catD*	2.071	1.233	0.130	100	1.208
BKK13340	*ykoM*	2.065	1.190	0.094	172	1.166
BKK02500	*ycbG*	2.062	1.184	0.113	369	1.160
BKK02160	*ybfA*	2.059	1.153	0.108	167	1.130
BKK09570	*yhdR*	2.057	1.201	0.114	78	1.177
BKK00440	*veg*	2.054	1.187	0.102	171	1.164
BKK40730	*yyaS*	2.054	1.209	0.101	132	1.184
BKK02470	*ycbD*	2.053	1.153	0.135	95	1.130

Sup. Table 4: Cell width of mutants of the BKK collection (continued)

BKK name [1]	gene	screening delta^2 (%)	average width (µm)	+/-	nb	AWP
BKK39160	*yxiI*	2.052	1.189	0.110	101	1.165
BKK17120	*pksE*	2.051	1.231	0.150	206	1.206
BKK16390	*flhA*	2.049	1.177	0.132	387	1.153
BKK06970	*yesO*	2.048	1.226	0.081	234	1.201
BKK25820	*yqcI*	2.046	1.177	0.119	230	1.153
BKK04940	*yddE*	2.045	1.191	0.118	503	1.167
BKK29530	*sppA*	2.041	1.163	0.097	191	1.139
BKK12229	*yjzI*	2.039	1.213	0.089	1324	1.189
BKK13040	*hmp*	2.038	1.190	0.117	451	1.166
BKK17230	*pksS*	2.036	1.187	0.115	151	1.164
BKK27070	*levD*	2.036	1.201	0.093	261	1.177
BKK11680	*thiS*	2.032	1.213	0.150	755	1.189
BKK35650	*lytR*	2.031	1.173	0.106	529	1.149
BKK03000	*opuAC*	2.03	1.226	0.065	146	1.201
BKK05590	*ydgD*	2.027	1.191	0.077	159	1.167
BKK16080	*ylqH*	2.026	1.183	0.126	259	1.160
BKK27310	*pbpI*	2.024	0.995	0.106	123	0.975
BKK30450	*ytrB*	2.023	1.174	0.117	246	1.151
BKK26910	*yraK*	2.022	1.200	0.106	204	1.177
BKK04660	*ndoA*	2.02	1.188	0.092	72	1.165
BKK36110	*ywrC*	2.013	1.190	0.131	93	1.166
BKK39470	*yxeP*	2.013	1.188	0.107	95	1.165
BKK29910	*ytzH*	2.011	1.135	0.135	46	1.113
BKK26250	*yqaN*	2.007	1.200	0.103	195	1.177
BKK40540	*yybR*	2.006	1.179	0.105	120	1.156
BKK12710	*xkdR*	2.005	1.213	0.154	142	1.189
BKK23470	*spoIIAA*	2.001	1.231	0.157	184	1.206
BKK26790	*aadK*	1.996	1.200	0.101	286	1.177
BKK39440	*yxxB*	1.993	1.231	0.109	192	1.206
BKK40660	*yybF*	1.993	1.190	0.100	407	1.167
BKK20928	*yoyH*	1.992	1.178	0.110	326	1.155
BKK14440	*panE*	1.99	1.203	0.116	210	1.179
BKK22030	*ypbR*	1.99	1.176	0.114	377	1.153
BKK22720	*cheR*	1.99	1.176	0.120	252	1.153
BKK27810	*yrbD*	1.98	1.183	0.092	212	1.160
BKK33221	*yvrH*	1.976	0.994	0.081	41	0.975
BKK33990	*yvbU*	1.976	1.157	0.126	72	1.134
BKK03590	*tcyC*	1.974	1.225	0.085	153	1.201
BKK07130	*lplD*	1.974	1.202	0.118	423	1.179
BKK17580	*xynB*	1.972	1.186	0.112	205	1.164
BKK21780	*yplP*	1.97	1.178	0.130	274	1.155
BKK11190	*argC*	1.966	1.207	0.106	73	1.183
BKK37750	*ywfA*	1.965	1.172	0.112	349	1.149
BKK31520	*yufL*	1.964	1.170	0.114	329	1.148
BKK22880	*ypfD*	1.962	1.176	0.114	318	1.153
BKK15800	*thiN*	1.96	1.157	0.113	318	1.135
BKK34780	*yvcI*	1.96	1.157	0.145	339	1.135
BKK30630	*ytkD*	1.959	1.179	0.090	123	1.156
BKK24000	*bmrU*	1.958	1.210	0.104	414	1.187
BKK29220	*ytsJ*	1.958	1.162	0.099	103	1.139
BKK34070	*yvfT*	1.955	1.172	0.120	289	1.149
BKK34580	*yvdJ*	1.954	1.172	0.102	502	1.149
BKK08850	*ssuC*	1.95	1.232	0.132	128	1.208
BKK03180	*cah*	1.948	1.182	0.124	632	1.160
BKK03930	*gdh*	1.944	1.188	0.086	175	1.165
BKK40830	*yyaK*	1.942	1.207	0.103	172	1.184
BKK33470	*bdbC*	1.941	1.189	0.104	160	1.166
BKK17490	*ynzG*	1.939	1.184	0.107	433	1.162
BKK19110	*yobW*	1.939	1.194	0.116	89	1.172
BKK10130	*hemH*	1.938	1.178	0.105	92	1.156
BKK36490	*ywoC*	1.938	1.189	0.095	81	1.166
BKK14130	*ykuL*	1.937	1.189	0.142	774	1.166
BKK28360	*ysnA*	1.937	1.173	0.118	288	1.151
BKK40500	*rplI*	1.935	1.207	0.099	105	1.184
BKK10020	*serC*	1.934	1.200	0.119	157	1.177
BKK19030	*yobO*	1.934	1.194	0.139	98	1.172
BKK30680	*ytjA*	1.932	1.178	0.113	78	1.156
BKK11120	*yitT*	1.926	1.185	0.112	223	1.163
BKK21170	*yomZ*	1.923	1.177	0.104	316	1.155
BKK33580	*yvaF*	1.922	1.156	0.125	114	1.134
BKK22640	*trpB*	1.921	1.121	0.096	196	1.100
BKK13770	*ykvO*	1.919	1.189	0.130	68	1.166
BKK21420	*bhlA*	1.919	1.173	0.111	216	1.151
BKK21800	*ypkP*	1.918	1.177	0.117	566	1.155
BKK10010	*trpP*	1.917	1.232	0.127	46	1.208
BKK23810	*yqjN*	1.912	1.210	0.102	350	1.187
BKK20590	*yoqL*	1.91	1.177	0.122	249	1.155
BKK00600	*yabP*	1.909	1.169	0.107	550	1.147
BKK13690	*motA*	1.898	1.189	0.102	117	1.166

BKK name [1]	gene	screening delta^2 (%)	average width (µm)	+/-	nb	AWP
BKK23540	*yqkK*	1.898	1.210	0.110	283	1.187
BKK03200	*ycgM*	1.892	1.151	0.119	36	1.130
BKK10360	*yhfT*	1.891	1.206	0.101	217	1.183
BKK32630	*yurR*	1.889	1.156	0.092	150	1.134
BKK18490	*rtp*	1.888	1.184	0.126	266	1.162
BKK11900	*yjcL*	1.885	1.184	0.120	287	1.163
BKK19340	*yocR*	1.884	1.179	0.095	155	1.158
BKK27730	*ruvB*	1.884	1.181	0.107	314	1.160
BKK14990	*ylbF*	1.883	1.201	0.131	615	1.179
BKK28250	*leuD*	1.88	1.181	0.099	224	1.160
BKK35170	*uvrB*	1.876	1.156	0.117	87	1.135
BKK32990	*mrgA*	1.873	1.156	0.091	90	1.134
BKK10780	*yisN*	1.871	1.205	0.130	152	1.183
BKK13780	*ykvP*	1.871	1.188	0.121	416	1.166
BKK35950	*rbsC*	1.867	1.188	0.105	116	1.166
BKK24140	*prpC*	1.865	1.209	0.102	659	1.187
BKK26660	*yrdN*	1.857	1.199	0.117	403	1.177
BKK38630	*katX*	1.855	1.186	0.131	34	1.165
BKK04860	*ydcQ*	1.852	1.186	0.121	95	1.165
BKK29540	*ppnKB*	1.851	1.161	0.089	140	1.139
BKK06440	*purB*	1.849	1.201	0.124	179	1.179
BKK19470	*yojF*	1.847	1.190	0.118	412	1.168
BKK27220	*yrhE*	1.843	1.198	0.100	228	1.177
BKK14300	*moaE*	1.838	1.201	0.107	880	1.179
BKK17760	*yndE*	1.836	1.185	0.113	206	1.164
BKK39270	*bglP*	1.835	1.186	0.113	79	1.165
BKK01090	*rplGB*	1.832	1.185	0.109	249	1.164
BKK05420	*ydfI*	1.824	1.223	0.116	68	1.201
BKK07330	*yfnB*	1.818	1.200	0.112	548	1.179
BKK13830	*ykvU*	1.816	1.184	0.115	326	1.163
BKK31390	*yugI*	1.816	1.169	0.105	232	1.148
BKK00970	*yacP*	1.815	1.181	0.123	307	1.160
BKK10740	*yisJ*	1.814	1.205	0.113	75	1.183
BKK02480	*gudP*	1.81	1.223	0.069	197	1.201
BKK32790	*yusG*	1.81	1.155	0.095	171	1.134
BKK13800	*ykvR*	1.804	1.187	0.132	58	1.166
BKK05770	*ydhI*	1.798	1.188	0.110	439	1.167
BKK02490	*gudD*	1.797	1.150	0.123	43	1.130
BKK25930	*yqcE*	1.796	1.172	0.104	256	1.151
BKK31050	*gbsB*	1.796	1.177	0.126	106	1.156
BKK33480	*bdbD*	1.796	1.155	0.095	157	1.134
BKK17590	*xylR*	1.79	1.184	0.113	81	1.164
BKK04520	*ydbM*	1.788	1.180	0.115	696	1.160
BKK21599	*yoyK*	1.788	1.176	0.107	459	1.155
BKK40370	*yycJ*	1.785	1.150	0.098	131	1.130
BKK40610	*yybK*	1.784	1.187	0.121	101	1.166
BKK15560	*pyrE*	1.783	1.160	0.092	111	1.139
BKK38830	*aldY*	1.781	1.185	0.118	77	1.165
BKK38950	*yxjH*	1.78	1.178	0.094	70	1.158
BKK03610	*tcyA*	1.775	1.168	0.116	422	1.147
BKK16140	*codV*	1.77	1.205	0.125	65	1.184
BKK19140	*yozB*	1.768	1.182	0.116	362	1.162
BKK08070	*acoB*	1.767	1.179	0.118	417	1.159
BKK09310	*pgcA*	1.767	1.230	0.127	38	1.208
BKK02710	*yczC*	1.765	1.223	0.067	82	1.201
BKK36150	*ywqN*	1.764	1.134	0.087	54	1.114
BKK40840	*yyaJ*	1.764	1.188	0.117	192	1.167
BKK04420	*ydbC*	1.763	1.185	0.100	263	1.165
BKK02170	*ybfB*	1.758	1.168	0.120	442	1.147
BKK39280	*yxxE*	1.757	1.185	0.122	80	1.165
BKK14010	*cheV*	1.754	1.183	0.123	333	1.163
BKK02310	*ybfO*	1.752	1.150	0.097	86	1.130
BKK08830	*ssuB*	1.745	1.229	0.167	96	1.208
BKK13530	*kinE*	1.743	1.188	0.103	359	1.168
BKK18690	*yoaP*	1.743	1.182	0.114	392	1.162
BKK10610	*yhjR*	1.74	1.204	0.108	150	1.183
BKK21240	*yomS*	1.739	1.175	0.123	411	1.155
BKK29430	*ytzD*	1.739	1.159	0.092	208	1.139
BKK29610	*ezrA*	1.739	1.132	0.116	65	1.113
BKK03100	*ycgG*	1.737	1.150	0.107	110	1.130
BKK02000	*ybdJ*	1.735	1.150	0.097	72	1.130
BKK04490	*ydbJ*	1.734	1.222	0.098	109	1.201
BKK07490	*yfmF*	1.733	1.179	0.094	296	1.159
BKK10890	*yisX*	1.732	1.204	0.120	111	1.183
BKK05320	*ydeS*	1.726	1.187	0.122	143	1.167
BKK06700	*yerO*	1.725	1.199	0.126	373	1.179
BKK06073	*ydzW*	1.722	1.167	0.098	121	1.147
BKK29700	*acuB*	1.721	1.178	0.105	142	1.158
BKK24210	*yqiG*	1.719	1.207	0.145	583	1.187

BKK name [1]	gene	screening delta^2 (%)	average width (µm)	+/-	nb	AWP
BKK13120	*proB*	1.714	1.186	0.123	109	1.166
BKK28740	*araP*	1.713	1.159	0.102	107	1.139
BKK04700	*rsbU*	1.71	1.185	0.115	93	1.165
BKK10500	*yhjG*	1.71	1.204	0.102	100	1.183
BKK27350	*yrrN*	1.698	1.171	0.123	232	1.151
BKK29490	*tpx*	1.695	1.132	0.120	128	1.113
BKK40578	*yyzK*	1.692	1.186	0.114	161	1.166
BKK04170	*ydaB*	1.687	1.185	0.097	343	1.165
BKK22160	*yptA*	1.68	1.119	0.097	30	1.100
BKK03060	*lctP*	1.674	1.149	0.121	51	1.130
BKK19689	*yokU*	1.674	1.188	0.106	330	1.168
BKK24290	*xseB*	1.672	1.207	0.112	260	1.187
BKK10600	*yhjQ*	1.665	1.203	0.120	85	1.183
BKK30940	*glgP*	1.665	1.175	0.103	132	1.156
BKK09400	*spoVR*	1.663	1.228	0.112	201	1.208
BKK16950	*pbpX*	1.661	1.227	0.160	62	1.206
BKK21380	*yomF*	1.661	1.174	0.125	384	1.155
BKK18410	*ggt*	1.66	1.157	0.097	89	1.138
BKK20090	*yosK*	1.656	1.148	0.113	70	1.129
BKK28300	*ilvH*	1.656	1.179	0.096	313	1.160
BKK04180	*ydaC*	1.655	1.184	0.087	146	1.165
BKK16170	*codY*	1.649	1.182	0.138	230	1.163
BKK39350	*hutH*	1.647	1.184	0.115	102	1.165
BKK09260	*yhxA*	1.643	1.182	0.104	262	1.163
BKK09120	*yhcK*	1.636	1.182	0.108	295	1.163
BKK30840	*yteA*	1.632	1.175	0.116	117	1.156
BKK06890	*cotJA*	1.631	1.198	0.113	433	1.179
BKK25190	*cccA*	1.63	1.172	0.111	473	1.153
BKK39680	*iolI*	1.621	1.166	0.106	353	1.147
BKK06500	*purM*	1.62	1.198	0.121	369	1.179
BKK36060	*cotH*	1.619	1.168	0.118	334	1.149
BKK36550	*spoIIQ*	1.619	1.168	0.118	334	1.149
BKK35690	*ggaA*	1.617	1.176	0.105	68	1.158
BKK04370	*ydaS*	1.61	1.184	0.094	466	1.165
BKK08550	*sspK*	1.601	1.177	0.114	318	1.159
BKK10340	*phoE*	1.601	1.202	0.115	140	1.183
BKK17460	*glnA*	1.601	1.182	0.115	59	1.164
BKK24540	*yqhL*	1.6	1.206	0.110	223	1.187
BKK06330	*yeaC*	1.596	1.198	0.120	155	1.179
BKK27140	*oatA*	1.596	1.242	0.068	82	1.222
BKK32860	*yusN*	1.596	1.152	0.102	110	1.134
BKK10970	*yitF*	1.591	1.202	0.119	107	1.183
BKK20180	*yosB*	1.589	1.180	0.114	343	1.162
BKK35840	*ywtF*	1.589	1.132	0.100	277	1.114
BKK00220	*yaaL*	1.586	1.202	0.124	98	1.183
BKK17040	*mutS*	1.584	1.182	0.112	258	1.164
BKK39060	*citH*	1.584	1.183	0.114	87	1.165
BKK39290	*yxxD*	1.584	1.185	0.121	66	1.166
BKK17980	*sspP*	1.582	1.182	0.111	159	1.164
BKK05870	*gmuF*	1.581	1.186	0.116	431	1.167
BKK22180	*gpsB*	1.577	1.118	0.115	76	1.100
BKK06048	*ydzU*	1.574	1.186	0.113	280	1.167
BKK30580	*ytmA*	1.574	1.174	0.085	99	1.156
BKK31540	*yufN*	1.571	1.241	0.082	57	1.222
BKK32980	*yusZ*	1.569	1.180	0.115	395	1.161
BKK09350	*yhdB*	1.568	1.195	0.105	280	1.177
BKK05700	*ydhC*	1.558	1.185	0.075	162	1.167
BKK03920	*glcU*	1.555	1.220	0.082	32	1.201
BKK34680	*yvcT*	1.554	1.153	0.107	60	1.135
BKK19050	*yobQ*	1.551	1.190	0.106	142	1.172
BKK29330	*ytmO*	1.551	1.130	0.108	791	1.113
BKK12340	*uxuA*	1.549	1.207	0.092	1324	1.189
BKK11170	*yitY*	1.547	1.202	0.098	110	1.183
BKK30330	*ytvB*	1.547	1.174	0.089	111	1.156
BKK23900	*yqjF*	1.546	1.225	0.164	94	1.206
BKK09650	*yhdZ*	1.545	1.195	0.110	122	1.177
BKK17990	*sspO*	1.543	1.180	0.124	356	1.162
BKK39740	*iolC*	1.542	0.990	0.076	90	0.975
BKK28670	*ysfB*	1.541	1.157	0.096	170	1.139
BKK34250	*epsL*	1.538	1.153	0.093	142	1.135
BKK07870	*yfkK*	1.537	1.180	0.120	342	1.163
BKK09420	*lytE*	1.537	1.227	0.115	39	1.208
BKK16590	*ylxS*	1.535	1.179	0.127	326	1.161
BKK22869	*ypzI*	1.535	1.117	0.088	110	1.100
BKK08650	*fabL*	1.533	1.177	0.155	135	1.159
BKK40130	*yydK*	1.531	1.165	0.095	388	1.147
BKK12960	*dppE*	1.529	1.155	0.093	140	1.138
BKK09380	*nsrR*	1.528	1.195	0.104	120	1.177
BKK08029	*yfzA*	1.524	1.176	0.110	516	1.159

BKK name [1]	gene	screening delta^2 (%)	average width (µm)	+/-	nb	AWP
BKK05230	*ydeK*	1.523	1.157	0.085	95	1.139
BKK27610	*apt*	1.518	1.177	0.098	142	1.160
BKK02090	*ybxI*	1.516	1.147	0.129	111	1.130
BKK08760	*spo0M*	1.514	1.227	0.147	248	1.208
BKK37590	*ywgA*	1.514	1.136	0.131	72	1.119
BKK25010	*yqgE*	1.512	1.225	0.116	49	1.206
BKK29840	*ytpP*	1.511	1.157	0.103	98	1.139
BKK23050	*fmnP*	1.509	1.117	0.103	148	1.100
BKK00210	*recR*	1.502	1.165	0.163	503	1.147
BKK21990	*ypdQ*	1.502	1.172	0.103	484	1.155
BKK06910	*cotJC*	1.5	1.197	0.115	261	1.179
BKK35420	*yvyG*	1.5	1.131	0.108	862	1.114
BKK40950	*yyaC*	1.498	1.202	0.096	139	1.184
BKK04840	*ydcO*	1.495	1.177	0.131	359	1.160
BKK24610	*sinR*	1.492	1.202	0.088	162	1.184
BKK11660	*tenI*	1.49	1.207	0.118	264	1.189
BKK30660	*ytkA*	1.486	1.240	0.055	32	1.222
BKK12970	*ykfA*	1.484	1.226	0.139	121	1.208
BKK33450	*helD*	1.476	1.151	0.085	97	1.134
BKK05620	*ydgF*	1.475	1.177	0.113	690	1.160
BKK19630	*deoD*	1.472	1.146	0.119	548	1.129
BKK19430	*yojJ*	1.469	1.146	0.132	269	1.129
BKK20820	*yopO*	1.465	1.172	0.106	450	1.155
BKK13010	*ykgB*	1.462	1.183	0.126	555	1.166
BKK10710	*gerPB*	1.461	1.201	0.117	127	1.183
BKK16800	*spoIIIE*	1.461	1.224	0.175	62	1.206
BKK26810	*yrpC*	1.454	1.240	0.084	165	1.222
BKK01660	*ybbD*	1.451	1.219	0.083	66	1.201
BKK11390	*appB*	1.451	1.226	0.162	190	1.208
BKK06150	*gutB*	1.447	1.196	0.126	82	1.179
BKK32160	*yutM*	1.446	1.178	0.110	434	1.161
BKK33720	*opuBB*	1.445	1.183	0.114	181	1.166
BKK32500	*pucB*	1.444	1.183	0.114	156	1.166
BKK32300	*yutE*	1.441	1.178	0.113	384	1.161
BKK35810	*gerBB*	1.435	1.130	0.116	31	1.114
BKK25860	*yqcG*	1.431	1.168	0.107	300	1.151
BKK27280	*yrrT*	1.431	0.989	0.085	47	0.975
BKK33590	*yvaG*	1.43	1.151	0.107	178	1.134
BKK26020	*yqbP*	1.427	1.193	0.122	119	1.177
BKK19120	*czrA*	1.426	1.188	0.109	157	1.172
BKK40630	*yybI*	1.426	1.201	0.109	194	1.184
BKK07570	*yflS*	1.422	1.226	0.140	73	1.208
BKK34240	*epsM*	1.421	1.151	0.108	130	1.135
BKK08530	*yfhH*	1.417	1.175	0.106	257	1.159
BKK17220	*pksR*	1.415	1.180	0.108	157	1.164
BKK28910	*lrgA*	1.404	1.178	0.108	203	1.161
BKK34080	*yvfS*	1.404	1.166	0.097	241	1.149
BKK40120	*yyzE*	1.398	0.989	0.063	46	0.975
BKK06740	*yefB*	1.394	1.195	0.113	251	1.179
BKK28040	*radC*	1.394	1.169	0.118	183	1.153
BKK35860	*pgdS*	1.393	1.165	0.103	527	1.149
BKK34120	*ganB*	1.391	1.177	0.106	416	1.161
BKK14310	*moaD*	1.386	1.195	0.107	574	1.179
BKK24430	*spoIIIAA*	1.386	1.167	0.113	452	1.151
BKK40590	*yybM*	1.383	1.182	0.107	186	1.166
BKK08950	*yhbE*	1.382	1.225	0.165	85	1.208
BKK14009	*ykzT*	1.382	1.182	0.130	124	1.166
BKK09000	*yhbJ*	1.381	1.193	0.123	131	1.177
BKK27820	*yrbC*	1.38	1.169	0.114	470	1.153
BKK25370	*yqfB*	1.378	0.988	0.083	49	0.975
BKK07440	*yfmK*	1.372	1.175	0.106	308	1.159
BKK32810	*yusI*	1.366	1.150	0.103	65	1.134
BKK37030	*racA*	1.362	1.173	0.121	116	1.157
BKK24820	*yqgU*	1.361	1.179	0.106	322	1.164
BKK03830	*yclQ*	1.36	1.218	0.091	69	1.201
BKK25050	*yqgA*	1.36	1.169	0.115	206	1.153
BKK36560	*ywnH*	1.36	1.182	0.103	162	1.166
BKK05370	*ydfD*	1.355	1.175	0.128	409	1.160
BKK01590	*ybaS*	1.353	1.153	0.107	123	1.138
BKK22070	*xpt*	1.352	1.155	0.112	112	1.139
BKK02520	*ycbJ*	1.348	1.145	0.089	215	1.130
BKK08980	*yhbH*	1.347	1.193	0.106	183	1.177
BKK29290	*ytnL*	1.346	1.155	0.092	133	1.139
BKK05100	*yddT*	1.34	1.183	0.112	351	1.167
BKK32440	*pucK*	1.339	1.163	0.110	68	1.148
BKK17600	*xylA*	1.333	1.179	0.138	65	1.164
BKK26089	*yqzN*	1.329	1.192	0.118	191	1.177
BKK31830	*yueE*	1.328	1.163	0.111	251	1.148
BKK19650	*yodM*	1.325	1.144	0.118	1093	1.129

Sup. Table 4: Cell width of mutants of the BKK collection (continued)

BKK name [1]	gene	screening delta[2] (%)	average width (µm)	+/-	nb	AWP
BKK02630	*tatAD*	1.323	1.217	0.079	43	1.201
BKK21920	*ugtP*	1.322	1.222	0.162	73	1.206
BKK13230	*thiV*	1.317	1.224	0.144	59	1.208
BKK11710	*thiD*	1.316	1.205	0.097	800	1.189
BKK33980	*yvbT*	1.311	1.177	0.117	449	1.161
BKK35400	*flgL*	1.309	1.150	0.127	456	1.135
BKK35990	*ywrO*	1.308	1.129	0.093	92	1.114
BKK26370	*yqaC*	1.306	1.192	0.098	415	1.177
BKK07190	*yezD*	1.304	1.194	0.120	279	1.179
BKK14290	*mobB*	1.304	1.195	0.098	513	1.179
BKK41020	*trmE*	1.303	1.175	0.113	189	1.160
BKK29860	*ytoP*	1.301	1.128	0.120	71	1.113
BKK10320	*yhfP*	1.298	1.178	0.106	390	1.163
BKK22020	*ypbS*	1.298	1.115	0.131	151	1.100
BKK15440	*ylyA*	1.297	1.154	0.088	127	1.139
BKK24960	*pstBA*	1.293	1.222	0.123	132	1.206
BKK05150	*ydeC*	1.292	1.173	0.117	96	1.158
BKK24530	*yqhM*	1.29	1.202	0.120	251	1.187
BKK19360	*odhB*	1.287	1.144	0.121	549	1.129
BKK37090	*ywjI*	1.282	1.162	0.108	254	1.147
BKK38260	*efeN*	1.277	1.181	0.110	63	1.166
BKK02250	*ybfJ*	1.275	1.217	0.068	77	1.201
BKK35310	*yvyD*	1.272	1.150	0.124	178	1.135
BKK22090	*kdgT*	1.27	1.222	0.126	196	1.206
BKK30620	*ytlD*	1.269	1.238	0.081	34	1.222
BKK03530	*ycxA*	1.267	1.217	0.115	381	1.201
BKK18180	*yngB*	1.266	1.222	0.167	172	1.206
BKK04040	*ycsE*	1.264	1.174	0.120	687	1.160
BKK35970	*ywsB*	1.263	1.128	0.143	32	1.114
BKK24520	*mntR*	1.262	1.168	0.118	145	1.153
BKK05529	*ydzR*	1.26	0.987	0.076	37	0.975
BKK15110	*ylbQ*	1.26	1.194	0.129	310	1.179
BKK15100	*ylbP*	1.259	1.171	0.107	130	1.156
BKK19060	*yobR*	1.258	1.199	0.139	119	1.184
BKK05570	*ydgB*	1.25	1.182	0.127	169	1.167
BKK14960	*ylbC*	1.249	1.194	0.116	99	1.179
BKK11090	*yitR*	1.244	1.177	0.102	427	1.163
BKK29620	*hisJ*	1.242	1.127	0.106	116	1.113
BKK30160	*ytcQ*	1.242	1.237	0.132	33	1.222
BKK20200	*yorZ*	1.238	1.176	0.118	242	1.162
BKK21360	*yomH*	1.238	1.169	0.108	660	1.155
BKK30190	*bioI*	1.237	1.170	0.111	146	1.156
BKK36940	*ywlD*	1.237	0.987	0.067	60	0.975
BKK18470	*proJ*	1.233	1.186	0.107	362	1.172
BKK21560	*yokK*	1.232	1.168	0.113	374	1.153
BKK06319	*ydzX*	1.231	1.193	0.111	263	1.179
BKK11740	*cotZ*	1.229	1.177	0.114	369	1.163
BKK32540	*bsn*	1.223	1.153	0.085	138	1.139
BKK06120	*ydjB*	1.222	1.153	0.103	162	1.139
BKK29690	*acuA*	1.222	1.153	0.091	97	1.139
BKK03070	*mdr*	1.221	1.216	0.071	60	1.201
BKK32570	*frlD*	1.218	0.987	0.085	108	0.975
BKK18330	*ppsB*	1.217	1.186	0.104	265	1.172
BKK03660	*yclE*	1.216	1.152	0.084	77	1.138
BKK10580	*yhjO*	1.213	1.223	0.167	120	1.208
BKK35960	*rbsB*	1.21	1.175	0.108	324	1.161
BKK07390	*yfmP*	1.206	1.173	0.122	115	1.159
BKK03210	*ycgN*	1.205	1.144	0.117	102	1.130
BKK12830	*spoIISA*	1.205	1.182	0.112	386	1.168
BKK23670	*yqkA*	1.203	1.165	0.111	373	1.151
BKK40780	*tetL*	1.202	1.161	0.100	434	1.147
BKK37400	*albD*	1.199	1.180	0.104	91	1.166
BKK02770	*yccK*	1.193	1.173	0.111	306	1.160
BKK27090	*aapA*	1.193	1.191	0.095	644	1.177
BKK08450	*yfiZ*	1.191	0.987	0.077	110	0.975
BKK32390	*yunF*	1.19	1.162	0.104	91	1.148
BKK33880	*yvbJ*	1.19	0.987	0.073	46	0.975
BKK23330	*ypuC*	1.187	1.201	0.097	299	1.187
BKK11550	*yjbH*	1.185	1.197	0.107	164	1.183
BKK18890	*yobF*	1.181	1.151	0.087	139	1.138
BKK24110	*yqzF*	1.181	1.167	0.120	340	1.153
BKK30480	*ytqA*	1.179	1.170	0.119	97	1.156
BKK31800	*yueF*	1.174	1.161	0.113	30	1.148
BKK07580	*citS*	1.172	1.223	0.135	97	1.208
BKK23340	*ypuB*	1.172	1.201	0.096	492	1.187
BKK19200	*desR*	1.17	1.185	0.081	187	1.172
BKK13700	*clpE*	1.166	1.180	0.171	37	1.166
BKK02210	*ybfH*	1.165	1.143	0.101	173	1.130
BKK39670	*iolJ*	1.165	1.178	0.100	128	1.165

BKK name [1]	gene	screening delta[2] (%)	average width (µm)	+/-	nb	AWP
BKK19690	*kamA*	1.164	1.142	0.121	516	1.129
BKK30690	*ytiB*	1.163	1.169	0.101	118	1.156
BKK34450	*sacB*	1.163	1.148	0.118	344	1.135
BKK18310	*ppsD*	1.159	1.151	0.074	48	1.138
BKK24420	*spoIIIAB*	1.155	1.220	0.143	44	1.206
BKK20999	*yoyJ*	1.154	1.168	0.101	696	1.155
BKK37790	*rocG*	1.141	1.132	0.104	77	1.119
BKK22580	*ypiB*	1.14	1.113	0.101	432	1.100
BKK20840	*yopM*	1.138	1.167	0.109	463	1.153
BKK35120	*yvlB*	1.138	1.148	0.095	221	1.135
BKK11600	*yjbM*	1.13	1.202	0.123	874	1.189
BKK08500	*yfhE*	1.128	1.172	0.107	303	1.159
BKK25610	*yqeM*	1.125	1.177	0.086	116	1.164
BKK09860	*khtT*	1.124	1.222	0.149	81	1.208
BKK21230	*yomT*	1.118	1.168	0.121	384	1.155
BKK02400	*ybgF*	1.117	1.143	0.092	116	1.130
BKK06077	*ydzW*	1.114	1.171	0.098	123	1.158
BKK34530	*cotR*	1.114	1.148	0.101	139	1.135
BKK06300	*cotA*	1.113	1.192	0.137	231	1.179
BKK39100	*yxiO*	1.113	1.220	0.122	240	1.206
BKK19040	*csaA*	1.103	1.185	0.105	258	1.172
BKK29300	*rbfK*	1.1	1.152	0.102	129	1.139
BKK13090	*ykkC*	1.099	1.222	0.147	54	1.208
BKK15870	*recG*	1.094	1.170	0.106	87	1.157
BKK33070	*gerAC*	1.092	1.147	0.090	128	1.134
BKK19900	*yotF*	1.09	1.170	0.105	101	1.158
BKK12270	*yjlB*	1.086	1.202	0.139	1310	1.189
BKK03320	*nasB*	1.083	1.172	0.122	628	1.160
BKK14080	*ykuH*	1.083	1.179	0.121	496	1.166
BKK31530	*yufM*	1.082	1.164	0.123	167	1.151
BKK20160	*yosD*	1.078	1.141	0.145	94	1.129
BKK18770	*cyeA*	1.073	1.150	0.093	165	1.138
BKK23230	*ypuF*	1.071	1.200	0.109	87	1.187
BKK12620	*xkdH*	1.07	1.202	0.146	325	1.189
BKK32420	*pucR*	1.068	1.174	0.104	189	1.161
BKK08130	*yfjE*	1.06	0.985	0.086	35	0.975
BKK27340	*yrrO*	1.056	1.163	0.119	280	1.151
BKK34090	*yvfR*	1.056	1.162	0.114	355	1.149
BKK26800	*yrpB*	1.054	1.189	0.104	219	1.177
BKK30640	*ytkC*	1.053	1.150	0.100	144	1.138
BKK39930	*yxaM*	1.051	1.162	0.102	406	1.149
BKK32200	*yutJ*	1.05	1.151	0.091	155	1.139
BKK03160	*ycgJ*	1.049	1.172	0.103	409	1.160
BKK21290	*yomN*	1.047	1.167	0.102	284	1.155
BKK30230	*bioK*	1.047	1.163	0.122	231	1.151
BKK23730	*yqjV*	1.044	1.199	0.107	514	1.187
BKK09250	*yhcX*	1.04	1.189	0.106	161	1.177
BKK18820	*yobB*	1.038	1.174	0.114	363	1.162
BKK39340	*hutP*	1.031	1.177	0.110	128	1.165
BKK26210	*yqaR*	1.027	1.165	0.113	220	1.153
BKK06037	*ydzT*	1.026	1.179	0.126	384	1.167
BKK39330	*abnB*	1.022	1.159	0.097	385	1.147
BKK37780	*rocA*	1.02	1.159	0.119	283	1.147
BKK14520	*adeC*	1.018	1.191	0.134	117	1.179
BKK21250	*yomR*	1.016	1.167	0.109	564	1.155
BKK21050	*yonN*	1.015	1.167	0.111	523	1.155
BKK02120	*ybeC*	1.014	1.141	0.091	47	1.130
BKK08330	*yfiN*	1.011	1.171	0.101	487	1.159
BKK17410	*cwlC*	1.01	1.219	0.172	262	1.206
BKK27080	*levR*	1.009	1.189	0.102	272	1.177
BKK26380	*yqaB*	1.008	1.189	0.105	524	1.177
BKK05710	*ydhD*	1.005	1.214	0.085	97	1.201
BKK39690	*iolH*	1.004	1.176	0.107	125	1.165
BKK32720	*yurZ*	1.003	1.146	0.109	150	1.134
BKK02830	*ycdF*	1.001	1.171	0.097	372	1.160
BKK18670	*yoaN*	1.001	1.149	0.087	138	1.138
BKK28340	*ysnF*	1.001	1.171	0.102	308	1.160
BKK31260	*mcpB*	1	1.173	0.113	392	1.161
BKK27440	*glnH*	0.995	1.171	0.111	306	1.160
BKK16330	*cheY*	0.994	1.173	0.112	242	1.162
BKK29390	*ytmI*	0.992	1.173	0.112	387	1.161
BKK04800	*ydcL*	0.989	1.159	0.119	400	1.147
BKK27840	*safA*	0.988	1.165	0.122	303	1.153
BKK11210	*argB*	0.985	1.195	0.107	73	1.183
BKK34190	*yvfH*	0.985	1.146	0.112	621	1.135
BKK39220	*yxxG*	0.984	1.176	0.119	107	1.165
BKK27880	*nifS*	0.982	1.162	0.113	333	1.151
BKK14880	*ctaB*	0.981	1.191	0.144	304	1.179
BKK18980	*yobJ*	0.981	1.173	0.107	322	1.162

BKK name [1]	gene	screening delta[2] (%)	average width (µm)	+/-	nb	AWP
BKK39210	*yxiF*	0.98	1.176	0.087	93	1.165
BKK04120	*yczI*	0.979	1.176	0.071	157	1.165
BKK17530	*ynaE*	0.978	1.173	0.109	333	1.162
BKK34410	*yveG*	0.977	1.146	0.127	133	1.135
BKK40060	*gntK*	0.976	1.179	0.123	198	1.168
BKK05170	*ydeE*	0.975	0.985	0.090	172	0.975
BKK15470	*pyrR*	0.975	1.151	0.074	139	1.139
BKK27060	*levE*	0.973	1.162	0.119	322	1.151
BKK11880	*metC*	0.972	1.201	0.109	76	1.189
BKK33170	*yvrB*	0.971	1.145	0.072	109	1.134
BKK05300	*ydeQ*	0.969	1.171	0.111	442	1.160
BKK24750	*yqhB*	0.966	1.175	0.101	199	1.164
BKK22150	*ypvA*	0.965	1.111	0.129	31	1.100
BKK30890	*ytxO*	0.965	1.167	0.102	119	1.156
BKK37410	*albE*	0.963	1.161	0.113	302	1.149
BKK09280	*glpF*	0.962	1.179	0.100	204	1.168
BKK11860	*yjcH*	0.962	1.200	0.130	1045	1.189
BKK13390	*ykoT*	0.962	1.178	0.160	133	1.166
BKK07050	*yesW*	0.956	1.171	0.118	723	1.160
BKK25910	*yqxH*	0.951	1.169	0.093	161	1.158
BKK30510	*ytpA*	0.951	1.167	0.088	123	1.156
BKK14100	*ykuJ*	0.949	1.150	0.096	157	1.139
BKK33050	*gerAA*	0.948	1.145	0.108	98	1.134
BKK27460	*glnP*	0.944	1.234	0.069	98	1.222
BKK04140	*pbpC*	0.941	1.213	0.074	38	1.201
BKK35930	*rbsD*	0.94	1.160	0.109	441	1.149
BKK27540	*yrvM*	0.937	1.164	0.118	194	1.153
BKK18510	*yoxC*	0.936	1.183	0.106	142	1.172
BKK34340	*epsD*	0.935	1.177	0.099	149	1.166
BKK32840	*fadN*	0.933	1.145	0.109	126	1.134
BKK29990	*pbuO*	0.932	1.233	0.098	43	1.222
BKK40080	*gntZ*	0.93	1.179	0.112	275	1.168
BKK40470	*yycC*	0.929	1.177	0.122	74	1.166
BKK36540	*ywnJ*	0.925	1.125	0.114	142	1.114
BKK20410	*yorE*	0.921	1.173	0.108	274	1.162
BKK30500	*ytpB*	0.921	1.167	0.117	135	1.156
BKK28060	*spoIIB*	0.918	1.233	0.069	47	1.222
BKK02580	*ycbO*	0.912	1.140	0.092	111	1.130
BKK08260	*yfiG*	0.912	1.219	0.123	61	1.208
BKK08460	*yfhA*	0.912	1.179	0.110	271	1.168
BKK11929	*yjzG*	0.91	1.173	0.124	238	1.163
BKK15690	*rpoZ*	0.907	1.145	0.113	146	1.134
BKK35500	*degS*	0.907	1.195	0.096	368	1.184
BKK21080	*yonI*	0.894	1.165	0.111	263	1.155
BKK38170	*qoxA*	0.892	1.167	0.111	118	1.157
BKK06230	*iolT*	0.891	1.212	0.099	51	1.201
BKK30350	*yttB*	0.891	1.233	0.104	53	1.222
BKK29600	*braB*	0.888	1.150	0.097	141	1.139
BKK32220	*yutI*	0.888	1.158	0.116	96	1.148
BKK07910	*yfkF*	0.887	1.173	0.111	307	1.163
BKK18720	*yoaR*	0.887	1.172	0.117	408	1.162
BKK27430	*glnQ*	0.885	1.233	0.073	63	1.222
BKK33270	*yvrO*	0.885	1.177	0.092	100	1.166
BKK02850	*adcA*	0.882	1.212	0.185	62	1.201
BKK28530	*etfB*	0.88	1.170	0.104	54	1.160
BKK15810	*spoVM*	0.875	1.217	0.143	136	1.206
BKK18849	*yozV*	0.872	1.148	0.096	136	1.138
BKK12500	*xkdA*	0.87	1.199	0.131	581	1.189
BKK21180	*yomY*	0.87	1.165	0.105	121	1.155
BKK30710	*ythA*	0.869	1.171	0.110	517	1.161
BKK32380	*yunE*	0.869	1.233	0.088	187	1.222
BKK30659	*ytzI*	0.864	1.166	0.099	112	1.156
BKK29630	*yttP*	0.863	1.161	0.103	266	1.151
BKK40022	*yxaC*	0.862	1.159	0.118	436	1.149
BKK29940	*ytlR*	0.859	1.166	0.091	104	1.156
BKK13620	*mtnD*	0.858	1.173	0.106	247	1.163
BKK05670	*ydgJ*	0.856	1.177	0.083	202	1.167
BKK03680	*yclG*	0.855	1.148	0.083	150	1.138
BKK39540	*yxeI*	0.853	1.176	0.161	61	1.166
BKK17680	*thyA*	0.843	1.172	0.102	391	1.162
BKK07735	*yflB*	0.842	1.169	0.116	352	1.159
BKK06170	*ydjE*	0.841	1.169	0.109	242	1.160
BKK11490	*yjbC*	0.84	1.193	0.108	104	1.183
BKK30990	*thiT*	0.839	1.232	0.144	449	1.222
BKK18110	*ynfC*	0.838	1.181	0.097	86	1.172
BKK26490	*yrkJ*	0.836	1.163	0.118	313	1.153
BKK37280	*narG*	0.836	1.128	0.095	66	1.119
BKK06480	*purL*	0.835	1.157	0.105	230	1.147
BKK30135	*yteP*	0.834	1.167	0.105	100	1.158

BKK name [1]	gene	screening delta[2] (%)	average width (µm)	+/-	nb	AWP
BKK32770	*yusE*	0.831	1.144	0.103	127	1.134
BKK15250	*ylxW*	0.829	1.178	0.106	230	1.168
BKK29160	*ytvI*	0.829	0.983	0.092	59	0.975
BKK35050	*yvnA*	0.828	1.171	0.104	484	1.161
BKK38330	*lrgB*	0.825	1.176	0.115	146	1.166
BKK17020	*ymcA*	0.823	1.173	0.121	89	1.164
BKK06240	*bdhA*	0.822	1.189	0.118	208	1.179
BKK14560	*defB*	0.822	1.189	0.111	157	1.179
BKK23180	*spmA*	0.821	1.161	0.110	214	1.151
BKK33310	*fhuB*	0.821	1.176	0.089	132	1.166
BKK16260	*ylxF*	0.819	1.172	0.105	385	1.163
BKK19000	*yobL*	0.818	1.171	0.109	228	1.162
BKK05540	*ydfS*	0.817	1.157	0.108	443	1.147
BKK16870	*ymfI*	0.815	1.173	0.127	152	1.164
BKK23960	*artR*	0.815	1.147	0.126	64	1.138
BKK34440	*pbpE*	0.815	1.159	0.104	531	1.149
BKK17180	*pksJ*	0.813	1.173	0.138	53	1.164
BKK03940	*ycnI*	0.808	1.167	0.103	145	1.158
BKK16940	*recA*	0.808	1.171	0.136	314	1.162
BKK02440	*glnJ*	0.805	1.139	0.095	145	1.130
BKK36820	*atpG*	0.805	1.157	0.108	161	1.147
BKK15490	*pyrB*	0.801	1.165	0.095	92	1.156
BKK16890	*ymfK*	0.801	1.173	0.116	110	1.164
BKK38160	*qoxB*	0.8	1.157	0.106	300	1.147
BKK20940	*yopC*	0.797	1.164	0.106	347	1.155
BKK23740	*yqjU*	0.796	1.197	0.101	476	1.187
BKK04260	*topB*	0.794	1.169	0.124	161	1.160
BKK13070	*ykkA*	0.794	1.176	0.091	75	1.166
BKK36140	*ywqO*	0.792	1.123	0.129	34	1.114
BKK38670	*yxlE*	0.791	1.174	0.100	71	1.165
BKK04970	*yddH*	0.788	1.167	0.103	187	1.158
BKK15040	*ylbK*	0.786	1.188	0.099	57	1.179
BKK35980	*ywsA*	0.785	1.166	0.104	214	1.157
BKK12170	*yjgD*	0.782	1.198	0.171	663	1.189
BKK05810	*gmuB*	0.781	1.176	0.070	262	1.167
BKK04550	*ydbP*	0.78	1.174	0.115	38	1.165
BKK16980	*spoVS*	0.78	1.171	0.114	489	1.162
BKK39110	*deaD*	0.78	1.174	0.108	71	1.165
BKK14930	*ctaG*	0.778	1.188	0.084	340	1.179
BKK38250	*ywbO*	0.775	1.166	0.128	73	1.157
BKK20570	*yoqN*	0.773	1.138	0.129	216	1.129
BKK22430	*panB*	0.77	1.148	0.093	106	1.139
BKK05030	*yddM*	0.763	1.168	0.114	341	1.160
BKK07210	*yetK*	0.763	1.177	0.114	305	1.168
BKK21270	*yomP*	0.759	1.164	0.106	512	1.155
BKK34350	*epsC*	0.759	1.158	0.105	493	1.149
BKK18000	*citB*	0.758	1.171	0.116	333	1.162
BKK23200	*ypuI*	0.758	1.196	0.113	175	1.187
BKK14690	*yktD*	0.757	1.188	0.123	159	1.179
BKK38220	*ywcC*	0.755	1.127	0.129	194	1.119
BKK39910	*yxnB*	0.754	1.173	0.116	114	1.165
BKK02650	*pcp*	0.751	1.156	0.099	407	1.147
BKK04110	*lipC*	0.749	1.168	0.123	363	1.160
BKK33150	*yvqK*	0.749	1.143	0.105	183	1.134
BKK04690	*rsbT*	0.747	1.174	0.125	153	1.165
BKK17480	*ynzF*	0.747	1.171	0.107	549	1.162
BKK38760	*cydA*	0.746	1.173	0.112	80	1.165
BKK28150	*hemC*	0.744	1.168	0.122	118	1.160
BKK12810	*xlyA*	0.738	1.171	0.124	192	1.163
BKK37730	*bacB*	0.738	1.158	0.110	383	1.149
BKK16880	*ymfJ*	0.737	1.172	0.115	163	1.164
BKK25800	*yqcK*	0.735	1.162	0.119	297	1.153
BKK20920	*yopE*	0.734	1.163	0.093	358	1.155
BKK32340	*lytH*	0.734	1.231	0.068	103	1.222
BKK22670	*trpD*	0.727	1.108	0.100	210	1.100
BKK34860	*hisI*	0.727	1.143	0.118	365	1.135
BKK08750	*ygxA*	0.722	1.171	0.117	280	1.163
BKK07300	*yfnE*	0.721	0.982	0.107	90	0.975
BKK17310	*ymaG*	0.72	1.172	0.103	209	1.164
BKK08220	*yfiC*	0.719	1.217	0.149	146	1.208
BKK20790	*yopR*	0.719	1.163	0.110	540	1.155
BKK10330	*yhfQ*	0.717	1.171	0.101	412	1.163
BKK04839	*ydzL*	0.716	1.173	0.101	120	1.165
BKK21370	*yomG*	0.713	1.163	0.115	493	1.155
BKK24190	*yqiI*	0.713	1.215	0.141	36	1.206
BKK39139	*yxzJ*	0.713	1.173	0.106	60	1.165
BKK10810	*yisP*	0.711	1.192	0.109	100	1.183
BKK38980	*scoB*	0.71	1.175	0.111	95	1.166
BKK07310	*yfnD*	0.706	1.187	0.117	483	1.179

Sup. Table 4: Cell width of mutants of the BKK collection (continued)

BKK name [1]	gene	screening delta^2 (%)	average width (µm)	+/-	nb	AWP
BKK22950	*ypdA*	0.704	1.108	0.122	68	1.100
BKK16280	*flgD*	0.7	1.176	0.110	238	1.168
BKK19980	*yosW*	0.699	1.170	0.113	242	1.162
BKK12010	*manP*	0.698	1.197	0.110	207	1.189
BKK24400	*spoIIIAD*	0.697	1.215	0.156	73	1.206
BKK00760	*pabC*	0.694	1.166	0.108	91	1.158
BKK13760	*ykvN*	0.694	1.171	0.104	317	1.163
BKK19940	*yotB*	0.694	1.170	0.112	401	1.162
BKK39310	*yxiC*	0.693	1.173	0.095	154	1.165
BKK20620	*yoqI*	0.69	1.170	0.123	243	1.162
BKK30060	*ytfP*	0.688	1.164	0.097	179	1.156
BKK07410	*yfmN*	0.686	1.167	0.117	156	1.159
BKK12539	*ykzK*	0.685	1.157	0.127	328	1.149
BKK12600	*xkdF*	0.684	1.174	0.139	219	1.166
BKK38620	*aag*	0.684	1.173	0.118	86	1.165
BKK12030	*yjdF*	0.683	1.217	0.121	62	1.208
BKK27110	*yrhO*	0.681	1.185	0.110	374	1.177
BKK27230	*yrhD*	0.681	1.185	0.110	374	1.177
BKK33460	*yvgT*	0.681	1.142	0.102	219	1.134
BKK31670	*yuxO*	0.677	1.156	0.143	105	1.148
BKK38910	*yxjL*	0.677	1.215	0.133	72	1.206
BKK02260	*ybfK*	0.676	1.167	0.107	479	1.160
BKK02940	*yceH*	0.672	1.138	0.090	112	1.130
BKK05000	*yddK*	0.668	1.167	0.120	413	1.160
BKK18050	*yneQ*	0.668	1.170	0.120	382	1.162
BKK34000	*cyeB*	0.665	1.157	0.102	296	1.149
BKK21120	*yonE*	0.662	1.163	0.103	431	1.155
BKK17750	*yndD*	0.657	1.171	0.099	143	1.164
BKK24020	*bmrR*	0.655	1.161	0.113	349	1.153
BKK24230	*spoIVB*	0.651	1.214	0.151	171	1.206
BKK28140	*hemD*	0.649	1.167	0.115	32	1.160
BKK30670	*luxS*	0.648	1.164	0.092	146	1.156
BKK12630	*xkdI*	0.643	1.197	0.115	243	1.189
BKK37850	*spsG*	0.643	1.126	0.106	156	1.119
BKK01480	*truA*	0.642	1.167	0.109	987	1.160
BKK03970	*ycnL*	0.64	1.172	0.075	138	1.165
BKK02700	*estA*	0.637	1.209	0.069	46	1.201
BKK40690	*yybC*	0.637	1.192	0.082	150	1.184
BKK25530	*spoIIP*	0.634	1.230	0.122	228	1.222
BKK39640	*yxdL*	0.634	1.230	0.123	41	1.222
BKK18460	*gltC*	0.633	1.145	0.104	86	1.138
BKK09070	*yhcG*	0.632	1.184	0.119	349	1.177
BKK39570	*yxeF*	0.632	1.230	0.084	41	1.222
BKK37970	*ung*	0.628	1.126	0.117	234	1.119
BKK05400	*ydfG*	0.627	1.175	0.090	283	1.167
BKK10800	*yizA*	0.627	1.191	0.116	78	1.183
BKK24250	*ahrC*	0.626	1.194	0.115	224	1.187
BKK06074	*ydzW*	0.624	1.175	0.131	184	1.167
BKK15650	*yloB*	0.624	1.141	0.087	176	1.134
BKK22570	*ypiF*	0.623	1.107	0.115	586	1.100
BKK08190	*malR*	0.622	1.166	0.120	268	1.159
BKK29570	*sspA*	0.62	1.120	0.105	74	1.113
BKK14650	*yktB*	0.618	1.186	0.131	229	1.179
BKK38200	*galK*	0.615	1.155	0.101	480	1.147
BKK17699	*ynzK*	0.614	1.171	0.101	59	1.164
BKK21450	*yolJ*	0.614	1.158	0.108	327	1.151
BKK40860	*yyaH*	0.611	1.192	0.115	101	1.184
BKK07450	*yfmJ*	0.604	1.166	0.110	189	1.159
BKK23390	*spoVAF*	0.602	1.214	0.144	33	1.206
BKK39490	*yxeN*	0.597	1.145	0.103	70	1.138
BKK02450	*glnL*	0.596	1.167	0.118	302	1.160
BKK12050	*yjdH*	0.596	1.196	0.132	53	1.189
BKK02360	*nagBB*	0.593	1.137	0.109	196	1.130
BKK08140	*yfjD*	0.591	1.166	0.104	594	1.159
BKK00420	*ksgA*	0.585	1.154	0.103	542	1.147
BKK12610	*xkdG*	0.585	1.196	0.089	288	1.189
BKK16020	*rimM*	0.584	1.163	0.115	58	1.156
BKK11470	*oppF*	0.58	1.169	0.115	270	1.163
BKK16299	*ylzI*	0.576	1.169	0.124	264	1.163
BKK36870	*atpB*	0.568	1.173	0.101	96	1.166
BKK38000	*ywdD*	0.568	1.125	0.083	111	1.119
BKK40380	*yycI*	0.565	1.136	0.096	121	1.130
BKK00890	*yacL*	0.563	1.154	0.107	520	1.147
BKK30310	*ytwF*	0.563	1.163	0.099	107	1.156
BKK15880	*fapR*	0.562	1.125	0.126	861	1.119
BKK38030	*ywdA*	0.562	1.125	0.095	98	1.119
BKK21400	*yomD*	0.558	1.161	0.101	255	1.155
BKK30530	*ytnA*	0.558	1.162	0.103	83	1.156
BKK31340	*yugM*	0.553	1.144	0.094	147	1.138

BKK name [1]	gene	screening delta^2 (%)	average width (µm)	+/-	nb	AWP
BKK14790	*ylaI*	0.552	1.186	0.101	798	1.179
BKK36930	*ywlE*	0.551	1.163	0.095	139	1.157
BKK19060	*yobR*	0.546	1.144	0.096	95	1.138
BKK32550	*yurJ*	0.544	1.146	0.091	112	1.139
BKK00880	*disA*	0.541	1.144	0.128	65	1.138
BKK19090	*yobU*	0.541	1.168	0.116	346	1.162
BKK29950	*ytlQ*	0.541	1.162	0.090	85	1.156
BKK16780	*rnjB*	0.54	1.170	0.103	100	1.164
BKK05910	*ydiB*	0.539	1.174	0.094	138	1.167
BKK17200	*pksM*	0.538	1.170	0.123	99	1.164
BKK33440	*cysJ*	0.538	1.140	0.123	146	1.134
BKK22310	*recU*	0.533	1.106	0.126	173	1.100
BKK06640	*yerI*	0.531	1.185	0.138	221	1.179
BKK14030	*ykuC*	0.53	1.213	0.164	91	1.206
BKK35590	*tuaC*	0.53	1.172	0.112	139	1.166
BKK13250	*ykoG*	0.529	1.173	0.098	350	1.166
BKK12100	*yjeA*	0.526	1.169	0.111	403	1.163
BKK24550	*gcvPB*	0.522	1.193	0.116	252	1.187
BKK04880	*ydcS*	0.517	1.171	0.133	55	1.165
BKK03750	*yclJ*	0.515	1.171	0.090	172	1.165
BKK31380	*yuzA*	0.512	1.154	0.097	152	1.148
BKK36620	*ywnB*	0.508	1.153	0.102	370	1.147
BKK15069	*ylzH*	0.506	1.145	0.123	68	1.139
BKK30470	*ytzC*	0.505	1.162	0.102	111	1.156
BKK39960	*yxaI*	0.505	1.171	0.101	113	1.165
BKK38050	*sacP*	0.503	1.124	0.113	151	1.119
BKK11350	*yjaZ*	0.502	1.168	0.108	254	1.163
BKK13820	*ykvT*	0.501	1.172	0.104	198	1.166
BKK32760	*yusD*	0.498	1.167	0.114	491	1.161
BKK36990	*ywkF*	0.497	1.163	0.124	81	1.157
BKK18908	*yozY*	0.495	1.144	0.094	152	1.138
BKK26510	*yrkH*	0.495	1.183	0.103	265	1.177
BKK22560	*qcrA*	0.492	1.106	0.116	509	1.100
BKK39780	*iolS*	0.491	1.170	0.099	67	1.165
BKK09640	*yhdY*	0.488	1.183	0.113	140	1.177
BKK37720	*bacC*	0.487	1.124	0.097	60	1.119
BKK16370	*fliR*	0.486	1.167	0.104	307	1.162
BKK37900	*spsB*	0.485	1.155	0.109	325	1.149
BKK19620	*yodJ*	0.484	1.212	0.137	277	1.206
BKK16090	*sucC*	0.482	1.165	0.106	324	1.160
BKK40870	*ccpB*	0.48	1.153	0.106	296	1.147
BKK18920	*phrK*	0.479	1.174	0.123	361	1.168
BKK22940	*prsW*	0.479	1.212	0.113	177	1.206
BKK08700	*ygaE*	0.475	1.164	0.116	366	1.159
BKK03720	*gerKB*	0.474	1.170	0.119	187	1.165
BKK31330	*yugN*	0.474	1.161	0.101	90	1.156
BKK26140	*yqbE*	0.472	1.157	0.107	509	1.151
BKK23519	*yqzK*	0.469	1.193	0.103	228	1.187
BKK20050	*yosQ*	0.467	1.135	0.128	244	1.129
BKK24240	*recN*	0.462	1.193	0.098	405	1.187
BKK17290	*ebrB*	0.459	1.212	0.123	103	1.206
BKK12490	*yjqC*	0.456	1.194	0.132	479	1.189
BKK23710	*polYB*	0.455	1.192	0.105	309	1.187
BKK04680	*rsbS*	0.454	1.170	0.106	190	1.165
BKK13720	*queC*	0.453	1.172	0.115	305	1.166
BKK04920	*yddC*	0.451	1.153	0.104	382	1.147
BKK18500	*yoxD*	0.441	1.167	0.116	316	1.162
BKK28680	*glcD*	0.441	1.144	0.102	258	1.139
BKK14210	*ykuT*	0.44	1.168	0.122	316	1.163
BKK32350	*yunB*	0.439	1.144	0.103	309	1.139
BKK39760	*mmsA*	0.439	1.163	0.085	196	1.158
BKK40030	*yxaB*	0.439	1.161	0.102	150	1.156
BKK16690	*pnpA*	0.437	1.167	0.103	296	1.162
BKK13160	*ohrB*	0.436	1.171	0.144	251	1.166
BKK13200	*rsbRB*	0.432	1.168	0.113	293	1.163
BKK40110	*bglA*	0.432	1.173	0.112	233	1.168
BKK02220	*ybfI*	0.43	1.135	0.092	170	1.130
BKK28510	*xsa*	0.428	1.165	0.117	121	1.160
BKK32960	*yusY*	0.423	1.171	0.100	174	1.166
BKK05270	*ydzF*	0.422	1.165	0.117	427	1.160
BKK23360	*ppiB*	0.418	1.158	0.109	475	1.153
BKK38320	*cidA*	0.418	1.154	0.114	449	1.149
BKK23380	*lysA*	0.417	1.192	0.107	296	1.187
BKK32910	*yusS*	0.417	1.139	0.099	102	1.134
BKK23850	*zwf*	0.414	1.158	0.119	278	1.153
BKK39450	*yxeR*	0.414	1.211	0.143	161	1.206
BKK17170	*pksI*	0.412	1.168	0.122	55	1.164
BKK19490	*gerT*	0.408	1.134	0.110	855	1.129
BKK16290	*flgE*	0.406	1.167	0.105	264	1.163

BKK name [1]	gene	screening delta^2 (%)	average width (µm)	+/-	nb	AWP
BKK39410	*nupC*	0.406	1.211	0.121	169	1.206
BKK39940	*yxaL*	0.406	1.169	0.120	149	1.165
BKK12320	*yjmC*	0.402	1.194	0.112	487	1.189
BKK26870	*yraN*	0.401	1.227	0.077	226	1.222
BKK00540	*yabK*	0.399	1.168	0.116	119	1.164
BKK23720	*yqzH*	0.399	1.192	0.111	149	1.187
BKK17640	*alrB*	0.398	1.211	0.140	126	1.206
BKK20900	*yopG*	0.397	1.159	0.111	293	1.155
BKK04900	*yddA*	0.394	1.170	0.097	174	1.165
BKK12069	*yjzH*	0.394	1.167	0.121	380	1.163
BKK05580	*ydgC*	0.389	1.172	0.078	186	1.167
BKK27120	*sigV*	0.389	1.181	0.111	106	1.177
BKK15500	*pyrC*	0.388	1.162	0.099	114	1.158
BKK33049	*yvzF*	0.388	1.139	0.107	137	1.134
BKK24100	*bkdR*	0.386	1.162	0.101	76	1.158
BKK36800	*atpC*	0.386	1.119	0.100	114	1.114
BKK20870	*yopJ*	0.385	1.156	0.121	241	1.151
BKK29250	*nrnA*	0.383	1.117	0.091	182	1.113
BKK06010	*ydiL*	0.381	1.172	0.093	276	1.167
BKK14970	*ylbD*	0.376	1.172	0.101	330	1.168
BKK28720	*abfA*	0.375	1.144	0.093	167	1.139
BKK34020	*yvbX*	0.375	1.139	0.089	166	1.134
BKK31360	*yugK*	0.372	1.152	0.115	238	1.148
BKK17630	*yncC*	0.371	1.211	0.179	185	1.206
BKK35340	*fliD*	0.37	1.139	0.112	342	1.135
BKK01600	*ybbA*	0.369	1.181	0.096	187	1.177
BKK27980	*spoIVFA*	0.369	1.227	0.103	37	1.222
BKK08470	*yfhB*	0.363	1.163	0.106	647	1.159
BKK04280	*ydaK*	0.359	1.169	0.102	90	1.165
BKK17140	*pksF*	0.358	1.168	0.102	125	1.164
BKK22170	*ypsC*	0.356	1.143	0.091	185	1.139
BKK22140	*kduD*	0.355	1.104	0.136	162	1.100
BKK34950	*pelC*	0.355	1.170	0.118	121	1.166
BKK03520	*srfAD*	0.349	1.206	0.110	358	1.201
BKK33190	*yvrD*	0.349	1.138	0.096	120	1.134
BKK39770	*iolR*	0.349	1.151	0.099	347	1.147
BKK21620	*yokE*	0.345	1.157	0.112	268	1.153
BKK06400	*yebE*	0.338	1.162	0.108	155	1.158
BKK14730	*ylaC*	0.337	1.183	0.164	47	1.179
BKK08350	*estB*	0.336	1.166	0.107	343	1.163
BKK31060	*gbsA*	0.336	1.162	0.101	87	1.158
BKK14120	*ykzF*	0.333	1.170	0.118	709	1.166
BKK03740	*yclI*	0.327	1.163	0.114	365	1.160
BKK33840	*yvbF*	0.322	1.138	0.093	94	1.134
BKK02290	*psd*	0.318	1.151	0.109	486	1.147
BKK04960	*yddG*	0.314	1.205	0.144	424	1.201
BKK15140	*mraW*	0.311	1.210	0.144	38	1.206
BKK20000	*yosU*	0.309	1.133	0.144	350	1.129
BKK23140	*resB*	0.309	1.191	0.104	848	1.187
BKK36840	*atpH*	0.309	1.118	0.117	116	1.114
BKK10490	*sipV*	0.308	1.166	0.109	259	1.163
BKK08610	*yfhO*	0.306	1.172	0.112	328	1.168
BKK18640	*yoaK*	0.304	1.141	0.090	121	1.138
BKK20020	*yosS*	0.303	1.133	0.130	174	1.129
BKK36720	*ywmE*	0.302	1.151	0.112	230	1.147
BKK08010	*yfjP*	0.296	1.162	0.113	362	1.159
BKK28550	*fadR*	0.294	1.141	0.088	128	1.138
BKK16270	*fliK*	0.293	1.171	0.120	278	1.168
BKK27330	*udk*	0.291	0.978	0.120	35	0.975
BKK04850	*ydcP*	0.29	1.163	0.107	520	1.160
BKK37260	*narJ*	0.29	1.122	0.102	110	1.119
BKK00840	*mcsA*	0.287	1.161	0.106	73	1.158
BKK23790	*yqjP*	0.277	1.190	0.109	238	1.187
BKK08840	*ssuA*	0.273	1.171	0.111	284	1.168
BKK17420	*spoVK*	0.273	1.210	0.144	106	1.206
BKK03340	*folEB*	0.272	1.133	0.087	153	1.130
BKK40359	*yyzO*	0.271	1.153	0.114	356	1.149
BKK32450	*pucL*	0.269	1.143	0.082	149	1.139
BKK23550	*mleA*	0.268	1.190	0.103	198	1.187
BKK08000	*yfjQ*	0.266	1.162	0.101	373	1.159
BKK33820	*opuCB*	0.266	1.137	0.091	98	1.134
BKK39610	*yxeB*	0.266	1.151	0.104	590	1.147
BKK00570	*yabM*	0.265	1.141	0.131	38	1.138
BKK27830	*coxA*	0.264	1.163	0.101	115	1.160
BKK28220	*clpX*	0.263	1.163	0.112	147	1.160
BKK15400	*ylmG*	0.258	1.182	0.156	135	1.179
BKK32780	*yusF*	0.257	1.137	0.100	178	1.134
BKK25940	*yqcD*	0.252	1.154	0.104	313	1.151
BKK24310	*folD*	0.25	1.154	0.120	106	1.151

BKK name [1]	gene	screening delta^2 (%)	average width (µm)	+/-	nb	AWP
BKK23750	*yqjT*	0.249	0.977	0.082	55	0.975
BKK33390	*yvgM*	0.249	1.141	0.099	123	1.138
BKK07600	*yflP*	0.247	1.162	0.103	326	1.159
BKK19010	*yobM*	0.246	1.174	0.088	124	1.172
BKK10730	*yisI*	0.243	1.186	0.121	118	1.183
BKK30020	*ytzE*	0.243	1.159	0.101	138	1.156
BKK22610	*tyrA*	0.242	1.103	0.142	69	1.100
BKK33400	*yvgN*	0.236	1.137	0.097	121	1.134
BKK02370	*ybgA*	0.235	1.133	0.097	68	1.130
BKK18240	*yngHA*	0.235	1.174	0.104	136	1.172
BKK05070	*yddQ*	0.232	1.162	0.107	412	1.160
BKK25340	*phoH*	0.231	1.166	0.098	211	1.164
BKK09500	*yhdK*	0.23	1.211	0.198	77	1.208
BKK10030	*hinT*	0.23	1.180	0.108	124	1.177
BKK31010	*yuaG*	0.228	1.159	0.103	99	1.156
BKK13410	*ykoV*	0.227	1.169	0.121	65	1.166
BKK14780	*ylaH*	0.226	1.182	0.104	302	1.179
BKK17570	*xynP*	0.226	1.166	0.124	87	1.164
BKK05480	*ydfN*	0.225	1.170	0.100	142	1.167
BKK26120	*yqbG*	0.225	1.179	0.090	222	1.177
BKK03790	*yclM*	0.224	1.168	0.101	695	1.165
BKK13169	*ykzN*	0.223	1.211	0.118	207	1.208
BKK20350	*yorK*	0.222	1.142	0.109	41	1.139
BKK09810	*yhaZ*	0.22	1.165	0.110	260	1.163
BKK04730	*sigB*	0.219	1.167	0.175	164	1.165
BKK31960	*dhbF*	0.219	1.169	0.112	184	1.166
BKK37070	*rpmEA*	0.217	1.160	0.098	107	1.158
BKK02550	*ycbL*	0.215	1.132	0.088	130	1.130
BKK26560	*yrkC*	0.215	1.156	0.102	583	1.153
BKK31910	*yukE*	0.215	1.142	0.101	140	1.139
BKK23650	*yqkC*	0.214	1.190	0.111	197	1.187
BKK14860	*pycA*	0.213	1.165	0.110	228	1.163
BKK30910	*cotSA*	0.213	1.158	0.095	140	1.156
BKK18860	*yozH*	0.212	1.164	0.110	370	1.162
BKK09550	*yhdP*	0.211	1.211	0.169	39	1.208
BKK10430	*yhxD*	0.211	1.186	0.119	104	1.183
BKK05750	*ydhG*	0.203	1.170	0.075	265	1.167
BKK36920	*ywlF*	0.203	1.159	0.145	30	1.157
BKK39880	*yxbC*	0.202	1.150	0.112	397	1.147
BKK40300	*rapG*	0.202	1.169	0.110	107	1.166
BKK30650	*dps*	0.201	1.158	0.115	113	1.156
BKK23170	*spmB*	0.197	1.189	0.090	186	1.187
BKK07700	*nagP*	0.196	1.161	0.113	434	1.159
BKK13260	*ykoH*	0.196	1.140	0.115	113	1.138
BKK08560	*yfhJ*	0.195	1.161	0.112	591	1.159
BKK32090	*yuiA*	0.195	1.150	0.121	401	1.148
BKK32510	*pucA*	0.192	1.150	0.098	85	1.148
BKK25030	*yqgC*	0.189	1.156	0.121	260	1.153
BKK39170	*yxzG*	0.184	1.167	0.111	78	1.165
BKK11870	*metI*	0.179	1.191	0.133	619	1.189
BKK28570	*yshE*	0.178	1.162	0.097	70	1.160
BKK34390	*pnbA*	0.177	1.137	0.108	195	1.135
BKK32320	*yutC*	0.175	1.168	0.103	241	1.166
BKK37040	*ywkB*	0.174	1.151	0.115	428	1.149
BKK33620	*yvaK*	0.167	1.136	0.096	148	1.134
BKK15080	*rpmF*	0.166	1.181	0.154	33	1.179
BKK00290	*yaaQ*	0.16	1.181	0.143	721	1.179
BKK11950	*yjcQ*	0.159	1.164	0.116	221	1.163
BKK10370	*bioY*	0.156	1.210	0.136	251	1.208
BKK33730	*opuBA*	0.155	1.163	0.114	603	1.161
BKK01389	*ybzG*	0.148	1.165	0.103	312	1.164
BKK03310	*nasC*	0.147	1.132	0.102	91	1.130
BKK01880	*ybcI*	0.145	1.140	0.108	157	1.138
BKK38800	*yxkH*	0.145	1.210	0.131	126	1.208
BKK37390	*albC*	0.144	1.151	0.103	476	1.149
BKK02050	*ybdO*	0.142	1.132	0.095	130	1.130
BKK17710	*tatAC*	0.142	1.165	0.110	135	1.164
BKK04890	*ydcT*	0.14	1.167	0.107	124	1.165
BKK24180	*yqiK*	0.138	1.155	0.117	264	1.153
BKK22169	*ypzG*	0.134	1.141	0.090	131	1.139
BKK16420	*cheB*	0.132	1.163	0.107	233	1.161
BKK33010	*cssR*	0.128	1.136	0.093	170	1.134
BKK30520	*ytoA*	0.126	1.157	0.103	133	1.156
BKK25450	*prmA*	0.125	1.165	0.086	229	1.164
BKK01970	*skfG*	0.123	1.141	0.092	231	1.139
BKK13220	*thiW*	0.123	1.210	0.185	60	1.208
BKK32970	*yusY*	0.121	1.168	0.104	140	1.166
BKK14740	*ylaD*	0.12	1.169	0.109	459	1.168
BKK11940	*yjcP*	0.117	1.190	0.102	573	1.189

Sup. Table 4: Cell width of mutants of the BKK collection (continued)

BKK name [1]	gene	screening delta[2] (%)	average width (µm)	+/-	nb	AWP
BKK22750	*menH*	0.117	1.102	0.111	167	1.100
BKK01960	*skfF*	0.116	1.159	0.110	97	1.158
BKK06260	*ydjN*	0.115	1.180	0.126	106	1.179
BKK02019	*ybzI*	0.113	1.131	0.081	88	1.130
BKK38350	*ywbE*	0.113	1.120	0.108	196	1.119
BKK36320	*ywpG*	0.112	1.158	0.106	104	1.157
BKK27100	*yrhP*	0.111	1.223	0.067	74	1.222
BKK35330	*fliS*	0.109	1.136	0.116	72	1.135
BKK14600	*pdhC*	0.108	0.976	0.067	62	0.975
BKK08940	*yhbD*	0.101	1.169	0.111	312	1.168
BKK33370	*yvgK*	0.1	1.162	0.112	358	1.161
BKK32900	*yusR*	0.099	1.135	0.098	156	1.134
BKK31890	*yukC*	0.098	1.149	0.122	33	1.148
BKK18660	*yoaM*	0.094	1.163	0.113	539	1.162
BKK10450	*yhjB*	0.092	1.169	0.100	396	1.168
BKK21510	*yolD*	0.092	1.156	0.106	445	1.155
BKK14720	*ylaB*	0.088	1.169	0.114	205	1.168
BKK30250	*ytaP*	0.088	1.157	0.101	182	1.156
BKK35890	*pgsC*	0.088	1.150	0.114	508	1.149
BKK06470	*purQ*	0.087	1.180	0.117	328	1.179
BKK17090	*pksB*	0.087	1.208	0.142	95	1.206
BKK08780	*ygaJ*	0.084	1.160	0.108	183	1.159
BKK05040	*yddN*	0.08	1.161	0.110	508	1.160
BKK11840	*yjcF*	0.08	1.190	0.102	239	1.189
BKK21630	*yokD*	0.078	1.156	0.112	537	1.155
BKK31875	*yukB*	0.077	1.223	0.085	142	1.222
BKK16230	*fliH*	0.074	1.163	0.109	309	1.163
BKK09390	*ygxB*	0.073	1.178	0.105	122	1.177
BKK10870	*yisU*	0.071	1.184	0.122	89	1.183
BKK08779	*ygzC*	0.069	0.976	0.068	78	0.975
BKK31220	*yuxG*	0.068	1.157	0.090	121	1.156
BKK07360	*yfmS*	0.067	1.209	0.162	277	1.208
BKK20500	*ligB*	0.065	1.140	0.102	119	1.139
BKK20540	*yoqR*	0.065	1.140	0.096	140	1.139
BKK35040	*yvnB*	0.064	1.136	0.099	258	1.135
BKK21940	*degR*	0.062	1.156	0.106	328	1.155
BKK15090	*ylbO*	0.061	1.180	0.128	295	1.179
BKK22120	*kdgR*	0.061	1.154	0.105	309	1.153
BKK24800	*yqgW*	0.061	1.164	0.100	306	1.164
BKK11260	*yjzC*	0.059	1.184	0.108	83	1.183
BKK14240	*rok*	0.057	1.163	0.108	254	1.163
BKK03870	*ycnE*	0.054	1.166	0.109	252	1.165
BKK34060	*yvfU*	0.053	1.135	0.093	115	1.134
BKK18850	*yobD*	0.045	1.139	0.083	33	1.138
BKK38040	*sacA*	0.045	1.167	0.119	82	1.166
BKK14910	*ctaE*	0.044	1.180	0.124	362	1.179
BKK30570	*ytmB*	0.036	1.156	0.095	100	1.156
BKK11270	*yjzD*	0.034	1.184	0.137	84	1.183
BKK31170	*yulF*	0.033	1.156	0.105	133	1.156
BKK17920	*ynzD*	0.032	1.164	0.122	83	1.164
BKK19270	*yozN*	0.031	1.172	0.105	99	1.172
BKK13110	*purU*	0.025	1.163	0.122	377	1.163
BKK31190	*yulD*	0.025	1.151	0.116	329	1.151
BKK39090	*yxiP*	0.023	1.165	0.111	167	1.165
BKK24170	*mmgA*	0.022	0.975	0.086	46	0.975
BKK16410	*ylxH*	0.021	1.156	0.093	246	1.156
BKK28710	*cstA*	0.021	1.156	0.093	246	1.156
BKK29960	*ytlP*	0.021	1.156	0.106	75	1.156
BKK12780	*xepA*	0.015	1.209	0.116	125	1.208
BKK05600	*ydgE*	0.014	1.167	0.078	333	1.167
BKK11370	*appF*	0.012	1.168	0.112	350	1.168
BKK10900	*yisY*	0.01	1.183	0.109	90	1.183
BKK11240	*carB*	0.01	1.183	0.099	77	1.183
BKK03260	*ycgS*	0.008	1.130	0.082	74	1.130
BKK16240	*fliI*	0.008	1.163	0.102	402	1.163
BKK20170	*yosC*	0.006	1.162	0.103	302	1.162
BKK02200	*ybfG*	0.005	1.160	0.111	334	1.160
BKK31280	*yugU*	0.005	1.158	0.098	182	1.158
BKK35510	*yvyE*	0.004	1.114	0.115	94	1.114
BKK33570	*yvaE*	0.003	1.222	0.096	64	1.222
BKK05380	*ydfE*	0	1.167	0.119	117	1.167
BKK38060	*ywcJ*	-0.001	1.158	0.104	159	1.158
BKK07380	*yfmQ*	-0.002	1.159	0.114	290	1.159
BKK04870	*ydcR*	-0.003	1.147	0.113	421	1.147
BKK13310	*tnrA*	-0.003	1.163	0.107	316	1.163
BKK11079	*yizB*	-0.005	1.183	0.106	129	1.183
BKK23940	*yqjB*	-0.007	1.187	0.107	578	1.187
BKK29460	*moaB*	-0.008	1.113	0.112	56	1.113
BKK35070	*yvmC*	-0.008	1.158	0.118	102	1.158

BKK name [1]	gene	screening delta[2] (%)	average width (µm)	+/-	nb	AWP
BKK33750	*sdpA*	-0.01	1.166	0.114	98	1.166
BKK03270	*ycgT*	-0.013	1.130	0.087	70	1.130
BKK19810	*ypqP*	-0.013	1.162	0.108	490	1.162
BKK03900	*gabT*	-0.016	1.159	0.116	439	1.160
BKK08280	*yfiI*	-0.016	1.159	0.109	485	1.159
BKK13020	*ykgA*	-0.017	1.166	0.113	222	1.166
BKK21980	*ypdP*	-0.017	1.155	0.104	410	1.155
BKK19080	*yobT*	-0.019	1.138	0.096	153	1.138
BKK11400	*appC*	-0.021	1.162	0.113	319	1.163
BKK09060	*yhcF*	-0.022	1.177	0.109	213	1.177
BKK40940	*yyaD*	-0.028	1.167	0.086	1044	1.167
BKK02680	*lmrA*	-0.031	1.130	0.091	136	1.130
BKK17470	*ynxB*	-0.038	1.161	0.104	322	1.162
BKK06140	*gutR*	-0.039	1.178	0.110	197	1.179
BKK10700	*gerPC*	-0.039	1.183	0.099	144	1.183
BKK13580	*mtnE*	-0.039	1.166	0.100	307	1.166
BKK29930	*amyX*	-0.044	1.156	0.096	114	1.156
BKK22010	*exoA*	-0.046	1.157	0.118	101	1.158
BKK17840	*fosB*	-0.048	1.163	0.101	79	1.164
BKK25420	*yqeW*	-0.049	1.137	0.093	161	1.138
BKK07610	*citM*	-0.052	1.208	0.162	70	1.208
BKK23970	*artQ*	-0.052	1.186	0.092	375	1.187
BKK29679	*ytzK*	-0.053	1.150	0.096	183	1.151
BKK06038	*ydzT*	-0.055	1.157	0.097	121	1.158
BKK10480	*yhjE*	-0.059	1.208	0.125	156	1.208
BKK39240	*yxxF*	-0.059	1.206	0.133	120	1.206
BKK02280	*ybfM*	-0.061	1.129	0.088	66	1.130
BKK08020	*yfjO*	-0.061	1.158	0.110	459	1.159
BKK21150	*yonB*	-0.067	1.154	0.116	258	1.155
BKK11020	*yitK*	-0.069	1.162	0.105	400	1.163
BKK36040	*ywrJ*	-0.072	1.160	0.105	395	1.161
BKK33160	*yvrA*	-0.076	1.133	0.087	67	1.134
BKK28280	*leuA*	-0.077	1.159	0.100	263	1.160
BKK22270	*yppE*	-0.081	1.099	0.127	308	1.100
BKK40420	*purA*	-0.082	1.147	0.103	301	1.147
BKK40530	*cotF*	-0.084	1.164	0.100	110	1.165
BKK23570	*ansB*	-0.086	1.186	0.099	322	1.187
BKK26110	*yqbH*	-0.088	1.176	0.101	82	1.177
BKK19230	*yocJ*	-0.09	1.161	0.098	389	1.162
BKK05190	*ydeG*	-0.094	1.200	0.122	600	1.201
BKK12790	*xhlA*	-0.096	1.165	0.126	299	1.166
BKK18300	*ppsE*	-0.096	1.137	0.098	126	1.138
BKK21280	*yomO*	-0.096	1.154	0.102	187	1.155
BKK18930	*yobH*	-0.097	1.161	0.108	456	1.162
BKK23240	*ypzK*	-0.097	1.152	0.111	266	1.153
BKK12900	*htrA*	-0.103	1.161	0.108	201	1.163
BKK33530	*yvaA*	-0.104	1.133	0.107	126	1.134
BKK00270	*yaaO*	-0.105	1.188	0.154	255	1.189
BKK30760	*mntB*	-0.107	1.155	0.104	87	1.156
BKK17510	*ynaC*	-0.112	1.162	0.108	108	1.164
BKK02040	*ybdN*	-0.113	1.158	0.112	519	1.160
BKK12990	*ykfC*	-0.113	1.207	0.136	89	1.208
BKK25060	*yqfZ*	-0.113	1.152	0.115	437	1.153
BKK00930	*cysE*	-0.114	1.099	0.119	344	1.100
BKK30830	*menF*	-0.114	1.155	0.097	132	1.156
BKK24690	*comGE*	-0.115	1.150	0.117	411	1.151
BKK28430	*sdhB*	-0.117	0.974	0.088	37	0.975
BKK11930	*yjcO*	-0.119	1.188	0.115	502	1.189
BKK33030	*yuxN*	-0.119	1.133	0.092	114	1.134
BKK16460	*cheD*	-0.12	1.160	0.105	594	1.161
BKK25100	*zur*	-0.121	1.162	0.099	222	1.164
BKK03670	*dtpT*	-0.122	1.137	0.098	191	1.138
BKK11200	*argJ*	-0.122	1.167	0.101	274	1.168
BKK06770	*yeeB*	-0.123	1.177	0.128	196	1.179
BKK24870	*glpG*	-0.124	1.162	0.106	221	1.164
BKK04150	*ycsN*	-0.126	1.156	0.110	186	1.158
BKK20360	*yorJ*	-0.126	1.138	0.109	54	1.139
BKK24090	*ptb*	-0.127	1.186	0.113	204	1.187
BKK03910	*gabD*	-0.129	1.137	0.094	125	1.138
BKK10550	*ntdA*	-0.13	1.167	0.107	302	1.168
BKK02900	*yceD*	-0.131	1.128	0.092	151	1.130
BKK04340	*ydaP*	-0.131	1.163	0.102	180	1.165
BKK19250	*yocL*	-0.131	1.170	0.087	154	1.172
BKK35699	*yvzE*	-0.132	1.160	0.104	471	1.161
BKK22650	*trpF*	-0.133	1.099	0.099	205	1.100
BKK23350	*ypzD*	-0.133	1.185	0.095	555	1.187
BKK02740	*natR*	-0.134	1.128	0.081	33	1.130
BKK13400	*ykoU*	-0.134	1.165	0.100	214	1.166
BKK21190	*yomX*	-0.135	1.150	0.103	417	1.151

BKK name [1]	gene	screening delta[2] (%)	average width (µm)	+/-	nb	AWP
BKK35680	*ggaB*	-0.137	1.160	0.101	475	1.161
BKK17500	*ynaB*	-0.138	1.160	0.102	456	1.162
BKK03500	*comS*	-0.14	1.156	0.117	101	1.158
BKK18200	*yngD*	-0.144	1.170	0.116	40	1.172
BKK08410	*yfiV*	-0.147	1.157	0.110	450	1.159
BKK25590	*comEA*	-0.147	1.162	0.108	120	1.164
BKK19600	*yodH*	-0.149	0.974	0.082	95	0.975
BKK22290	*sspM*	-0.157	1.099	0.129	223	1.100
BKK11530	*coiA*	-0.158	1.181	0.118	141	1.183
BKK11160	*yitW*	-0.163	1.181	0.102	106	1.183
BKK23800	*proI*	-0.163	1.152	0.110	315	1.153
BKK40800	*yyaN*	-0.163	1.183	0.110	194	1.184
BKK17660	*yncF*	-0.165	1.160	0.106	345	1.162
BKK39660	*yxdJ*	-0.165	1.163	0.121	126	1.165
BKK29750	*aroA*	-0.167	1.138	0.091	202	1.139
BKK16380	*flhB*	-0.168	1.151	0.108	434	1.153
BKK38900	*yxjM*	-0.169	1.206	0.165	87	1.208
BKK10240	*yhfI*	-0.177	1.175	0.097	124	1.177
BKK28290	*ilvC*	-0.179	1.158	0.093	161	1.160
BKK36790	*ywmA*	-0.179	1.155	0.107	122	1.157
BKK12560	*xpf*	-0.185	1.187	0.140	193	1.189
BKK33520	*csoR*	-0.19	1.132	0.095	118	1.134
BKK14820	*ylaL*	-0.191	1.204	0.122	65	1.206
BKK15000	*ylbG*	-0.192	1.177	0.122	43	1.179
BKK28160	*hemX*	-0.197	1.157	0.121	145	1.160
BKK39980	*qodI*	-0.198	1.145	0.110	477	1.147
BKK36340	*ywpE*	-0.202	1.155	0.133	66	1.157
BKK18570	*yoaE*	-0.206	1.136	0.092	106	1.138
BKK19610	*yodI*	-0.208	1.127	0.127	787	1.129
BKK02860	*adcC*	-0.209	1.145	0.105	129	1.147
BKK33640	*yvaM*	-0.209	1.132	0.083	92	1.134
BKK20480	*yoqX*	-0.21	1.159	0.112	334	1.162
BKK32859	*yuzM*	-0.211	1.132	0.098	229	1.134
BKK40070	*gntP*	-0.211	1.145	0.101	447	1.147
BKK05950	*ydiF*	-0.212	1.199	0.097	201	1.201
BKK10880	*yisV*	-0.212	1.181	0.120	113	1.183
BKK17810	*yndK*	-0.212	1.161	0.104	81	1.164
BKK12700	*xkdQ*	-0.214	1.137	0.122	51	1.139
BKK33550	*yvaC*	-0.219	1.155	0.093	106	1.158
BKK19880	*yotH*	-0.224	1.165	0.111	370	1.168
BKK22080	*ypwA*	-0.224	1.137	0.089	115	1.139
BKK10350	*yhfS*	-0.229	1.165	0.112	381	1.168
BKK17670	*cotU*	-0.229	1.161	0.102	60	1.164
BKK40360	*htrC*	-0.229	1.148	0.114	206	1.151
BKK08150	*yfjC*	-0.233	1.165	0.104	219	1.168
BKK27300	*yrrS*	-0.238	1.174	0.113	99	1.177
BKK11080	*yitQ*	-0.239	1.180	0.098	89	1.183
BKK22970	*mecB*	-0.239	1.098	0.119	380	1.100
BKK02140	*glpT*	-0.24	1.157	0.106	419	1.160
BKK07080	*yesZ*	-0.24	1.176	0.125	170	1.179
BKK11750	*cotY*	-0.24	1.160	0.105	250	1.163
BKK12070	*yjdJ*	-0.244	1.137	0.115	128	1.139
BKK33650	*yvzC*	-0.248	1.132	0.097	106	1.134
BKK06850	*yeeK*	-0.252	1.176	0.118	284	1.179
BKK33690	*yvaQ*	-0.252	1.131	0.089	144	1.134
BKK13730	*queD*	-0.253	1.165	0.113	294	1.168
BKK29580	*thiI*	-0.258	1.110	0.095	79	1.113
BKK10460	*yhjC*	-0.259	1.180	0.110	94	1.183
BKK17650	*yncE*	-0.259	1.161	0.121	77	1.164
BKK12950	*dppD*	-0.262	1.205	0.221	52	1.208
BKK25570	*comEC*	-0.262	1.148	0.113	195	1.151
BKK16960	*ymdA*	-0.267	1.160	0.118	66	1.164
BKK03430	*nucA*	-0.268	1.157	0.111	483	1.160
BKK14700	*nprE*	-0.272	1.176	0.130	125	1.179
BKK33080	*liaR*	-0.273	1.131	0.113	94	1.134
BKK33890	*yvbK*	-0.278	1.146	0.109	488	1.149
BKK15060	*ylbM*	-0.279	1.176	0.133	31	1.179
BKK08310	*yfiL*	-0.28	1.205	0.173	344	1.208
BKK29340	*tcyN*	-0.281	1.136	0.102	79	1.139
BKK03580	*yczE*	-0.282	1.135	0.083	161	1.138
BKK21090	*yonH*	-0.283	1.150	0.106	441	1.153
BKK01720	*ybbK*	-0.285	1.156	0.099	107	1.160
BKK09470	*yhdH*	-0.285	1.205	0.154	154	1.208
BKK08180	*malA*	-0.287	1.159	0.106	297	1.163
BKK11500	*spxA*	-0.288	1.154	0.108	160	1.158
BKK26400	*yrkS*	-0.289	1.150	0.104	160	1.153
BKK07290	*yfnF*	-0.294	0.972	0.101	55	0.975
BKK18950	*yozL*	-0.295	1.168	0.100	174	1.172
BKK41040	*oxaAA*	-0.295	1.163	0.156	108	1.166

BKK name [1]	gene	screening delta[2] (%)	average width (µm)	+/-	nb	AWP
BKK05110	*ydeA*	-0.297	1.156	0.100	417	1.160
BKK09320	*yhcY*	-0.299	1.135	0.095	61	1.138
BKK02690	*ansZ*	-0.3	1.198	0.073	70	1.201
BKK12930	*dppB*	-0.301	1.163	0.110	480	1.166
BKK11382	*appA*	-0.302	1.165	0.106	303	1.168
BKK30970	*glgC*	-0.303	1.153	0.122	89	1.156
BKK14410	*sipT*	-0.306	1.176	0.095	649	1.179
BKK30980	*glgB*	-0.307	1.152	0.086	152	1.156
BKK38130	*ywcE*	-0.307	1.153	0.109	126	1.157
BKK18360	*yoxA*	-0.308	1.168	0.118	122	1.172
BKK25500	*hemN*	-0.313	1.160	0.095	276	1.164
BKK26430	*yrkP*	-0.313	1.147	0.110	263	1.151
BKK08910	*yhbA*	-0.315	1.173	0.096	108	1.177
BKK20400	*yorF*	-0.318	1.158	0.109	391	1.162
BKK38110	*nfrA*	-0.321	1.144	0.114	420	1.147
BKK18280	*yngK*	-0.322	1.134	0.096	99	1.138
BKK08990	*yhbI*	-0.324	1.173	0.107	115	1.177
BKK01630	*feuA*	-0.329	1.198	0.064	38	1.201
BKK05690	*ydhB*	-0.329	1.163	0.080	303	1.167
BKK10520	*glcP*	-0.332	1.179	0.101	80	1.183
BKK31030	*yuaE*	-0.334	1.152	0.097	92	1.156
BKK02800	*ycdC*	-0.336	1.152	0.099	127	1.156
BKK39650	*yxdK*	-0.338	1.161	0.105	67	1.165
BKK12220	*yjiC*	-0.34	1.159	0.105	418	1.163
BKK39850	*yxbF*	-0.34	1.144	0.108	397	1.147
BKK32800	*yusH*	-0.341	1.154	0.096	112	1.158
BKK25000	*pbpA*	-0.343	0.972	0.075	76	0.975
BKK02540	*ycbK*	-0.344	1.197	0.064	120	1.201
BKK37340	*ywiB*	-0.344	1.162	0.113	66	1.166
BKK16470	*sigD*	-0.345	1.157	0.099	212	1.161
BKK39620	*yxeA*	-0.35	1.161	0.106	109	1.165
BKK08590	*yfhM*	-0.354	1.155	0.117	288	1.159
BKK17270	*ymaC*	-0.356	1.159	0.119	88	1.164
BKK19160	*yocC*	-0.356	1.153	0.102	125	1.158
BKK34050	*yvfV*	-0.359	1.130	0.093	74	1.134
BKK17850	*lexA*	-0.361	1.159	0.144	93	1.164
BKK28600	*yshB*	-0.362	1.155	0.094	108	1.160
BKK19220	*yocI*	-0.364	1.158	0.107	360	1.162
BKK10440	*yhjA*	-0.366	1.179	0.097	76	1.183
BKK28099	*yszA*	-0.366	1.155	0.096	293	1.160
BKK31420	*yugF*	-0.369	1.135	0.094	104	1.139
BKK26620	*yrdR*	-0.371	1.217	0.126	196	1.222
BKK39530	*yxeJ*	-0.374	1.160	0.101	76	1.165
BKK16799	*ylzJ*	-0.375	1.159	0.110	125	1.164
BKK40330	*rocE*	-0.38	1.147	0.095	426	1.151
BKK25490	*hrcA*	-0.381	1.159	0.103	336	1.164
BKK33870	*yvbI*	-0.386	1.157	0.112	359	1.161
BKK31160	*yubA*	-0.388	1.152	0.105	64	1.156
BKK31110	*yubF*	-0.389	1.152	0.092	86	1.156
BKK36670	*csbD*	-0.389	1.110	0.104	79	1.114
BKK29850	*ytoQ*	-0.39	1.157	0.109	367	1.161
BKK30810	*ytxM*	-0.393	1.151	0.113	71	1.156
BKK39190	*yxiG*	-0.394	1.143	0.104	454	1.147
BKK20730	*yopX*	-0.395	1.150	0.113	409	1.155
BKK05880	*gmuG*	-0.398	1.155	0.108	553	1.160
BKK08640	*yfhS*	-0.398	1.154	0.117	132	1.159
BKK22250	*yppG*	-0.4	1.096	0.138	56	1.100
BKK10260	*yhfK*	-0.405	1.172	0.093	116	1.177
BKK14330	*yknV*	-0.405	1.202	0.148	113	1.206
BKK29360	*tcyL*	-0.405	1.109	0.092	96	1.113
BKK29260	*ytpI*	-0.406	1.109	0.140	221	1.113
BKK26540	*yrkE*	-0.407	1.172	0.104	315	1.177
BKK07170	*yetI*	-0.408	1.174	0.123	200	1.179
BKK36240	*ywqE*	-0.41	1.157	0.119	478	1.161
BKK07260	*ltaSA*	-0.414	1.155	0.124	873	1.160
BKK00730	*cysK*	-0.415	1.150	0.157	186	1.155
BKK05060	*lrpB*	-0.415	1.155	0.117	452	1.160
BKK10380	*hemAT*	-0.416	1.203	0.179	117	1.208
BKK12440	*phrA*	-0.417	1.163	0.108	296	1.168
BKK38650	*yxlG*	-0.417	1.161	0.146	88	1.166
BKK05250	*ydeM*	-0.418	1.153	0.094	211	1.158
BKK02180	*ybfE*	-0.422	1.155	0.111	629	1.160
BKK22920	*ypeB*	-0.423	1.149	0.110	254	1.153
BKK06830	*rapH*	-0.426	1.174	0.112	301	1.179
BKK00230	*bofA*	-0.428	1.178	0.122	117	1.183
BKK02190	*ybfF*	-0.429	1.155	0.102	935	1.160
BKK06820	*yeeG*	-0.429	1.174	0.111	408	1.179
BKK34280	*epsJ*	-0.431	1.130	0.120	451	1.135
BKK40600	*yybL*	-0.431	1.161	0.112	83	1.166

Sup. Table 4: Cell width of mutants of the BKK collection (continued)

BKK name[1]	gene	screening delta[2] (%)	average width (µm)	+/-	nb	AWP
BKK31090	*ktrA*	-0.432	1.151	0.092	124	1.156
BKK23020	*recQ*	-0.434	1.096	0.111	512	1.100
BKK18840	*xynA*	-0.435	1.152	0.108	127	1.157
BKK36850	*atpF*	-0.436	1.161	0.107	114	1.166
BKK22730	*ndk*	-0.437	1.153	0.094	91	1.158
BKK02510	*garD*	-0.438	1.125	0.076	32	1.130
BKK27900	*pheA*	-0.445	1.154	0.110	78	1.160
BKK04010	*sipU*	-0.449	1.160	0.119	114	1.165
BKK29100	*phoR*	-0.449	1.108	0.104	53	1.113
BKK06460	*purS*	-0.45	1.174	0.111	333	1.179
BKK36810	*atpD*	-0.453	1.109	0.119	259	1.114
BKK06430	*purK*	-0.455	1.173	0.148	134	1.179
BKK07060	*yesX*	-0.456	1.152	0.108	93	1.158
BKK25810	*arsR*	-0.459	0.971	0.081	89	0.975
BKK02950	*yceI*	-0.46	1.196	0.139	96	1.201
BKK21870	*ilvD*	-0.46	1.150	0.108	532	1.155
BKK24050	*bkdAA*	-0.46	1.182	0.112	134	1.187
BKK38340	*ywbF*	-0.461	1.114	0.120	229	1.119
BKK29560	*ytcI*	-0.462	1.108	0.120	58	1.113
BKK04380	*ydaT*	-0.463	1.154	0.106	433	1.160
BKK14900	*ctaD*	-0.465	1.174	0.101	488	1.179
BKK06710	*swrC*	-0.466	1.154	0.107	502	1.160
BKK07140	*yetF*	-0.469	1.173	0.112	281	1.179
BKK21490	*sunI*	-0.469	1.148	0.110	595	1.153
BKK39590	*yxeD*	-0.47	1.159	0.108	161	1.165
BKK03359	*yczL*	-0.471	1.125	0.081	91	1.130
BKK19830	*yotM*	-0.474	1.124	0.141	604	1.129
BKK19640	*yodL*	-0.475	1.124	0.101	1106	1.129
BKK27150	*yrhK*	-0.479	1.148	0.110	477	1.153
BKK21690	*msrA*	-0.48	1.148	0.107	495	1.153
BKK24920	*yqgL*	-0.48	1.158	0.089	136	1.164
BKK28690	*glcF*	-0.484	1.146	0.109	285	1.151
BKK24840	*yqgS*	-0.485	1.201	0.157	48	1.206
BKK25090	*yqfW*	-0.489	1.158	0.099	203	1.164
BKK28960	*ytxC*	-0.491	1.108	0.096	33	1.113
BKK12910	*proG*	-0.492	1.161	0.094	429	1.166
BKK07160	*yetH*	-0.495	1.152	0.086	159	1.158
BKK31210	*yulB*	-0.497	1.150	0.089	111	1.156
BKK31240	*mcpA*	-0.497	1.150	0.091	103	1.156
BKK38730	*cydD*	-0.497	1.159	0.099	176	1.165
BKK21740	*ypmR*	-0.498	1.200	0.116	101	1.206
BKK33110	*liaG*	-0.498	1.129	0.109	107	1.134
BKK32849	*yuzL*	-0.499	1.129	0.089	135	1.134
BKK18630	*exlX*	-0.501	1.166	0.095	178	1.172
BKK13600	*mtnX*	-0.502	1.161	0.101	269	1.166
BKK33740	*yvaV*	-0.502	1.155	0.104	423	1.161
BKK39700	*iolG*	-0.502	1.159	0.125	128	1.165
BKK01680	*ybbF*	-0.509	1.154	0.108	425	1.160
BKK04039	*yczO*	-0.51	1.154	0.112	617	1.160
BKK13510	*ykzE*	-0.511	1.157	0.110	245	1.163
BKK19100	*yobV*	-0.512	1.166	0.081	90	1.172
BKK25150	*yqfQ*	-0.513	1.158	0.103	256	1.164
BKK40139	*yyzN*	-0.515	1.142	0.101	412	1.147
BKK23310	*sipS*	-0.516	1.181	0.112	172	1.187
BKK32930	*yusU*	-0.516	1.128	0.085	121	1.134
BKK33029	*yuzO*	-0.517	1.128	0.100	133	1.134
BKK12250	*yjkB*	-0.52	1.202	0.179	419	1.208
BKK20110	*yosI*	-0.52	1.123	0.159	139	1.129
BKK27000	*yraB*	-0.524	1.171	0.097	137	1.177
BKK04230	*ydaH*	-0.525	1.152	0.118	155	1.158
BKK07030	*yesU*	-0.527	1.173	0.126	429	1.179
BKK04160	*mtlR*	-0.532	1.159	0.110	91	1.165
BKK10180	*yhfC*	-0.533	1.156	0.105	283	1.163
BKK04650	*endB*	-0.537	1.159	0.096	107	1.165
BKK28610	*yshA*	-0.539	1.151	0.108	129	1.158
BKK28470	*lysC*	-0.54	0.970	0.073	32	0.975
BKK40640	*yybH*	-0.546	1.178	0.108	186	1.184
BKK07340	*yfnA*	-0.547	1.151	0.107	68	1.158
BKK02430	*glsA*	-0.548	1.153	0.096	386	1.160
BKK10830	*yisR*	-0.548	1.177	0.106	71	1.183
BKK08080	*acoC*	-0.555	1.152	0.100	334	1.159
BKK40100	*ahpF*	-0.556	1.162	0.106	417	1.168
BKK03360	*yciC*	-0.559	1.124	0.099	94	1.130
BKK36400	*flhO*	-0.559	1.155	0.090	293	1.161
BKK27700	*yrbF*	-0.56	1.215	0.088	197	1.222
BKK03240	*ycgQ*	-0.561	1.141	0.110	538	1.147
BKK17520	*ynaD*	-0.564	1.155	0.105	431	1.162
BKK19070	*yobS*	-0.564	1.165	0.082	212	1.172
BKK07270	*yfnH*	-0.565	1.151	0.104	109	1.158

BKK name[1]	gene	screening delta[2] (%)	average width (µm)	+/-	nb	AWP
BKK02300	*ybfN*	-0.569	1.153	0.110	417	1.160
BKK28660	*sspI*	-0.573	1.133	0.103	135	1.139
BKK25690	*sda*	-0.574	1.147	0.122	141	1.153
BKK11760	*cotX*	-0.575	1.182	0.129	720	1.189
BKK21900	*bsaA*	-0.575	1.148	0.114	230	1.155
BKK01890	*ybcL*	-0.578	1.131	0.119	141	1.138
BKK12930	*dppB*	-0.581	1.182	0.131	1017	1.189
BKK30920	*cotI*	-0.585	1.149	0.092	124	1.156
BKK33200	*yvrE*	-0.585	1.128	0.099	106	1.134
BKK06960	*yesN*	-0.586	1.172	0.105	370	1.179
BKK31250	*tlpA*	-0.588	1.149	0.104	117	1.156
BKK32590	*frlN*	-0.6	1.133	0.085	266	1.139
BKK40240	*yycS*	-0.602	1.144	0.110	363	1.151
BKK33510	*copZ*	-0.609	1.127	0.106	100	1.134
BKK27990	*minD*	-0.61	1.131	0.093	76	1.138
BKK23070	*serA*	-0.611	1.151	0.112	147	1.158
BKK03120	*ycgI*	-0.613	1.153	0.108	731	1.160
BKK29980	*ytjP*	-0.614	1.149	0.107	92	1.156
BKK01580	*ybaR*	-0.616	1.194	0.074	45	1.201
BKK04980	*yddI*	-0.618	1.152	0.105	375	1.160
BKK18030	*tlp*	-0.621	1.156	0.103	91	1.164
BKK33239	*yvrJ*	-0.622	1.127	0.105	103	1.134
BKK37240	*ywiE*	-0.625	1.112	0.110	74	1.119
BKK05200	*ydeH*	-0.628	1.160	0.112	169	1.167
BKK06034	*ydzT*	-0.628	1.160	0.122	635	1.167
BKK00990	*rpmGB*	-0.635	0.969	0.092	74	0.975
BKK31270	*tgl*	-0.635	1.149	0.096	54	1.156
BKK39920	*asnH*	-0.638	1.157	0.112	88	1.165
BKK39029	*yxiT*	-0.642	1.157	0.124	56	1.165
BKK17950	*yneJ*	-0.648	1.154	0.113	407	1.162
BKK30035	*ytzG*	-0.65	1.149	0.104	177	1.156
BKK22500	*ypjD*	-0.651	1.093	0.110	315	1.100
BKK07400	*yfmO*	-0.654	1.200	0.185	537	1.208
BKK00520	*ctc*	-0.655	1.156	0.119	116	1.164
BKK00720	*yacD*	-0.655	1.131	0.104	111	1.138
BKK22910	*ypfA*	-0.656	1.199	0.165	62	1.206
BKK03770	*rapC*	-0.658	1.131	0.084	139	1.138
BKK11070	*yitP*	-0.658	1.176	0.130	112	1.183
BKK22190	*ypsA*	-0.658	1.093	0.115	234	1.100
BKK25480	*grpE*	-0.658	1.156	0.094	228	1.164
BKK19590	*ctpA*	-0.659	1.122	0.140	308	1.129
BKK13430	*ykoX*	-0.661	1.200	0.116	84	1.208
BKK30180	*ytbQ*	-0.663	1.148	0.087	125	1.156
BKK07560	*pel*	-0.667	1.151	0.108	301	1.159
BKK09850	*yhaU*	-0.671	1.200	0.142	65	1.208
BKK32830	*fadA*	-0.673	1.127	0.096	160	1.134
BKK09440	*citA*	-0.68	1.155	0.099	349	1.163
BKK07810	*treA*	-0.683	1.151	0.119	341	1.159
BKK29240	*ytrI*	-0.684	1.105	0.107	208	1.113
BKK12640	*xkdJ*	-0.686	1.160	0.117	308	1.168
BKK38520	*dltC*	-0.686	1.142	0.117	106	1.149
BKK21410	*blyA*	-0.688	1.145	0.108	318	1.153
BKK15860	*sdaAA*	-0.69	1.107	0.119	86	1.114
BKK30110	*yteS*	-0.691	1.130	0.098	172	1.138
BKK35560	*tuaF*	-0.692	0.968	0.082	46	0.975
BKK24760	*rsbRD*	-0.694	1.155	0.095	103	1.164
BKK30420	*ytrE*	-0.694	1.214	0.099	160	1.222
BKK26640	*yrdP*	-0.697	1.145	0.098	461	1.153
BKK31820	*yuzF*	-0.697	1.132	0.096	98	1.139
BKK23580	*ansA*	-0.698	1.179	0.099	267	1.187
BKK38100	*ywcH*	-0.7	1.149	0.098	103	1.157
BKK12870	*mhqA*	-0.703	1.158	0.122	71	1.166
BKK36220	*ywqG*	-0.704	1.149	0.105	146	1.157
BKK36650	*ureB*	-0.704	1.149	0.105	146	1.157
BKK24460	*yqhT*	-0.705	1.143	0.104	402	1.151
BKK32920	*yusT*	-0.705	0.968	0.072	110	0.975
BKK40930	*yyaE*	-0.705	1.148	0.106	143	1.156
BKK36740	*ywmC*	-0.708	1.106	0.103	101	1.114
BKK13460	*rsgI*	-0.71	1.160	0.110	284	1.168
BKK15300	*bpr*	-0.712	1.154	0.110	315	1.163
BKK24440	*yqhV*	-0.713	1.179	0.103	289	1.187
BKK17266	*ymzE*	-0.714	1.155	0.085	76	1.164
BKK40880	*exoAA*	-0.716	1.176	0.089	156	1.184
BKK16700	*ylxY*	-0.721	1.155	0.112	89	1.164
BKK37220	*ywjB*	-0.724	1.158	0.116	106	1.166
BKK26840	*sigZ*	-0.727	1.168	0.101	270	1.177
BKK15480	*pyrP*	-0.732	1.148	0.110	96	1.156
BKK06040	*ydiM*	-0.733	1.131	0.120	118	1.139
BKK23630	*yqkE*	-0.733	1.178	0.094	656	1.187

BKK name[1]	gene	screening delta[2] (%)	average width (µm)	+/-	nb	AWP
BKK25830	*rapE*	-0.734	1.155	0.093	64	1.164
BKK17700	*cotC*	-0.739	1.155	0.094	106	1.164
BKK17740	*ynzB*	-0.739	1.153	0.107	311	1.162
BKK21820	*thyB*	-0.743	1.143	0.106	188	1.151
BKK09958	*yhzE*	-0.744	1.168	0.092	134	1.177
BKK35160	*uvrA*	-0.745	1.127	0.119	693	1.135
BKK24810	*yqgV*	-0.747	1.155	0.092	280	1.164
BKK17160	*pksH*	-0.756	1.155	0.097	112	1.164
BKK29740	*ccpA*	-0.758	1.153	0.142	154	1.161
BKK17240	*ymzB*	-0.759	1.153	0.108	396	1.162
BKK32890	*yusQ*	-0.766	1.126	0.098	134	1.134
BKK01810	*adaA*	-0.767	1.151	0.096	148	1.160
BKK19750	*cgeE*	-0.771	1.121	0.138	159	1.129
BKK04500	*ydbK*	-0.773	1.151	0.117	466	1.160
BKK19479	*yoyC*	-0.781	1.120	0.107	532	1.129
BKK21200	*yomW*	-0.781	1.146	0.100	543	1.155
BKK14340	*yknW*	-0.782	1.170	0.122	261	1.179
BKK12150	*yjgB*	-0.783	1.180	0.151	191	1.189
BKK31140	*cdoA*	-0.788	1.147	0.111	87	1.156
BKK11620	*yjbO*	-0.789	1.130	0.105	59	1.139
BKK22960	*gudB*	-0.791	1.148	0.108	82	1.158
BKK24300	*xseA*	-0.791	1.178	0.104	258	1.187
BKK27740	*ruvA*	-0.791	1.150	0.119	126	1.160
BKK05899	*ydhU*	-0.792	1.130	0.098	190	1.139
BKK36450	*ywoG*	-0.792	1.140	0.085	265	1.149
BKK22019	*ypzF*	-0.794	1.092	0.147	61	1.100
BKK31860	*yueB*	-0.795	1.139	0.110	505	1.148
BKK18990	*yobK*	-0.797	1.129	0.089	101	1.138
BKK17930	*ccdA*	-0.799	1.154	0.133	71	1.164
BKK25440	*rsmE*	-0.801	1.154	0.099	258	1.164
BKK33230	*sigO*	-0.801	0.967	0.074	94	0.975
BKK02130	*glpQ*	-0.805	1.150	0.111	551	1.160
BKK19580	*yodF*	-0.807	1.120	0.106	819	1.129
BKK29370	*tcyK*	-0.807	1.129	0.094	64	1.138
BKK12720	*xkdS*	-0.811	1.130	0.101	226	1.139
BKK30070	*opuD*	-0.812	1.152	0.107	355	1.161
BKK31080	*yuaB*	-0.818	1.147	0.102	80	1.156
BKK28100	*ysxE*	-0.825	1.150	0.101	367	1.160
BKK01020	*rplK*	-0.826	1.154	0.099	453	1.164
BKK25890	*yqxI*	-0.828	1.144	0.104	437	1.153
BKK14760	*ylaF*	-0.829	1.169	0.131	403	1.179
BKK18440	*gltB*	-0.829	1.158	0.108	258	1.168
BKK37760	*rocC*	-0.83	1.109	0.140	54	1.119
BKK38070	*sacT*	-0.834	1.109	0.169	30	1.119
BKK05410	*ydfH*	-0.835	1.138	0.102	406	1.147
BKK38870	*yxkA*	-0.836	1.138	0.105	378	1.147
BKK11980	*yjdA*	-0.837	1.153	0.111	259	1.163
BKK02110	*ybyB*	-0.843	1.120	0.107	72	1.130
BKK08890	*ygaO*	-0.847	1.158	0.106	277	1.168
BKK34290	*epsI*	-0.849	1.156	0.109	145	1.166
BKK38080	*ywcI*	-0.858	1.109	0.093	102	1.119
BKK26930	*yraI*	-0.859	1.167	0.112	295	1.177
BKK07820	*treR*	-0.861	1.149	0.112	507	1.159
BKK23100	*sigX*	-0.863	1.091	0.118	364	1.100
BKK24780	*yqgY*	-0.863	1.154	0.104	231	1.164
BKK00260	*yaaN*	-0.864	1.156	0.136	719	1.166
BKK16360	*fliQ*	-0.865	1.152	0.118	488	1.162
BKK03652	*yclD*	-0.867	1.128	0.098	144	1.138
BKK19420	*yojK*	-0.867	1.152	0.106	434	1.162
BKK23910	*yqjE*	-0.867	1.141	0.127	264	1.151
BKK32410	*pucH*	-0.867	1.130	0.085	208	1.140
BKK32950	*yusW*	-0.869	1.128	0.090	114	1.138
BKK10160	*yhgE*	-0.875	1.167	0.076	97	1.177
BKK10250	*yhfJ*	-0.875	1.167	0.089	101	1.177
BKK03789	*yczN*	-0.88	1.155	0.104	309	1.165
BKK20470	*yoqY*	-0.88	1.152	0.113	368	1.162
BKK18870	*yozI*	-0.883	1.152	0.114	418	1.162
BKK31200	*yulC*	-0.889	1.147	0.102	142	1.158
BKK33260	*yvrN*	-0.889	1.147	0.119	95	1.158
BKK01640	*ybbB*	-0.89	1.149	0.118	376	1.160
BKK05460	*ydfL*	-0.892	1.149	0.105	224	1.160
BKK03400	*yckD*	-0.893	0.966	0.075	74	0.975
BKK14060	*fadH*	-0.893	1.156	0.119	643	1.166
BKK04090	*kipA*	-0.897	1.154	0.086	123	1.165
BKK31470	*kapD*	-0.899	1.138	0.118	264	1.148
BKK06812	*yeeF*	-0.909	1.149	0.105	430	1.160
BKK27450	*glnM*	-0.91	1.149	0.094	177	1.160
BKK21260	*yomQ*	-0.911	1.141	0.115	260	1.151
BKK34650	*yvdC*	-0.911	1.125	0.094	30	1.135

BKK name[1]	gene	screening delta[2] (%)	average width (µm)	+/-	nb	AWP
BKK40340	*rocD*	-0.911	1.141	0.112	191	1.151
BKK35698	*yvzI*	-0.914	1.104	0.094	225	1.114
BKK35790	*yvyI*	-0.914	1.104	0.120	98	1.114
BKK39860	*aldX*	-0.918	1.154	0.091	94	1.165
BKK03030	*ycgB*	-0.919	1.120	0.100	159	1.130
BKK16300	*fliL*	-0.921	1.157	0.104	169	1.168
BKK19740	*yodT*	-0.924	1.119	0.109	569	1.129
BKK24010	*bmr*	-0.924	1.147	0.097		1.158
BKK10750	*yisK*	-0.926	1.152	0.113	418	1.163
BKK34720	*yvcP*	-0.928	1.139	0.095	317	1.149
BKK27570	*yrzK*	-0.931	1.149	0.089	55	1.160
BKK35200	*yvkB*	-0.934	1.124	0.109	345	1.135
BKK12470	*yjqA*	-0.935	1.178	0.098	823	1.189
BKK15070	*ylbN*	-0.935	1.152	0.105	238	1.163
BKK18040	*yneP*	-0.935	1.157	0.116	222	1.168
BKK07020	*rhgT*	-0.943	1.168	0.114	406	1.179
BKK03390	*yckC*	-0.946	1.147	0.111	106	1.158
BKK03960	*ycnK*	-0.946	1.149	0.105	386	1.160
BKK29380	*tcyJ*	-0.946	1.127	0.085	113	1.138
BKK04200	*ydaE*	-0.947	1.154	0.109	57	1.165
BKK25350	*yqfD*	-0.948	1.153	0.103	226	1.164
BKK23328	*ypzJ*	-0.949	1.140	0.105	342	1.151
BKK40270	*yycP*	-0.951	1.140	0.100	356	1.151
BKK25990	*yqbS*	-0.953	1.140	0.115	245	1.151
BKK06750	*yefC*	-0.956	1.168	0.108	131	1.179
BKK12210	*yjiB*	-0.956	1.178	0.101	1308	1.189
BKK11850	*yjcG*	-0.958	1.178	0.110	408	1.189
BKK19700	*yodP*	-0.961	1.195	0.135	101	1.206
BKK20750	*yopV*	-0.962	1.144	0.097	498	1.155
BKK40190	*fbp*	-0.966	1.157	0.108	254	1.168
BKK20970	*yonX*	-0.97	1.142	0.100	490	1.153
BKK01690	*ybbH*	-0.973	1.165	0.106	289	1.177
BKK32180	*yutK*	-0.978	1.210	0.067	54	1.222
BKK02420	*glnT*	-0.98	1.148	0.097	479	1.160
BKK24630	*sipW*	-0.981	0.965	0.084	50	0.975
BKK25080	*yqfX*	-0.982	1.152	0.114	249	1.164
BKK17790	*yndH*	-0.991	1.156	0.108	352	1.168
BKK19780	*cgeA*	-0.992	1.146	0.110	134	1.158
BKK09660	*yheN*	-0.993	1.165	0.104	115	1.177
BKK17620	*yncB*	-0.994	1.150	0.110	420	1.162
BKK13470	*sspD*	-0.995	1.155	0.112	115	1.166
BKK09710	*yheI*	-1	1.196	0.114	55	1.208
BKK26670	*gltR*	-1.003	1.142	0.099	508	1.153
BKK37650	*cysL*	-1.004	1.108	0.109	257	1.119
BKK13650	*eag*	-1.005	1.151	0.108	313	1.163
BKK24670	*comGG*	-1.009	1.152	0.098	163	1.164
BKK24860	*yqgQ*	-1.012	1.152	0.100	316	1.164
BKK17870	*yneB*	-1.014	1.150	0.113	307	1.162
BKK25120	*yqfT*	-1.014	1.142	0.104	482	1.153
BKK14150	*ykuN*	-1.018	1.167	0.123	907	1.179
BKK36950	*ywlC*	-1.02	0.965	0.112	41	0.975
BKK23110	*resE*	-1.029	1.194	0.094	126	1.206
BKK26830	*yrpE*	-1.029	1.209	0.067	94	1.222
BKK26900	*yraL*	-1.032	1.165	0.102	431	1.177
BKK34220	*epsO*	-1.033	1.123	0.111	96	1.135
BKK40010	*yxaD*	-1.036	1.136	0.107	433	1.147
BKK35880	*pgsA*	-1.037	1.138	0.095	548	1.149
BKK36669	*ywzF*	-1.038	1.136	0.100	159	1.147
BKK27190	*yrzI*	-1.039	1.146	0.112	86	1.158
BKK02530	*rtpA*	-1.04	1.136	0.101	428	1.147
BKK34690	*yvcS*	-1.041	1.137	0.105	563	1.149
BKK33780	*sdpI*	-1.043	1.122	0.096	107	1.134
BKK06780	*yeeC*	-1.045	1.167	0.102	601	1.179
BKK37610	*ywzC*	-1.045	1.107	0.104	320	1.119
BKK38540	*dltE*	-1.046	0.965	0.084	50	0.975
BKK33060	*gerAB*	-1.047	1.122	0.095	77	1.134
BKK33300	*fhuG*	-1.047	1.122	0.103	99	1.134
BKK34710	*yvcQ*	-1.047	1.137	0.106	440	1.149
BKK03230	*ycgP*	-1.048	1.118	0.091	41	1.130
BKK26880	*yraM*	-1.051	1.141	0.102	504	1.153
BKK26420	*yrkQ*	-1.054	1.209	0.092	161	1.222
BKK35800	*gerBA*	-1.054	1.103	0.087	38	1.114
BKK23000	*ypbE*	-1.057	1.089	0.113	607	1.100
BKK18750	*yooT*	-1.064	1.159	0.106	218	1.172
BKK18900	*yozJ*	-1.064	1.126	0.093	69	1.138
BKK31590	*yufS*	-1.064	1.127	0.110	61	1.139
BKK05120	*cspC*	-1.067	1.147	0.120	380	1.160
BKK10910	*yisZ*	-1.069	1.171	0.097	92	1.183
BKK12820	*spoIISB*	-1.069	1.195	0.160	87	1.208

Sup. Table 4: Cell width of mutants of the BKK collection (continued)

BKK name [1]	gene	screening delta^2 (%)	average width (µm)	+/-	nb	AWP
BKK33120	*liaH*	-1.071	1.122	0.084	63	1.134
BKK05260	*ydeN*	-1.075	1.155	0.097	89	1.167
BKK24160	*mmgB*	-1.075	1.141	0.105	257	1.153
BKK33320	*fhuD*	-1.075	1.209	0.105	252	1.222
BKK01710	*ybbJ*	-1.077	1.164	0.102	133	1.177
BKK27470	*yrrD*	-1.077	1.147	0.099	194	1.160
BKK18350	*dacC*	-1.078	1.193	0.135	91	1.206
BKK21550	*yokL*	-1.079	1.142	0.104	393	1.155
BKK39230	*wapA*	-1.084	1.193	0.143	195	1.206
BKK19870	*yotI*	-1.086	1.117	0.135	332	1.129
BKK09770	*yheD*	-1.091	1.155	0.097	330	1.168
BKK21020	*yonR*	-1.092	1.141	0.099	548	1.153
BKK36420	*spoIIID*	-1.094	1.137	0.107	357	1.149
BKK00250	*xpaC*	-1.095	1.154	0.113	166	1.166
BKK17300	*ebrA*	-1.095	1.127	0.080	151	1.139
BKK28970	*ytxB*	-1.096	1.149	0.102	394	1.161
BKK18960	*yozM*	-1.103	1.149	0.111	410	1.162
BKK40150	*yydI*	-1.104	1.135	0.095	299	1.147
BKK21320	*yomL*	-1.106	1.141	0.119	521	1.153
BKK23860	*gndA*	-1.107	1.174	0.123	247	1.187
BKK15760	*prpC*	-1.108	1.122	0.105	108	1.134
BKK02330	*ybfQ*	-1.111	1.117	0.101	90	1.130
BKK19300	*yozC*	-1.114	1.149	0.115	374	1.162
BKK38960	*yxjG*	-1.116	1.152	0.112	148	1.165
BKK07180	*yetI*	-1.117	1.166	0.110	343	1.179
BKK03090	*ycgF*	-1.125	1.147	0.117	276	1.160
BKK08600	*csbB*	-1.128	1.149	0.111	343	1.163
BKK22260	*yppF*	-1.128	1.127	0.099	97	1.139
BKK05440	*nap*	-1.129	1.145	0.093	126	1.158
BKK31680	*comA*	-1.13	1.127	0.091	114	1.139
BKK34200	*sigL*	-1.131	1.122	0.150	50	1.135
BKK07890	*yfkI*	-1.132	1.146	0.112	300	1.159
BKK10680	*gerPE*	-1.132	1.155	0.094	302	1.168
BKK34850	*yvcA*	-1.135	1.136	0.117	519	1.149
BKK06280	*ydjP*	-1.136	1.134	0.107	302	1.147
BKK10420	*comK*	-1.136	1.170	0.094	96	1.183
BKK11230	*carA*	-1.136	1.170	0.126	87	1.183
BKK32530	*pucF*	-1.138	1.135	0.141	51	1.148
BKK27680	*yrbG*	-1.143	1.146	0.099	381	1.160
BKK13170	*guaD*	-1.145	1.153	0.132	208	1.166
BKK06390	*yebD*	-1.146	1.146	0.108	324	1.160
BKK30200	*bioB*	-1.148	1.143	0.097	92	1.156
BKK30300	*melA*	-1.149	1.143	0.096	118	1.156
BKK35060	*cypX*	-1.149	1.122	0.122	509	1.135
BKK17820	*yndL*	-1.152	1.150	0.117	119	1.164
BKK04050	*ycsF*	-1.156	1.134	0.098	508	1.147
BKK13870	*ykvZ*	-1.157	1.149	0.104	324	1.163
BKK39460	*yxeQ*	-1.157	1.151	0.090	123	1.165
BKK38820	*yxkF*	-1.158	1.151	0.100	179	1.165
BKK31450	*kinB*	-1.16	1.208	0.062	59	1.222
BKK27010	*adhA*	-1.166	1.163	0.099	387	1.177
BKK20980	*yonV*	-1.167	1.140	0.101	590	1.153
BKK21660	*yokA*	-1.168	1.141	0.094	236	1.155
BKK18190	*yngC*	-1.169	1.192	0.143	65	1.206
BKK05080	*yddR*	-1.171	1.154	0.114	251	1.167
BKK25730	*yqeC*	-1.173	1.150	0.116	153	1.164
BKK00120	*pdxT*	-1.176	1.144	0.111	91	1.158
BKK22850	*seaA*	-1.178	1.087	0.116	159	1.100
BKK00430	*yabG*	-1.179	1.150	0.094	141	1.164
BKK13299	*ykzO*	-1.179	1.087	0.112	133	1.100
BKK32400	*yunG*	-1.179	1.134	0.128	144	1.148
BKK09530	*yhdN*	-1.18	1.149	0.118	291	1.163
BKK29970	*ytkP*	-1.181	1.142	0.114	107	1.156
BKK17350	*ymzC*	-1.186	1.148	0.109	446	1.162
BKK10170	*fabHB*	-1.187	1.163	0.097	106	1.177
BKK32030	*bioYB*	-1.187	1.208	0.068	97	1.222
BKK37310	*fnr*	-1.187	1.134	0.101	308	1.147
BKK37580	*ywgB*	-1.188	1.105	0.120	802	1.119
BKK12649	*ykzM*	-1.189	1.154	0.111	241	1.168
BKK02910	*yceE*	-1.191	1.117	0.099	166	1.130
BKK13670	*mhqR*	-1.191	1.152	0.116	231	1.166
BKK15660	*yloC*	-1.191	1.121	0.102	102	1.134
BKK08520	*recX*	-1.193	1.145	0.103	280	1.159
BKK33090	*liaS*	-1.193	1.207	0.126	67	1.222
BKK12180	*yjhA*	-1.199	1.194	0.161	146	1.208
BKK16160	*clpY*	-1.2	1.149	0.105	311	1.163
BKK04820	*immR*	-1.201	1.144	0.095	123	1.158
BKK30090	*yteU*	-1.218	1.124	0.099	116	1.138
BKK38500	*dltA*	-1.218	1.135	0.124	115	1.149
BKK08380	*yfiS*	-1.219	1.145	0.110	285	1.159
BKK35920	*rbsK*	-1.219	1.101	0.093	72	1.114
BKK08320	*yfiM*	-1.221	1.145	0.107	270	1.159
BKK30380	*bceA*	-1.222	1.207	0.080	97	1.222
BKK24650	*yqzG*	-1.223	1.173	0.107	165	1.187
BKK20850	*yopL*	-1.224	1.137	0.101	257	1.151
BKK36570	*ywnG*	-1.224	1.101	0.107	208	1.114
BKK20130	*yosG*	-1.225	1.148	0.109	236	1.162
BKK26780	*yrdA*	-1.225	1.139	0.111	450	1.153
BKK04630	*ydcC*	-1.228	1.187	0.115	558	1.201
BKK20740	*yopW*	-1.228	1.141	0.097	412	1.155
BKK38840	*yxkD*	-1.233	1.193	0.123	83	1.208
BKK38580	*licC*	-1.234	1.152	0.127	75	1.166
BKK14170	*ykuP*	-1.241	1.148	0.098	319	1.163
BKK07230	*yetM*	-1.242	1.164	0.111	529	1.179
BKK09090	*yhcI*	-1.244	1.154	0.112	287	1.168
BKK38880	*yxjO*	-1.244	1.150	0.102	79	1.165
BKK36700	*moaA*	-1.245	1.100	0.111	86	1.114
BKK06940	*yesL*	-1.246	1.153	0.099	342	1.168
BKK07500	*yfmE*	-1.247	1.144	0.102	257	1.159
BKK38710	*yxlA*	-1.25	1.150	0.114	148	1.165
BKK03330	*nasA*	-1.255	1.186	0.074	69	1.201
BKK02870	*adcB*	-1.26	1.124	0.109	108	1.138
BKK39420	*deoC*	-1.262	1.150	0.108	126	1.165
BKK35910	*rbsR*	-1.264	1.152	0.108	135	1.166
BKK20890	*yopH*	-1.265	1.139	0.091	514	1.153
BKK26680	*yrdK*	-1.269	1.136	0.100	279	1.151
BKK17450	*glnR*	-1.272	1.147	0.101	632	1.162
BKK11440	*oppB*	-1.273	1.193	0.162	80	1.208
BKK20070	*nrdIB*	-1.276	1.115	0.120	112	1.129
BKK00400	*yabE*	-1.28	1.125	0.106	65	1.139
BKK37130	*spo0F*	-1.28	1.135	0.095	525	1.149
BKK17130	*acpK*	-1.293	1.148	0.120	90	1.164
BKK34610	*mdxE*	-1.296	1.146	0.117	352	1.161
BKK25390	*yqeZ*	-1.297	1.148	0.095	182	1.164
BKK35280	*yvjA*	-1.298	1.120	0.111	324	1.135
BKK34040	*yvfW*	-1.299	1.120	0.090	138	1.134
BKK11000	*yitI*	-1.302	1.147	0.117	303	1.163
BKK21880	*ypgR*	-1.302	1.140	0.097	477	1.155
BKK00370	*abrB*	-1.303	1.164	0.146	91	1.179
BKK06450	*purC*	-1.303	1.163	0.119	259	1.179
BKK20280	*yorR*	-1.303	1.153	0.106	244	1.168
BKK38230	*ywcB*	-1.303	1.104	0.117	69	1.119
BKK17720	*yndA*	-1.304	1.147	0.106	409	1.162
BKK12730	*xkdT*	-1.307	1.173	0.126	110	1.189
BKK26390	*spoIIIC*	-1.313	1.161	0.109	187	1.177
BKK31400	*yugH*	-1.315	1.133	0.116	120	1.148
BKK12550	*xtrA*	-1.317	1.153	0.120	208	1.168
BKK34660	*yvdB*	-1.318	1.134	0.106	357	1.149
BKK06760	*yeeA*	-1.321	1.163	0.105	420	1.179
BKK07510	*yfmD*	-1.321	1.192	0.147	53	1.208
BKK20860	*yopK*	-1.323	1.138	0.112	512	1.153
BKK03860	*ycnD*	-1.324	1.132	0.103	386	1.147
BKK05660	*ydgI*	-1.325	1.142	0.106	80	1.158
BKK23430	*spoVAB*	-1.33	1.190	0.130	100	1.206
BKK07990	*yfjR*	-1.331	1.143	0.109	324	1.159
BKK04210	*ydaF*	-1.333	1.149	0.096	136	1.165
BKK37810	*spsL*	-1.339	1.134	0.110	405	1.149
BKK06920	*yesJ*	-1.342	1.163	0.127	285	1.179
BKK30410	*ytrF*	-1.343	1.140	0.101	163	1.156
BKK39510	*yxeL*	-1.347	1.149	0.106	82	1.165
BKK04780	*ydcI*	-1.352	1.149	0.133	181	1.165
BKK01980	*ybbA*	-1.353	1.144	0.101	436	1.160
BKK08360	*yfiQ*	-1.354	1.143	0.120	230	1.159
BKK02620	*phoD*	-1.357	1.132	0.109	508	1.147
BKK37380	*albB*	-1.359	1.104	0.094	61	1.119
BKK24200	*yqiH*	-1.361	1.190	0.163	31	1.206
BKK38790	*yxzE*	-1.361	1.149	0.108	112	1.165
BKK18800	*penP*	-1.364	0.962	0.101	39	0.975
BKK27690	*yrzE*	-1.364	1.144	0.103	394	1.160
BKK31930	*ald*	-1.364	1.132	0.120	51	1.148
BKK10960	*yitE*	-1.373	1.167	0.103	143	1.183
BKK13100	*ykkD*	-1.373	1.192	0.138	130	1.208
BKK25430	*rimO*	-1.374	1.148	0.103	365	1.164
BKK34920	*hisG*	-1.377	1.119	0.113	100	1.135
BKK35430	*flgM*	-1.377	1.141	0.106	89	1.157
BKK16480	*ylxL*	-1.38	1.145	0.117	375	1.161
BKK11381	*appA*	-1.386	1.167	0.107	111	1.183
BKK10760	*yisL*	-1.387	1.167	0.115	123	1.183
BKK29760	*ytxJ*	-1.39	1.124	0.095	87	1.139
BKK18520	*yoxB*	-1.391	1.141	0.105	122	1.157
BKK34520	*yvdP*	-1.391	1.119	0.104	77	1.135
BKK25740	*yqeB*	-1.396	1.147	0.112	122	1.164
BKK28940	*ysaA*	-1.398	1.098	0.120	292	1.113
BKK40480	*yycB*	-1.399	1.131	0.099	412	1.147
BKK21390	*yomE*	-1.401	1.137	0.109	434	1.153
BKK39120	*yxiM*	-1.404	1.148	0.116	30	1.165
BKK12460	*xlyB*	-1.41	1.172	0.109	914	1.189
BKK33380	*yvgL*	-1.41	1.205	0.089	94	1.222
BKK06310	*gabP*	-1.411	1.143	0.104	835	1.160
BKK19920	*yotD*	-1.414	1.113	0.142	144	1.129
BKK03982	*mtlF*	-1.419	1.148	0.084	173	1.165
BKK17730	*yndB*	-1.42	1.145	0.105	310	1.162
BKK21540	*yolA*	-1.423	1.138	0.114	236	1.155
BKK36640	*ureC*	-1.424	1.140	0.111	147	1.157
BKK37190	*clsB*	-1.424	1.103	0.104	64	1.119
BKK06250	*ydjM*	-1.425	1.162	0.107	316	1.179
BKK30770	*mntA*	-1.426	1.145	0.105	389	1.161
BKK18540	*yoaB*	-1.428	1.122	0.093	174	1.138
BKK33240	*oxdC*	-1.428	1.118	0.106	149	1.134
BKK07940	*yfkC*	-1.432	1.142	0.106	337	1.159
BKK37060	*tdk*	-1.432	1.098	0.123	30	1.114
BKK30080	*yteV*	-1.434	1.139	0.106	98	1.156
BKK11130	*ipi*	-1.437	1.166	0.095	131	1.183
BKK23820	*yqjM*	-1.437	1.137	0.105	489	1.153
BKK15770	*prkC*	-1.438	1.151	0.101	374	1.168
BKK39339	*yxzL*	-1.441	1.131	0.105	357	1.147
BKK29680	*acsA*	-1.445	1.097	0.115	54	1.113
BKK16430	*cheA*	-1.446	1.122	0.117	107	1.138
BKK27469	*yrzR*	-1.447	1.134	0.101	274	1.151
BKK06990	*yesQ*	-1.452	1.191	0.193	175	1.208
BKK35140	*yvkN*	-1.453	1.119	0.112	363	1.135
BKK36880	*atpI*	-1.454	1.098	0.135	177	1.114
BKK02570	*ycbN*	-1.461	1.184	0.071	153	1.201
BKK08390	*yfiT*	-1.468	1.142	0.113	277	1.159
BKK07090	*yetA*	-1.471	1.162	0.122	231	1.179
BKK22590	*ypiA*	-1.472	1.084	0.101	235	1.100
BKK05090	*yddS*	-1.475	1.143	0.116	439	1.160
BKK25230	*yqxD*	-1.475	1.146	0.092	192	1.164
BKK38920	*pepT*	-1.475	1.148	0.125	149	1.165
BKK18580	*yoaF*	-1.478	1.121	0.092	73	1.138
BKK11510	*yjbE*	-1.479	1.166	0.107	141	1.183
BKK00240	*csfB*	-1.48	1.166	0.114	178	1.183
BKK02840	*ycdG*	-1.48	1.113	0.095	53	1.130
BKK17610	*xylB*	-1.481	1.145	0.110	292	1.162
BKK10270	*lcfB*	-1.482	1.166	0.086	74	1.183
BKK16110	*dprA*	-1.482	1.142	0.122	183	1.160
BKK08790	*thiC*	-1.487	1.142	0.103	187	1.159
BKK14430	*ykpA*	-1.487	1.145	0.098	374	1.163
BKK03710	*gerKC*	-1.488	1.121	0.090	59	1.138
BKK08480	*yfhC*	-1.488	1.142	0.120	318	1.159
BKK36100	*ywrD*	-1.488	1.140	0.095	90	1.157
BKK13630	*ykvA*	-1.49	0.960	0.069	99	0.975
BKK14680	*ykzC*	-1.493	1.162	0.162	93	1.179
BKK28790	*araB*	-1.493	1.122	0.098	118	1.139
BKK32070	*yuiC*	-1.495	1.131	0.082	182	1.148
BKK16310	*fliM*	-1.501	1.145	0.113	212	1.163
BKK08510	*yfhF*	-1.503	1.141	0.104	306	1.159
BKK16060	*rnhB*	-1.503	1.147	0.096	66	1.165
BKK19800	*phy*	-1.503	1.144	0.115	426	1.162
BKK35770	*tagC*	-1.504	1.144	0.114	343	1.161
BKK18020	*sspN*	-1.506	1.146	0.113	69	1.164
BKK32520	*pucG*	-1.513	1.204	0.064	126	1.222
BKK17190	*pksL*	-1.514	1.150	0.101	460	1.168
BKK23060	*ypzE*	-1.514	1.140	0.088	95	1.158
BKK11139	*yizC*	-1.517	1.145	0.105	410	1.163
BKK26950	*yraG*	-1.518	1.159	0.109	169	1.177
BKK18120	*alsT*	-1.521	1.154	0.123	126	1.172
BKK24380	*spoIIIAF*	-1.521	1.188	0.140	54	1.206
BKK23980	*artP*	-1.522	1.136	0.113	432	1.153
BKK02390	*ilvE*	-1.528	1.142	0.099	480	1.160
BKK16930	*cinA*	-1.529	1.146	0.103	109	1.164
BKK08300	*yfiK*	-1.533	1.141	0.116	123	1.159
BKK21860	*yphP*	-1.535	1.137	0.102	356	1.155
BKK06078	*ydzW*	-1.536	1.130	0.102	158	1.147
BKK26440	*yrkO*	-1.536	1.159	0.108	235	1.177
BKK39630	*yxdM*	-1.539	1.203	0.082	74	1.222
BKK21760	*ypmP*	-1.542	1.137	0.112	541	1.155
BKK03620	*yclA*	-1.543	1.147	0.102	218	1.165
BKK29900	*trmB*	-1.543	1.143	0.115	339	1.161
BKK30100	*yteT*	-1.544	1.138	0.084	264	1.156
BKK38450	*ywaE*	-1.544	1.140	0.095	86	1.158
BKK21930	*cspD*	-1.545	1.136	0.115	292	1.153
BKK37840	*spsI*	-1.546	1.148	0.109	96	1.166
BKK41060	*rpmH*	-1.546	1.138	0.141	37	1.156
BKK22550	*qcrB*	-1.549	1.083	0.122	393	1.100
BKK23600	*yqxK*	-1.551	1.169	0.102	227	1.187
BKK02460	*ycbC*	-1.554	1.112	0.103	106	1.130
BKK16100	*sucD*	-1.556	1.144	0.103	270	1.163
BKK25330	*yqfF*	-1.559	1.145	0.095	287	1.164
BKK02790	*ycdB*	-1.56	1.112	0.101	71	1.130
BKK38510	*dltB*	-1.561	1.148	0.109	108	1.166
BKK02340	*gltP*	-1.571	1.112	0.086	113	1.130
BKK13380	*ykoS*	-1.571	1.150	0.104	463	1.168
BKK31730	*yuzC*	-1.571	1.122	0.085	67	1.139
BKK11809	*yjcZ*	-1.572	1.170	0.088	328	1.189
BKK30720	*ythB*	-1.572	1.138	0.101	112	1.156
BKK40040	*glxK*	-1.572	1.129	0.101	458	1.147
BKK10120	*hemE*	-1.574	1.158	0.118	98	1.177
BKK29710	*acuC*	-1.577	1.133	0.106	278	1.151
BKK11910	*yjcM*	-1.578	1.170	0.112	321	1.189
BKK33222	*rsoA*	-1.578	1.116	0.082	107	1.134
BKK35360	*hag*	-1.58	1.117	0.112	490	1.135
BKK33610	*rnr*	-1.581	1.116	0.098	95	1.134
BKK17770	*yndF*	-1.588	1.145	0.107	100	1.164
BKK23090	*rsiX*	-1.588	1.138	0.103	140	1.156
BKK40680	*yybD*	-1.588	1.166	0.095	235	1.184
BKK08170	*yfjA*	-1.59	1.140	0.107	392	1.159
BKK16130	*trmFO*	-1.592	1.141	0.096	555	1.160
BKK28110	*spoVID*	-1.593	1.141	0.104	89	1.160
BKK29010	*speD*	-1.593	1.095	0.102	167	1.113
BKK20450	*yorA*	-1.596	1.149	0.108	282	1.168
BKK13050	*ykzH*	-1.597	1.144	0.101	351	1.163
BKK07670	*yflI*	-1.6	1.141	0.099	508	1.160
BKK19410	*cwlS*	-1.603	1.143	0.119	379	1.162
BKK03540	*ycxB*	-1.605	1.112	0.097	128	1.130
BKK20040	*yosP*	-1.606	1.111	0.146	181	1.129
BKK07850	*yfkM*	-1.608	1.141	0.100	442	1.160
BKK32640	*sspG*	-1.609	1.116	0.080	162	1.134
BKK36600	*mta*	-1.609	1.096	0.102	68	1.114
BKK10560	*yhjM*	-1.611	1.164	0.102	93	1.183
BKK17940	*yneI*	-1.612	1.143	0.103	382	1.162
BKK03510	*srfAC*	-1.613	1.112	0.105	104	1.130
BKK24850	*glcK*	-1.614	1.145	0.097	126	1.164
BKK11570	*yjbJ*	-1.617	1.189	0.136	144	1.208
BKK09600	*crcBA*	-1.619	1.158	0.098	143	1.177
BKK05890	*ydhU*	-1.62	1.139	0.095	125	1.158
BKK08900	*yhzB*	-1.623	1.158	0.096	73	1.177
BKK06840	*yeeI*	-1.629	1.160	0.112	307	1.179
BKK25880	*yqxJ*	-1.632	0.959	0.070	80	0.975
BKK01530	*cwlD*	-1.634	0.959	0.076	49	0.975
BKK25580	*comEB*	-1.639	1.144	0.109	305	1.164
BKK38270	*efeM*	-1.641	1.131	0.107	296	1.149
BKK08490	*yfhD*	-1.643	1.140	0.101	197	1.159
BKK20580	*yoqM*	-1.644	1.136	0.098	448	1.155
BKK34590	*mdxG*	-1.644	1.131	0.110	557	1.149
BKK30150	*ytdP*	-1.647	1.137	0.087	99	1.156
BKK37910	*spsA*	-1.648	1.138	0.131	166	1.157
BKK33860	*yvbH*	-1.653	1.142	0.107	283	1.161
BKK39890	*yxbB*	-1.653	1.138	0.121	98	1.158
BKK32050	*pepA*	-1.655	1.129	0.097	189	1.148
BKK07620	*yflN*	-1.658	1.140	0.111	437	1.159
BKK07955	*yfkA*	-1.658	1.143	0.102	358	1.163
BKK08340	*padR*	-1.659	1.143	0.103	324	1.163
BKK26550	*yrkD*	-1.661	1.157	0.102	241	1.177
BKK28590	*polX*	-1.661	1.140	0.094	41	1.160
BKK14280	*moeA*	-1.667	1.159	0.105	186	1.179
BKK12420	*yjoB*	-1.668	1.169	0.115	635	1.189
BKK02270	*pssA*	-1.669	1.111	0.092	91	1.130
BKK33340	*sspJ*	-1.67	1.142	0.113	342	1.161
BKK21890	*ypgQ*	-1.672	1.136	0.106	342	1.155
BKK07710	*yflE*	-1.674	1.119	0.100	128	1.138
BKK32010	*besA*	-1.675	1.129	0.126	164	1.148
BKK38680	*yxlD*	-1.675	1.147	0.106	219	1.166
BKK13520	*ykrP*	-1.676	1.147	0.103	230	1.166
BKK14470	*mreBH*	-1.677	1.143	0.105	317	1.163
BKK26480	*yrkK*	-1.677	1.134	0.097	437	1.153

Sup. Table 4: Cell width of mutants of the BKK collection (continued)

BKK name [1]	gene	screening delta^2 (%)	average width (µm)	+/-	nb	AWP
BKK17030	*cotE*	-1.679	1.186	0.120	146	1.206
BKK29320	*ytnI*	-1.68	1.094	0.124	219	1.113
BKK32240	*thrB*	-1.681	1.129	0.128	97	1.148
BKK10040	*ecsA*	-1.685	1.119	0.106	117	1.138
BKK00140	*dck*	-1.686	1.159	0.111	494	1.179
BKK21960	*ypeQ*	-1.686	1.132	0.102	312	1.151
BKK27270	*mtnN*	-1.686	1.138	0.105	125	1.158
BKK08060	*acoA*	-1.69	1.148	0.100	355	1.168
BKK29720	*ytxE*	-1.693	1.120	0.098	134	1.139
BKK00480	*yabJ*	-1.696	1.144	0.104	133	1.164
BKK16720	*ymxH*	-1.696	1.144	0.110	151	1.164
BKK09780	*yheC*	-1.699	1.157	0.100	171	1.177
BKK39990	*yxaF*	-1.699	1.145	0.116	71	1.165
BKK12280	*rex*	-1.7	1.143	0.111	267	1.163
BKK33960	*araE*	-1.7	1.142	0.099	359	1.161
BKK34970	*ppaX*	-1.7	1.116	0.123	252	1.135
BKK34600	*mdxF*	-1.704	1.116	0.102	143	1.135
BKK18710	*yozF*	-1.707	1.152	0.106	89	1.172
BKK02619	*yczK*	-1.713	1.111	0.105	61	1.130
BKK31560	*yufP*	-1.713	1.146	0.104	163	1.166
BKK05530	*ydfR*	-1.715	1.140	0.104	319	1.160
BKK39840	*yxbG*	-1.716	0.958	0.091	42	0.975
BKK03690	*yczF*	-1.718	1.145	0.106	134	1.165
BKK32560	*frlR*	-1.72	1.120	0.092	204	1.139
BKK30120	*yteR*	-1.721	1.136	0.089	121	1.156
BKK19890	*yotG*	-1.724	1.110	0.125	487	1.129
BKK11330	*fabHA*	-1.725	1.163	0.114	77	1.183
BKK24640	*yqxM*	-1.725	1.131	0.100	200	1.151
BKK36830	*atpA*	-1.727	1.095	0.114	135	1.114
BKK32460	*pucM*	-1.728	1.128	0.139	50	1.148
BKK12940	*dppC*	-1.729	1.187	0.151	94	1.208
BKK26860	*yraO*	-1.73	1.156	0.093	212	1.177
BKK38240	*ywcA*	-1.732	1.099	0.144	41	1.119
BKK25620	*yqeL*	-1.74	1.143	0.093	205	1.164
BKK17690	*yncM*	-1.741	1.142	0.097	467	1.162
BKK04740	*rsbX*	-1.745	1.145	0.137	276	1.165
BKK31440	*patB*	-1.745	1.128	0.096	187	1.148
BKK32870	*yusO*	-1.745	1.115	0.106	105	1.134
BKK07420	*yfmM*	-1.75	1.187	0.136	44	1.208
BKK34140	*ganQ*	-1.75	1.137	0.087	145	1.158
BKK05990	*tatCY*	-1.759	1.139	0.110	489	1.160
BKK17880	*ynzC*	-1.766	1.141	0.100	341	1.162
BKK21640	*yokC*	-1.773	1.133	0.100	472	1.153
BKK39710	*iolF*	-1.773	1.146	0.099	194	1.166
BKK23830	*yqjL*	-1.776	1.166	0.112	135	1.187
BKK22300	*yppC*	-1.778	1.081	0.120	153	1.100
BKK33500	*copA*	-1.778	1.118	0.101	103	1.138
BKK04270	*ydaJ*	-1.78	1.180	0.070	54	1.201
BKK19999	*yojW*	-1.781	1.109	0.134	310	1.129
BKK24080	*bcd*	-1.787	1.166	0.112	291	1.187
BKK11970	*yjcS*	-1.79	1.142	0.111	337	1.163
BKK30595	*ytlA*	-1.791	1.200	0.059	243	1.222
BKK21110	*yonF*	-1.793	1.134	0.116	243	1.155
BKK32480	*pucD*	-1.793	1.145	0.108	84	1.166
BKK13990	*kinA*	-1.798	1.163	0.113	98	1.184
BKK05780	*ydhJ*	-1.799	1.139	0.099	444	1.160
BKK32360	*yunC*	-1.799	1.127	0.115	101	1.148
BKK03981	*mtlA*	-1.801	1.144	0.089	125	1.165
BKK10940	*yitC*	-1.801	1.162	0.112	128	1.183
BKK15720	*defA*	-1.801	1.114	0.115	85	1.134
BKK26740	*cypA*	-1.802	1.155	0.101	376	1.177
BKK35090	*yvmA*	-1.802	1.129	0.097	453	1.149
BKK08100	*acoR*	-1.805	1.138	0.098	462	1.159
BKK12390	*uxaA*	-1.805	1.147	0.105	422	1.168
BKK39390	*hutM*	-1.809	1.144	0.107	106	1.165
BKK22930	*sleB*	-1.81	1.080	0.128	100	1.100
BKK07430	*yfmL*	-1.811	1.139	0.109	434	1.160
BKK22810	*yphF*	-1.812	1.185	0.126	147	1.206
BKK07530	*yfmB*	-1.815	1.138	0.106	496	1.159
BKK36660	*ureA*	-1.815	1.094	0.121	218	1.114
BKK33850	*yvbG*	-1.819	0.957	0.071	65	0.975
BKK14110	*ykuK*	-1.821	1.141	0.111	232	1.163
BKK34160	*cycB*	-1.821	1.129	0.113	428	1.149
BKK34820	*bmrA*	-1.823	1.128	0.094	590	1.149
BKK25600	*comER*	-1.825	1.142	0.094	229	1.164
BKK01060	*ybxB*	-1.83	1.142	0.097	388	1.164
BKK06079	*ydzW*	-1.832	1.136	0.101	98	1.158
BKK15460	*rluD*	-1.835	1.093	0.110	129	1.113
BKK31850	*yueC*	-1.837	1.127	0.115	237	1.148

BKK name [1]	gene	screening delta^2 (%)	average width (µm)	+/-	nb	AWP
BKK38180	*ywzA*	-1.84	1.136	0.100	88	1.157
BKK03140	*tmrB*	-1.844	1.109	0.093	113	1.130
BKK10080	*yhfA*	-1.844	1.186	0.151	72	1.208
BKK24580	*yqhH*	-1.844	1.136	0.093	114	1.158
BKK11820	*yjcD*	-1.85	1.141	0.099	347	1.163
BKK19350	*yocS*	-1.853	1.184	0.144	83	1.206
BKK40170	*yydG*	-1.854	1.146	0.126	284	1.168
BKK39750	*iolB*	-1.855	1.143	0.113	74	1.165
BKK36350	*ywpD*	-1.858	1.140	0.101	406	1.161
BKK39180	*yxiH*	-1.859	1.143	0.095	72	1.165
BKK39970	*yxaH*	-1.859	1.128	0.097	551	1.149
BKK11790	*yjcA*	-1.861	1.167	0.132	759	1.189
BKK01935	*skfC*	-1.866	1.092	0.095	102	1.113
BKK16650	*rbfA*	-1.867	1.142	0.135	97	1.164
BKK21329	*youB*	-1.867	1.132	0.099	450	1.153
BKK34460	*levB*	-1.867	1.114	0.116	246	1.135
BKK37710	*bacD*	-1.868	1.128	0.095	609	1.149
BKK03890	*gabR*	-1.873	1.143	0.129	557	1.165
BKK06570	*yerB*	-1.876	1.179	0.088	145	1.201
BKK04910	*yddB*	-1.881	1.126	0.096	417	1.147
BKK32000	*dhbA*	-1.881	1.118	0.089	39	1.139
BKK39080	*licT*	-1.883	1.143	0.098	69	1.165
BKK26559	*yrzN*	-1.884	1.155	0.108	337	1.177
BKK20420	*yorD*	-1.887	1.140	0.105	457	1.162
BKK38140	*qoxD*	-1.887	1.135	0.089	126	1.157
BKK01240	*rpmC*	-1.888	1.126	0.106	154	1.147
BKK07070	*yesY*	-1.888	1.157	0.135	181	1.179
BKK32370	*yunD*	-1.889	1.126	0.118	44	1.148
BKK37230	*ywjA*	-1.892	1.126	0.106	474	1.147
BKK20780	*yopS*	-1.893	1.140	0.105	463	1.162
BKK18740	*yozG*	-1.894	1.149	0.125	113	1.172
BKK09259	*yhzG*	-1.897	1.141	0.101	340	1.163
BKK17860	*yneA*	-1.9	1.141	0.121	79	1.164
BKK25960	*yqcB*	-1.901	1.141	0.112	132	1.164
BKK22330	*ypoC*	-1.905	1.129	0.101	491	1.151
BKK37700	*bacE*	-1.91	1.127	0.113	257	1.149
BKK02970	*yceK*	-1.913	1.108	0.089	99	1.130
BKK01750	*ybbP*	-1.915	1.137	0.087	209	1.160
BKK25130	*nfo*	-1.917	1.141	0.097	250	1.164
BKK29450	*argG*	-1.919	1.129	0.102	330	1.151
BKK08960	*yhbF*	-1.923	1.154	0.096	209	1.177
BKK26570	*yrkB*	-1.923	1.131	0.102	355	1.153
BKK16250	*fliJ*	-1.927	1.146	0.102	344	1.168
BKK21590	*yokH*	-1.929	1.133	0.109	426	1.155
BKK26075	*yqbK*	-1.933	1.129	0.105	474	1.151
BKK06420	*purE*	-1.938	1.156	0.124	258	1.179
BKK40310	*phrG*	-1.938	1.129	0.097	459	1.151
BKK13790	*ykvQ*	-1.939	1.144	0.107	90	1.166
BKK33100	*liaF*	-1.941	1.112	0.095	174	1.134
BKK27380	*yrzB*	-1.946	1.129	0.102	372	1.151
BKK02150	*ybeF*	-1.948	1.108	0.113	74	1.130
BKK13240	*thiU*	-1.95	1.144	0.117	52	1.166
BKK17540	*ynaF*	-1.95	1.139	0.107	360	1.162
BKK02070	*csgA*	-1.952	1.108	0.093	59	1.130
BKK28410	*gerE*	-1.952	1.137	0.093	132	1.160
BKK02060	*ybxG*	-1.953	1.108	0.078	45	1.130
BKK30240	*bioW*	-1.956	1.133	0.098	264	1.156
BKK14580	*pdhA*	-1.958	1.156	0.161	118	1.179
BKK35540	*tuaH*	-1.961	1.127	0.100	691	1.149
BKK35630	*lytB*	-1.961	1.127	0.100	691	1.149
BKK19330	*sodF*	-1.968	1.149	0.112	116	1.172
BKK37140	*ywjG*	-1.968	1.097	0.112	690	1.119
BKK31300	*yugS*	-1.97	1.138	0.102	296	1.161
BKK38499	*ywzH*	-1.972	1.097	0.122	85	1.119
BKK24280	*ispA*	-1.974	1.164	0.099	281	1.187
BKK25040	*yqgB*	-1.974	1.141	0.114	149	1.164
BKK36530	*bcrC*	-1.975	1.092	0.073	51	1.114
BKK03730	*yclH*	-1.976	1.178	0.116	979	1.201
BKK27760	*csbX*	-1.977	1.137	0.113	408	1.160
BKK15330	*sigG*	-1.978	1.117	0.114	66	1.139
BKK00350	*yazA*	-1.982	1.156	0.155	152	1.179
BKK14420	*ykoA*	-1.984	1.156	0.117	450	1.179
BKK24710	*comGC*	-1.985	1.140	0.091	464	1.164
BKK32940	*yusV*	-1.985	1.198	0.098	38	1.222
BKK35240	*ctpB*	-1.988	1.113	0.131	420	1.135
BKK13130	*proA*	-1.989	1.143	0.113	173	1.166
BKK16790	*tepA*	-1.99	1.140	0.117	96	1.164
BKK27590	*dtd*	-1.991	1.137	0.095	316	1.160
BKK08570	*yfhK*	-1.996	1.136	0.118	187	1.159

BKK name [1]	gene	screening delta^2 (%)	average width (µm)	+/-	nb	AWP
BKK32020	*yuiH*	-2.006	1.125	0.107	138	1.148
BKK35100	*yvlD*	-2.007	1.112	0.106	409	1.135
BKK37500	*speE*	-2.01	1.096	0.135	44	1.119
BKK12740	*xkdU*	-2.012	1.143	0.120	135	1.166
BKK33140	*yvqJ*	-2.017	1.197	0.083	140	1.222
BKK27040	*levG*	-2.019	1.153	0.110	255	1.177
BKK06036	*ydzT*	-2.026	1.136	0.101	346	1.160
BKK17210	*pksN*	-2.03	1.140	0.106	81	1.164
BKK20600	*yoqK*	-2.031	1.131	0.114	250	1.155
BKK05550	*cotP*	-2.033	1.124	0.104	346	1.147
BKK20230	*yorW*	-2.033	1.144	0.114	250	1.168
BKK02640	*tatCD*	-2.034	1.177	0.071	79	1.201
BKK32140	*paiB*	-2.035	1.125	0.098	42	1.148
BKK20120	*yosH*	-2.036	1.106	0.120	100	1.129
BKK09190	*yhcR*	-2.037	1.153	0.104	120	1.177
BKK12570	*xtmA*	-2.037	1.165	0.129	436	1.189
BKK39900	*aslA*	-2.041	1.142	0.108	112	1.166
BKK24900	*rpmGA*	-2.043	1.140	0.095	211	1.164
BKK34550	*pgcM*	-2.046	1.112	0.109	267	1.135
BKK00560	*spoVT*	-2.048	0.955	0.075	44	0.975
BKK21000	*yonT*	-2.049	1.130	0.105	585	1.153
BKK31319	*yuzI*	-2.049	1.132	0.113	69	1.156
BKK24490	*yqhQ*	-2.05	1.163	0.094	377	1.187
BKK28780	*araD*	-2.051	1.090	0.092	351	1.113
BKK25920	*yqxG*	-2.054	1.140	0.130	58	1.164
BKK18590	*yoaG*	-2.055	1.115	0.094	104	1.138
BKK05840	*gmuD*	-2.056	1.143	0.077	244	1.167
BKK09110	*yhcJ*	-2.056	1.183	0.142	691	1.208
BKK27660	*comN*	-2.058	1.136	0.107	232	1.160
BKK20710	*yopZ*	-2.06	1.131	0.104	381	1.155
BKK21010	*yonS*	-2.06	1.182	0.106	94	1.206
BKK33250	*yvrL*	-2.06	1.137	0.104	253	1.161
BKK02730	*natK*	-2.062	1.177	0.059	52	1.201
BKK30360	*yttA*	-2.065	1.132	0.101	148	1.156
BKK05820	*gmuA*	-2.068	1.124	0.095	395	1.147
BKK26530	*yrkF*	-2.07	1.130	0.109	398	1.153
BKK35239	*swrAA*	-2.071	1.112	0.123	410	1.135
BKK37740	*bacA*	-2.072	1.096	0.098	90	1.119
BKK15630	*sirC*	-2.074	1.124	0.112	433	1.148
BKK13560	*mtnK*	-2.076	1.138	0.107	430	1.163
BKK21130	*yonD*	-2.079	1.131	0.097	406	1.155
BKK30870	*ytcB*	-2.08	1.197	0.063	445	1.222
BKK12240	*yjkA*	-2.087	1.183	0.158	459	1.208
BKK05800	*pbuE*	-2.088	1.176	0.051	59	1.201
BKK06000	*ydiK*	-2.091	1.143	0.098	204	1.167
BKK11890	*yjcK*	-2.094	1.164	0.121	356	1.189
BKK06190	*ydjG*	-2.096	1.154	0.103	168	1.179
BKK05760	*ydhH*	-2.098	1.143	0.131	139	1.167
BKK24700	*comGD*	-2.099	1.127	0.104	387	1.151
BKK33970	*araR*	-2.101	1.133	0.091	124	1.158
BKK12840	*pit*	-2.103	1.183	0.132	80	1.208
BKK34740	*crh*	-2.105	1.111	0.094	379	1.135
BKK26690	*brnQ*	-2.114	1.196	0.086	222	1.222
BKK02320	*ybfP*	-2.117	1.106	0.103	125	1.130
BKK37490	*speB*	-2.117	1.095	0.099	57	1.119
BKK07520	*yfmC*	-2.119	1.183	0.125	181	1.208
BKK12480	*yjqB*	-2.126	1.164	0.147	729	1.189
BKK36090	*ywrE*	-2.126	1.091	0.098	458	1.114
BKK07460	*yfmI*	-2.131	1.135	0.110	546	1.160
BKK25670	*yqeH*	-2.131	1.127	0.133	130	1.151
BKK37800	*yweA*	-2.137	1.125	0.105	439	1.149
BKK08240	*catE*	-2.141	1.134	0.109	292	1.159
BKK04600	*ydbT*	-2.146	1.123	0.098	421	1.147
BKK30220	*bioF*	-2.15	1.131	0.103	114	1.156
BKK31780	*yueH*	-2.152	1.123	0.107	205	1.148
BKK06790	*yeeD*	-2.157	1.135	0.096	487	1.160
BKK21430	*bhlB*	-2.158	1.130	0.103	474	1.155
BKK18450	*gltA*	-2.159	1.113	0.087	57	1.138
BKK10920	*yitA*	-2.163	1.158	0.114	121	1.183
BKK38420	*sacY*	-2.163	1.095	0.113	183	1.119
BKK01610	*feuC*	-2.166	1.151	0.095	232	1.177
BKK38390	*ywbA*	-2.167	1.132	0.124	61	1.157
BKK16010	*ylqD*	-2.168	1.132	0.107	126	1.157
BKK24030	*bkdB*	-2.168	1.161	0.108	173	1.187
BKK22130	*kduI*	-2.171	1.076	0.137	144	1.100
BKK29520	*yteJ*	-2.178	1.089	0.105	72	1.113
BKK09670	*dat*	-2.179	1.182	0.127	45	1.208
BKK32060	*yuiD*	-2.18	1.115	0.109	82	1.139
BKK25850	*yqzI*	-2.181	1.138	0.112	104	1.164

BKK name [1]	gene	screening delta^2 (%)	average width (µm)	+/-	nb	AWP
BKK06350	*yebA*	-2.185	1.153	0.110	460	1.179
BKK13190	*ispA*	-2.185	1.141	0.085	56	1.166
BKK03550	*ycxC*	-2.186	1.105	0.097	113	1.130
BKK36070	*cotG*	-2.187	1.131	0.117	91	1.156
BKK17250	*ymaE*	-2.189	1.136	0.107	575	1.162
BKK26749	*yrdD*	-2.189	1.151	0.097	166	1.177
BKK32210	*yuzD*	-2.189	1.123	0.093	38	1.148
BKK05240	*ydeL*	-2.19	1.114	0.105	58	1.139
BKK01400	*rpmJ*	-2.192	1.134	0.129	188	1.160
BKK11480	*yjbB*	-2.195	1.182	0.120	87	1.208
BKK28580	*mutSB*	-2.198	1.134	0.107	46	1.160
BKK31840	*yueD*	-2.199	1.123	0.093	145	1.148
BKK15030	*ylbJ*	-2.2	1.153	0.138	261	1.179
BKK26220	*yqaQ*	-2.2	1.151	0.094	408	1.177
BKK40090	*ahpC*	-2.201	1.142	0.109	340	1.168
BKK26000	*yqbR*	-2.206	1.126	0.092	459	1.151
BKK37270	*narH*	-2.206	1.094	0.108	90	1.119
BKK25630	*yqeK*	-2.213	1.138	0.099	142	1.164
BKK34830	*yvaJ*	-2.215	1.110	0.127	164	1.135
BKK11360	*appD*	-2.22	1.182	0.143	116	1.208
BKK25540	*gpr*	-2.22	1.138	0.093	189	1.164
BKK04470	*dctP*	-2.221	1.132	0.095	228	1.158
BKK05329	*ydzO*	-2.222	1.141	0.107	146	1.167
BKK33770	*spbC*	-2.223	1.135	0.097	572	1.161
BKK13150	*ohrR*	-2.225	1.140	0.125	195	1.166
BKK01760	*ybbR*	-2.226	1.134	0.104	558	1.160
BKK11220	*argD*	-2.235	1.142	0.109	390	1.168
BKK19150	*yocB*	-2.239	1.136	0.103	468	1.162
BKK34510	*yvdQ*	-2.239	1.135	0.101	418	1.161
BKK40200	*yydD*	-2.239	1.125	0.102	270	1.151
BKK30440	*ytrC*	-2.249	1.195	0.088	239	1.222
BKK18530	*yoaA*	-2.25	1.132	0.108	113	1.158
BKK18780	*yoaW*	-2.251	1.145	0.095	267	1.172
BKK00680	*hprT*	-2.252	1.122	0.103	265	1.147
BKK11810	*spoVIF*	-2.255	1.162	0.121	413	1.189
BKK37770	*rocB*	-2.255	1.094	0.123	46	1.119
BKK02890	*yceC*	-2.256	1.131	0.095	132	1.158
BKK29140	*citZ*	-2.261	1.088	0.138	132	1.113
BKK39560	*yxeG*	-2.262	1.138	0.092	115	1.165
BKK34170	*ganR*	-2.265	1.109	0.121	92	1.135
BKK09870	*khtS*	-2.266	1.181	0.180	175	1.208
BKK25750	*nucB*	-2.267	0.953	0.064	47	0.975
BKK26619	*yrzO*	-2.271	1.150	0.109	114	1.177
BKK05510	*ydfQ*	-2.272	1.141	0.106	647	1.167
BKK07640	*yflL*	-2.272	1.132	0.106	495	1.159
BKK13500	*ktrD*	-2.274	1.140	0.101	289	1.166
BKK15050	*ylbL*	-2.275	1.114	0.107	81	1.139
BKK00550	*mfd*	-2.278	1.137	0.113	86	1.164
BKK20810	*yopP*	-2.28	1.125	0.108	268	1.151
BKK27050	*levF*	-2.282	1.150	0.094	349	1.177
BKK07970	*yfjT*	-2.289	1.132	0.114	252	1.159
BKK02720	*yccF*	-2.29	1.174	0.082	45	1.201
BKK36580	*ywnF*	-2.291	1.089	0.141	214	1.114
BKK13980	*pbpH*	-2.294	1.136	0.107	403	1.163
BKK38810	*msmX*	-2.294	1.181	0.162	257	1.208
BKK30930	*ytaB*	-2.296	1.135	0.100	429	1.161
BKK13930	*splB*	-2.299	1.140	0.131	47	1.166
BKK33410	*yvgO*	-2.3	1.108	0.079	85	1.134
BKK21650	*yokB*	-2.301	1.127	0.106	388	1.153
BKK26558	*yrzM*	-2.302	1.127	0.104	248	1.153
BKK39250	*yxiE*	-2.304	1.138	0.117	85	1.165
BKK10470	*yhjD*	-2.305	1.136	0.106	422	1.163
BKK28930	*lytS*	-2.308	1.125	0.115	236	1.151
BKK10090	*yhgB*	-2.313	1.150	0.096	126	1.177
BKK00040	*recF*	-2.314	1.140	0.135	688	1.167
BKK04580	*cshA*	-2.315	1.133	0.121	225	1.160
BKK17960	*yneK*	-2.316	1.135	0.112	478	1.162
BKK33350	*yvsG*	-2.316	1.108	0.100	127	1.134
BKK01820	*adaB*	-2.321	1.133	0.101	151	1.160
BKK26230	*yqaP*	-2.327	1.149	0.086	152	1.177
BKK39000	*yxjC*	-2.327	1.138	0.102	117	1.165
BKK26130	*yqbF*	-2.33	0.952	0.105	50	0.975
BKK10860	*yisT*	-2.332	1.156	0.106	103	1.183
BKK20770	*yopT*	-2.332	1.135	0.103	225	1.162
BKK17150	*pksG*	-2.333	1.136	0.105	116	1.164
BKK38570	*licA*	-2.335	1.138	0.092	79	1.165
BKK22860	*yphA*	-2.337	1.178	0.119	103	1.206
BKK28920	*lytT*	-2.346	1.193	0.093	69	1.222
BKK39870	*yxbD*	-2.348	1.137	0.097	99	1.165

Sup. Table 4: Cell width of mutants of the BKK collection (continued)

BKK name [1]	gene	screening delta^2 (%)	average width (µm)	+/-	nb	AWP
BKK33420	*nhaK*	-2.351	1.134	0.101	490	1.161
BKK14710	*ylaA*	-2.355	1.151	0.113	150	1.179
BKK19990	*yosV*	-2.356	1.141	0.108	317	1.168
BKK32470	*pucE*	-2.357	1.113	0.096	129	1.139
BKK01470	*ybaF*	-2.358	1.136	0.092	172	1.164
BKK05560	*ydgA*	-2.36	1.130	0.103	167	1.158
BKK12110	*yjfA*	-2.36	1.161	0.135	210	1.189
BKK40180	*yydF*	-2.363	1.140	0.113	245	1.168
BKK10280	*yhfM*	-2.366	1.155	0.091	112	1.183
BKK39820	*htpG*	-2.366	1.137	0.086	112	1.165
BKK01350	*rplO*	-2.372	1.136	0.099	135	1.164
BKK38860	*galE*	-2.375	1.137	0.119	81	1.165
BKK29110	*phoP*	-2.376	1.087	0.104	34	1.113
BKK29239	*ytrH*	-2.378	1.087	0.080	70	1.113
BKK16450	*cheC*	-2.379	1.134	0.102	380	1.161
BKK15540	*pyrD*	-2.384	1.128	0.122	73	1.156
BKK19259	*yoyB*	-2.384	1.144	0.108	70	1.172
BKK20220	*yorX*	-2.386	1.112	0.112	59	1.139
BKK14390	*fruK*	-2.387	1.151	0.141	131	1.179
BKK20380	*yorH*	-2.39	1.102	0.124	48	1.129
BKK25980	*yqbT*	-2.392	0.952	0.072	120	0.975
BKK39580	*yxeE*	-2.394	1.137	0.089	128	1.165
BKK17050	*mutL*	-2.398	1.136	0.104	144	1.164
BKK16620	*rplGA*	-2.399	1.130	0.100	81	1.158
BKK06049	*ydzV*	-2.404	1.139	0.111	125	1.167
BKK14640	*yktA*	-2.404	1.135	0.110	295	1.163
BKK30270	*msmE*	-2.414	1.111	0.096	110	1.138
BKK37300	*ywiC*	-2.416	1.092	0.110	94	1.119
BKK12920	*dppA*	-2.417	1.138	0.135	52	1.166
BKK27800	*yrzH*	-2.42	1.123	0.114	398	1.151
BKK31322	*yugO*	-2.421	1.128	0.104	48	1.156
BKK27160	*cypB*	-2.422	1.148	0.110	280	1.177
BKK26610	*yrkA*	-2.425	1.148	0.121	121	1.177
BKK24740	*yqxL*	-2.426	1.177	0.128	71	1.206
BKK20960	*yopA*	-2.427	1.127	0.105	270	1.155
BKK30280	*amyD*	-2.427	1.110	0.101	68	1.138
BKK07040	*yesV*	-2.431	1.150	0.111	337	1.179
BKK22800	*spoIVA*	-2.434	1.112	0.092	55	1.139
BKK25510	*lepA*	-2.435	1.135	0.103	312	1.164
BKK26750	*yrdD*	-2.437	1.148	0.105	326	1.177
BKK35110	*yvlC*	-2.438	1.107	0.103	428	1.135
BKK10590	*yhjP*	-2.439	1.134	0.100	274	1.163
BKK27870	*nadB*	-2.441	1.131	0.099	246	1.160
BKK08040	*yfjM*	-2.442	1.131	0.097	529	1.159
BKK19480	*yojE*	-2.442	1.102	0.101	828	1.129
BKK12310	*exuM*	-2.445	1.179	0.120	75	1.208
BKK18880	*yobE*	-2.448	1.133	0.117	361	1.162
BKK27240	*yrhC*	-2.448	1.148	0.109	166	1.177
BKK03450	*hxlB*	-2.454	1.102	0.094	30	1.130
BKK17900	*yneE*	-2.456	1.135	0.085	105	1.164
BKK12800	*xhlB*	-2.463	1.138	0.129	184	1.166
BKK10840	*degA*	-2.473	1.154	0.100	142	1.183
BKK32650	*yurS*	-2.474	1.137	0.105	138	1.166
BKK34030	*yvbY*	-2.474	1.106	0.091	88	1.134
BKK13660	*kinD*	-2.476	1.177	0.160	152	1.206
BKK30210	*bioD*	-2.479	1.127	0.118	81	1.156
BKK11928	*yjzF*	-2.488	1.139	0.105	531	1.168
BKK17360	*ymzA*	-2.491	1.135	0.107	132	1.164
BKK38740	*cydC*	-2.491	1.172	0.129	459	1.201
BKK14490	*kinC*	-2.494	1.176	0.151	48	1.206
BKK39300	*yxiD*	-2.497	1.137	0.094	81	1.166
BKK12350	*uxuB*	-2.498	1.159	0.141	177	1.189
BKK16350	*fliP*	-2.498	1.133	0.108	581	1.162
BKK15610	*sumT*	-2.501	1.119	0.100	165	1.148
BKK30880	*ytcC*	-2.505	1.132	0.107	301	1.161
BKK40050	*gntR*	-2.516	1.119	0.119	149	1.147
BKK12360	*exuT*	-2.521	1.178	0.149	104	1.208
BKK18940	*yozK*	-2.523	1.142	0.105	362	1.172
BKK19460	*yojG*	-2.523	1.101	0.120	194	1.129
BKK03850	*ycnC*	-2.524	1.136	0.098	318	1.165
BKK40750	*yyaQ*	-2.524	1.119	0.106	545	1.147
BKK26820	*yrpD*	-2.527	1.147	0.111	280	1.177
BKK39730	*iolD*	-2.527	1.135	0.108	107	1.165
BKK20150	*yosE*	-2.528	1.101	0.157	129	1.129
BKK32250	*thrC*	-2.529	1.119	0.096	75	1.148
BKK28520	*etfA*	-2.532	1.128	0.107	72	1.158
BKK21340	*yomJ*	-2.533	1.126	0.096	315	1.155
BKK37020	*ywkD*	-2.533	1.086	0.081	34	1.114
BKK22900	*ypfB*	-2.534	1.072	0.109	247	1.100

BKK name [1]	gene	screening delta^2 (%)	average width (µm)	+/-	nb	AWP
BKK27009	*yrzP*	-2.535	1.124	0.106	366	1.153
BKK10670	*gerPF*	-2.539	1.153	0.097	128	1.183
BKK07790	*yfkQ*	-2.54	1.129	0.114	366	1.159
BKK19560	*yodD*	-2.541	1.101	0.129	112	1.129
BKK24260	*yqxC*	-2.542	1.128	0.090	113	1.158
BKK24470	*yqhS*	-2.542	1.128	0.090	113	1.158
BKK05409	*ydzQ*	-2.543	1.118	0.098	434	1.147
BKK26940	*yraH*	-2.544	1.147	0.098	433	1.177
BKK30739	*ytzL*	-2.548	1.127	0.091	60	1.156
BKK10000	*yhaH*	-2.551	1.133	0.101	400	1.163
BKK05310	*ydeR*	-2.553	1.130	0.114	429	1.160
BKK18898	*yozW*	-2.555	1.109	0.093	41	1.138
BKK21770	*ilvA*	-2.556	0.950	0.072	150	0.975
BKK18550	*yoaC*	-2.562	1.109	0.109	53	1.138
BKK12200	*yjiA*	-2.563	1.159	0.083	1736	1.189
BKK08030	*dusC*	-2.566	1.129	0.106	451	1.159
BKK18560	*yoaD*	-2.568	1.132	0.110	415	1.162
BKK18060	*yneR*	-2.577	1.134	0.105	133	1.164
BKK27860	*nadC*	-2.578	1.121	0.115	422	1.151
BKK26600	*bltD*	-2.579	1.146	0.099	140	1.177
BKK28260	*leuC*	-2.582	1.130	0.085	298	1.160
BKK36310	*ssbB*	-2.59	1.085	0.114	158	1.114
BKK40259	*yyzG*	-2.594	1.120	0.120	232	1.149
BKK16810	*ymfC*	-2.598	1.133	0.087	86	1.164
BKK00580	*yabN*	-2.603	1.133	0.093	136	1.164
BKK18170	*yngA*	-2.612	1.141	0.125	150	1.172
BKK36120	*ywrB*	-2.614	1.136	0.096	96	1.166
BKK28900	*lrgB*	-2.618	1.084	0.121	239	1.113
BKK39050	*katE*	-2.621	1.134	0.094	87	1.165
BKK10770	*wprA*	-2.626	1.152	0.118	131	1.183
BKK29170	*ytzA*	-2.626	1.131	0.102	350	1.161
BKK08200	*malP*	-2.633	1.128	0.098	405	1.159
BKK09910	*yhaO*	-2.633	1.153	0.102	172	1.184
BKK17550	*ynaG*	-2.633	1.133	0.151	89	1.164
BKK20630	*yoqH*	-2.633	1.131	0.103	434	1.162
BKK35900	*pgsB*	-2.633	1.119	0.103	461	1.149
BKK06530	*purD*	-2.634	1.148	0.118	479	1.179
BKK40021	*yxzK*	-2.636	1.119	0.089	601	1.149
BKK21440	*bdbB*	-2.643	1.124	0.103	548	1.155
BKK06290	*yeaA*	-2.644	1.148	0.116	159	1.179
BKK35030	*yvoA*	-2.648	1.135	0.113	78	1.166
BKK01380	*mapA*	-2.649	1.133	0.104	327	1.164
BKK22050	*bcsA*	-2.651	1.071	0.103	79	1.100
BKK25140	*cshB*	-2.654	1.133	0.107	190	1.164
BKK33810	*opuCC*	-2.656	1.119	0.101	243	1.149
BKK21680	*msrB*	-2.662	1.124	0.093	567	1.155
BKK03460	*hxlA*	-2.666	1.100	0.096	72	1.130
BKK06800	*yezA*	-2.671	1.147	0.112	331	1.179
BKK14840	*ylaN*	-2.671	1.148	0.089	337	1.179
BKK10570	*yhjN*	-2.674	1.152	0.096	86	1.183
BKK15130	*mraZ*	-2.674	1.108	0.100	130	1.138
BKK04000	*ycsA*	-2.676	1.134	0.109	110	1.165
BKK26748	*yrdD*	-2.68	1.145	0.098	379	1.177
BKK28210	*lonB*	-2.683	1.129	0.093	223	1.160
BKK22460	*ypjH*	-2.687	1.120	0.100	398	1.151
BKK36890	*upp*	-2.692	1.084	0.127	283	1.114
BKK26360	*yqaD*	-2.695	0.949	0.088	33	0.975
BKK33290	*fhuC*	-2.696	1.135	0.092	100	1.166
BKK13370	*ykoQ*	-2.697	1.135	0.109	154	1.166
BKK02080	*ybxH*	-2.698	1.099	0.097	41	1.130
BKK13960	*ykwC*	-2.7	1.135	0.118	328	1.166
BKK31000	*yuaI*	-2.7	1.125	0.095	139	1.156
BKK11280	*yjaU*	-2.71	1.151	0.144	93	1.183
BKK21950	*ypzA*	-2.71	1.124	0.107	354	1.155
BKK40740	*yyaR*	-2.711	1.152	0.101	266	1.184
BKK36610	*ywnC*	-2.717	1.125	0.105	107	1.157
BKK14980	*ylbE*	-2.718	1.147	0.130	424	1.179
BKK23420	*spoVAC*	-2.718	1.120	0.110	328	1.151
BKK37200	*ywjD*	-2.721	0.948	0.072	47	0.975
BKK24880	*yqgO*	-2.728	1.132	0.098	228	1.164
BKK27970	*spoIVFB*	-2.733	1.128	0.102	269	1.160
BKK03470	*hxlR*	-2.74	1.099	0.087	84	1.130
BKK19639	*yoyE*	-2.741	1.098	0.141	180	1.129
BKK12750	*xkdV*	-2.743	1.134	0.127	291	1.166
BKK30860	*ytcA*	-2.746	1.188	0.074	183	1.222
BKK13570	*mtnU*	-2.747	1.134	0.099	260	1.166
BKK19790	*cgeB*	-2.751	1.098	0.091	379	1.129
BKK30260	*msmR*	-2.757	1.124	0.102	89	1.156
BKK34630	*mdxR*	-2.757	1.129	0.091	497	1.161

BKK name [1]	gene	screening delta^2 (%)	average width (µm)	+/-	nb	AWP
BKK17800	*yndJ*	-2.759	1.131	0.125	81	1.164
BKK03569	*sfp*	-2.765	1.099	0.093	46	1.130
BKK15210	*spoVE*	-2.766	1.173	0.125	72	1.206
BKK22280	*yppD*	-2.774	1.070	0.114	281	1.100
BKK29770	*ytxH*	-2.777	1.082	0.121	83	1.113
BKK34770	*yvcJ*	-2.778	1.104	0.114	215	1.135
BKK35730	*tagE*	-2.778	1.134	0.104	114	1.166
BKK15750	*yloN*	-2.779	1.103	0.097	89	1.134
BKK23660	*yqkB*	-2.783	1.154	0.103	246	1.187
BKK25240	*yqfL*	-2.784	1.131	0.102	140	1.164
BKK28380	*gerM*	-2.788	1.127	0.117	165	1.160
BKK00980	*sigH*	-2.792	1.154	0.109	586	1.187
BKK00960	*rlmB*	-2.8	1.154	0.093	297	1.187
BKK24910	*yqgM*	-2.8	1.119	0.105	522	1.151
BKK29830	*ytpQ*	-2.8	1.125	0.105	157	1.158
BKK05109	*ydzN*	-2.801	1.127	0.101	522	1.160
BKK13330	*ykoL*	-2.802	1.108	0.114	39	1.139
BKK23870	*polYA*	-2.803	1.154	0.100	293	1.187
BKK27640	*yrvC*	-2.807	1.127	0.093	225	1.160
BKK00830	*ctsR*	-2.808	1.069	0.121	202	1.100
BKK09890	*natA*	-2.808	1.174	0.151	90	1.208
BKK12020	*manA*	-2.808	1.156	0.138	122	1.189
BKK39370	*hutI*	-2.808	0.948	0.072	30	0.975
BKK36180	*ywqK*	-2.81	1.134	0.098	114	1.166
BKK02600	*cwlJ*	-2.812	1.168	0.061	37	1.201
BKK38370	*ywbC*	-2.813	1.087	0.222	31	1.119
BKK32280	*yutG*	-2.816	1.116	0.105	62	1.148
BKK15640	*yloA*	-2.818	1.116	0.165	31	1.148
BKK27850	*nadA*	-2.823	1.127	0.098	241	1.160
BKK35609	*tuaA*	-2.823	1.117	0.109	518	1.149
BKK14360	*yknY*	-2.829	1.106	0.096	114	1.138
BKK27530	*yrvN*	-2.83	1.127	0.093	414	1.160
BKK32719	*yuzK*	-2.83	1.102	0.097	169	1.134
BKK20490	*yoqW*	-2.843	1.097	0.126	55	1.129
BKK09430	*citR*	-2.849	1.143	0.109	184	1.177
BKK27030	*sacC*	-2.849	1.143	0.095	115	1.177
BKK28180	*ysxD*	-2.85	1.127	0.099	313	1.160
BKK02560	*ycbM*	-2.851	1.098	0.099	170	1.130
BKK07920	*yfkE*	-2.852	1.126	0.101	420	1.159
BKK08630	*yfhQ*	-2.855	1.126	0.105	319	1.159
BKK11290	*yjaV*	-2.86	1.149	0.123	133	1.183
BKK08580	*yfhL*	-2.862	1.126	0.095	507	1.159
BKK40320	*argI*	-2.862	1.118	0.105	351	1.151
BKK20640	*yoqG*	-2.866	1.129	0.102	411	1.162
BKK06510	*purN*	-2.871	1.145	0.103	417	1.179
BKK08090	*acoL*	-2.873	1.129	0.106	386	1.163
BKK25400	*yqeY*	-2.873	1.130	0.103	178	1.164
BKK16845	*ymfF*	-2.877	1.130	0.114	127	1.164
BKK36000	*alsD*	-2.881	1.124	0.100	167	1.157
BKK20340	*yorL*	-2.89	1.097	0.144	56	1.129
BKK31760	*pncA*	-2.894	1.106	0.096	129	1.139
BKK20100	*yosJ*	-2.898	1.097	0.133	51	1.129
BKK00160	*yaaH*	-2.903	1.125	0.122	113	1.159
BKK13590	*mtnW*	-2.908	1.132	0.083	62	1.166
BKK28860	*rpmI*	-2.908	1.118	0.090	208	1.151
BKK36200	*ywqI*	-2.909	1.082	0.114	70	1.114
BKK00660	*yabT*	-2.912	1.096	0.161	81	1.129
BKK37940	*ywdJ*	-2.916	1.086	0.115	64	1.119
BKK40430	*yycE*	-2.918	1.132	0.099	185	1.166
BKK01860	*ybcF*	-2.921	1.126	0.100	422	1.160
BKK07690	*mapB*	-2.928	1.125	0.111	181	1.159
BKK17060	*ymzD*	-2.929	1.129	0.099	74	1.164
BKK21670	*ypqP*	-2.929	1.121	0.104	256	1.155
BKK15170	*spoVD*	-2.931	1.129	0.117	221	1.163
BKK31130	*yubD*	-2.931	1.186	0.079	95	1.222
BKK21100	*yonG*	-2.932	1.117	0.114	347	1.151
BKK27468	*yrzQ*	-2.932	1.126	0.093	407	1.160
BKK21570	*yokJ*	-2.94	1.121	0.112	417	1.155
BKK36020	*alsR*	-2.95	1.081	0.116	336	1.114
BKK26410	*psiE*	-2.953	1.142	0.103	251	1.177
BKK10070	*yhaA*	-2.954	1.142	0.100	150	1.177
BKK30370	*bceB*	-2.959	1.117	0.122	241	1.151
BKK39040	*yxiS*	-2.961	1.130	0.109	60	1.165
BKK21330	*yomK*	-2.963	1.119	0.098	639	1.153
BKK21310	*yozP*	-2.965	1.121	0.112	299	1.155
BKK21910	*metA*	-2.965	1.123	0.107	137	1.158
BKK12610	*xkdG*	-2.968	1.132	0.119	584	1.166
BKK18080	*yneT*	-2.968	1.129	0.103	100	1.164
BKK32469	*yuzJ*	-2.968	1.114	0.108	58	1.148

BKK name [1]	gene	screening delta^2 (%)	average width (µm)	+/-	nb	AWP
BKK06083	*ydzW*	-2.972	1.133	0.102	143	1.167
BKK32330	*lipA*	-2.976	1.127	0.102	309	1.161
BKK20270	*yorS*	-2.98	1.149	0.106	101	1.184
BKK37360	*sboX*	-2.982	1.085	0.096	95	1.119
BKK28270	*leuB*	-2.983	1.125	0.107	318	1.160
BKK40770	*tetB*	-2.985	1.149	0.108	84	1.184
BKK08620	*yfhP*	-2.99	1.124	0.103	526	1.159
BKK40220	*yydB*	-2.99	1.131	0.098	113	1.166
BKK31480	*yuxJ*	-2.992	1.114	0.129	184	1.148
BKK31490	*pbpD*	-2.993	1.185	0.074	103	1.222
BKK37530	*ywhC*	-2.999	1.085	0.097	81	1.119
BKK08670	*ygaB*	-3	1.124	0.115	301	1.159
BKK13420	*ykoW*	-3.003	1.172	0.147	169	1.208
BKK34750	*whiA*	-3.003	1.101	0.144	238	1.135
BKK23329	*ypuC*	-3.016	1.116	0.097	316	1.151
BKK33600	*smpB*	-3.02	1.100	0.093	70	1.134
BKK20060	*nrdEB*	-3.021	1.133	0.108	417	1.168
BKK02820	*rapJ*	-3.026	1.096	0.099	54	1.130
BKK01570	*ybaN*	-3.027	1.141	0.098	283	1.177
BKK20950	*yopB*	-3.03	1.120	0.111	297	1.155
BKK15510	*pyrAA*	-3.032	1.121	0.094	126	1.156
BKK29130	*icd*	-3.037	1.079	0.108	216	1.113
BKK03560	*ycxD*	-3.039	1.096	0.099	61	1.130
BKK40920	*engD*	-3.041	1.148	0.097	110	1.184
BKK09680	*nhaC*	-3.044	1.141	0.116	113	1.177
BKK29020	*gapB*	-3.044	1.079	0.093	142	1.113
BKK12880	*ykcB*	-3.053	1.131	0.111	385	1.166
BKK07200	*yetJ*	-3.058	1.143	0.100	309	1.179
BKK06550	*yecA*	-3.059	1.143	0.108	290	1.179
BKK17070	*ymcC*	-3.059	1.128	0.120	77	1.164
BKK14630	*speA*	-3.064	1.143	0.111	578	1.179
BKK11780	*cotV*	-3.066	1.153	0.125	942	1.189
BKK24590	*yqhG*	-3.067	0.945	0.091	78	0.975
BKK19190	*desK*	-3.071	1.169	0.108	71	1.206
BKK23700	*yqjX*	-3.071	1.151	0.099	168	1.187
BKK27890	*nadR*	-3.074	1.124	0.100	193	1.160
BKK25720	*yqeD*	-3.075	1.116	0.107	389	1.151
BKK09690	*nhaX*	-3.08	1.141	0.099	150	1.177
BKK26090	*yqbJ*	-3.086	1.116	0.102	571	1.151
BKK06900	*cotJB*	-3.088	1.142	0.116	414	1.179
BKK28820	*ysdC*	-3.09	1.104	0.103	125	1.139
BKK17400	*ymaB*	-3.092	1.126	0.106	441	1.162
BKK15670	*ylzA*	-3.11	1.099	0.073	125	1.134
BKK27250	*mccB*	-3.11	1.140	0.114	181	1.177
BKK20880	*yopI*	-3.118	0.945	0.070	69	0.975
BKK31690	*comP*	-3.118	1.130	0.098	91	1.166
BKK39790	*yxcE*	-3.119	1.128	0.075	63	1.165
BKK27520	*cymR*	-3.12	1.123	0.099	388	1.160
BKK24370	*spoIIIAG*	-3.121	1.115	0.095	412	1.151
BKK40520	*yybS*	-3.126	1.130	0.104	157	1.166
BKK11690	*thiG*	-3.13	1.131	0.106	235	1.168
BKK09040	*yhcD*	-3.132	1.140	0.100	215	1.177
BKK22110	*kdgK*	-3.133	1.066	0.117	155	1.100
BKK08710	*gsaB*	-3.15	1.122	0.108	543	1.159
BKK35640	*lytA*	-3.153	1.125	0.107	340	1.161
BKK05343	*ydzS*	-3.154	1.104	0.100	137	1.139
BKK36730	*ywmD*	-3.154	1.079	0.104	233	1.114
BKK10540	*ntdB*	-3.157	1.170	0.147	103	1.208
BKK19170	*yocD*	-3.157	1.135	0.086	143	1.172
BKK34360	*epsB*	-3.157	1.113	0.096	410	1.149
BKK01010	*nusG*	-3.16	1.150	0.133	98	1.187
BKK08720	*ygaF*	-3.163	1.122	0.108	460	1.159
BKK24480	*yqhR*	-3.164	1.150	0.100	225	1.187
BKK30850	*ytdA*	-3.165	1.183	0.059	48	1.222
BKK27750	*bofC*	-3.168	1.123	0.096	254	1.160
BKK23500	*drm*	-3.182	1.117	0.098	469	1.153
BKK21140	*yonC*	-3.183	1.118	0.103	283	1.155
BKK40573	*yyzI*	-3.185	1.129	0.123	86	1.166
BKK32310	*yutD*	-3.19	1.124	0.097	399	1.161
BKK25020	*sodA*	-3.192	1.126	0.100	159	1.164
BKK19970	*yosX*	-3.196	1.093	0.142	297	1.129
BKK40910	*rpsF*	-3.199	1.147	0.108	212	1.184
BKK14540	*ykzG*	-3.2	0.944	0.093	45	0.975
BKK22420	*panC*	-3.206	1.121	0.112	139	1.158
BKK25280	*recO*	-3.206	1.126	0.110	82	1.164
BKK06930	*yesK*	-3.208	1.163	0.082	103	1.201
BKK09610	*yhdV*	-3.211	1.139	0.098	139	1.177
BKK12580	*xtmB*	-3.213	1.151	0.158	645	1.189
BKK21350	*yomI*	-3.215	1.118	0.106	363	1.155

Sup. Table 4: Cell width of mutants of the BKK collection (continued)

BKK name [1]	gene	screening delta[2] (%)	average width (µm)	+/-	nb	AWP
BKK09790	*yheB*	-3.22	1.139	0.102	160	1.177
BKK26100	*yqbI*	-3.22	1.139	0.092	273	1.177
BKK31790	*yueG*	-3.222	1.124	0.102	275	1.161
BKK29880	*malS*	-3.223	1.077	0.100	138	1.113
BKK27770	*yrbE*	-3.227	1.122	0.092	155	1.160
BKK14040	*ykuD*	-3.239	1.129	0.144	63	1.166
BKK37660	*pta*	-3.241	1.082	0.103	43	1.119
BKK00300	*yaaR*	-3.253	1.110	0.098	509	1.147
BKK06559	*yezF*	-3.254	1.140	0.111	323	1.179
BKK07010	*yesS*	-3.259	1.140	0.121	322	1.179
BKK14569	*ykzV*	-3.26	1.141	0.108	517	1.179
BKK17340	*ymaH*	-3.269	1.125	0.100	83	1.164
BKK17780	*yndG*	-3.27	1.125	0.105	111	1.164
BKK30610	*ytlC*	-3.287	1.182	0.097	538	1.222
BKK14870	*ctaA*	-3.294	1.140	0.100	700	1.179
BKK05970	*rex*	-3.303	1.119	0.082	66	1.158
BKK10660	*yisB*	-3.316	1.144	0.111	81	1.183
BKK32040	*yuiF*	-3.321	1.110	0.106	107	1.148
BKK11320	*yjzB*	-3.327	1.144	0.115	105	1.183
BKK06650	*sapB*	-3.332	1.140	0.101	446	1.179
BKK26970	*adhB*	-3.333	1.113	0.113	266	1.151
BKK33560	*yvaD*	-3.335	1.127	0.112	111	1.166
BKK32580	*frlM*	-3.337	1.110	0.135	34	1.148
BKK09590	*yhdT*	-3.344	1.138	0.080	117	1.177
BKK05020	*phrI*	-3.351	1.109	0.099	471	1.147
BKK34300	*epsH*	-3.353	1.111	0.099	194	1.149
BKK06870	*yesE*	-3.354	1.139	0.095	217	1.179
BKK21470	*sunT*	-3.362	1.116	0.106	467	1.155
BKK10200	*yhfE*	-3.37	1.119	0.098	274	1.158
BKK20190	*yosA*	-3.372	1.091	0.129	93	1.129
BKK33280	*yvrP*	-3.372	1.127	0.100	87	1.166
BKK13710	*ykvI*	-3.373	1.127	0.095	412	1.166
BKK18979	*yoyA*	-3.38	1.123	0.105	426	1.162
BKK22980	*ypbG*	-3.381	1.114	0.101	575	1.153
BKK39400	*pdp*	-3.384	1.125	0.092	89	1.165
BKK09150	*yhcN*	-3.386	1.137	0.103	110	1.177
BKK10930	*yitB*	-3.389	1.143	0.117	93	1.183
BKK25790	*arsB*	-3.389	0.942	0.067	55	0.975
BKK33330	*lysP*	-3.395	1.096	0.103	76	1.134
BKK17267	*ymzE*	-3.396	1.124	0.105	209	1.164
BKK26500	*yrkI*	-3.397	1.114	0.105	511	1.153
BKK03250	*ycgR*	-3.398	1.092	0.106	46	1.130
BKK21700	*ypoP*	-3.398	1.114	0.113	383	1.153
BKK30900	*cotS*	-3.401	1.117	0.109	124	1.156
BKK28890	*yscB*	-3.405	1.180	0.075	73	1.222
BKK03600	*tcyB*	-3.418	1.160	0.074	478	1.201
BKK11730	*cotO*	-3.419	1.144	0.087	86	1.184
BKK11460	*oppD*	-3.422	1.167	0.138	139	1.208
BKK09450	*yhdF*	-3.424	1.137	0.090	185	1.177
BKK04250	*lrpC*	-3.436	1.125	0.106	439	1.165
BKK11140	*yitU*	-3.455	1.142	0.113	73	1.183
BKK23780	*yqjQ*	-3.467	1.146	0.097	190	1.187
BKK05050	*lrpA*	-3.473	1.127	0.144	150	1.167
BKK36960	*ywlB*	-3.477	1.076	0.112	120	1.114
BKK02590	*ycbP*	-3.478	1.091	0.094	50	1.130
BKK07650	*yflK*	-3.479	0.941	0.063	173	0.975
BKK11960	*yjcR*	-3.489	1.148	0.130	929	1.189
BKK38470	*ywaD*	-3.499	1.080	0.109	176	1.119
BKK32610	*frlB*	-3.5	1.108	0.106	42	1.148
BKK41010	*trmF*	-3.505	1.143	0.084	169	1.184
BKK28350	*ysnB*	-3.509	1.119	0.091	379	1.160
BKK19760	*cgeD*	-3.515	1.090	0.119	447	1.129
BKK25550	*rpsT*	-3.519	1.123	0.107	179	1.164
BKK26030	*yqbO*	-3.521	1.135	0.098	271	1.177
BKK20320	*yorN*	-3.526	1.089	0.138	92	1.129
BKK04670	*rsbRA*	-3.527	1.124	0.113	335	1.165
BKK39800	*yxcD*	-3.532	1.124	0.094	35	1.165
BKK06660	*opuE*	-3.537	1.159	0.081	125	1.201
BKK03110	*ycgH*	-3.54	1.159	0.079	59	1.201
BKK21710	*dinF*	-3.54	1.113	0.091	278	1.153
BKK39430	*deoR*	-3.547	1.123	0.097	111	1.165
BKK17678	*ynzJ*	-3.551	1.122	0.114	97	1.164
BKK35010	*nagA*	-3.553	1.095	0.121	463	1.135
BKK03780	*phrC*	-3.557	1.107	0.095	545	1.147
BKK17970	*cotM*	-3.572	1.163	0.126	121	1.206
BKK26240	*yqaO*	-3.575	1.135	0.121	168	1.177
BKK30400	*bceR*	-3.578	1.115	0.092	136	1.156
BKK00800	*yazB*	-3.586	1.061	0.121	391	1.100
BKK08370	*yfiR*	-3.589	0.940	0.072	60	0.975
BKK36080	*ywrF*	-3.595	1.074	0.089	41	1.114
BKK10410	*yhzC*	-3.599	1.141	0.102	84	1.183
BKK09830	*yhaX*	-3.6	1.135	0.110	118	1.177
BKK07150	*yetG*	-3.602	1.136	0.119	289	1.179
BKK27180	*yrhH*	-3.605	1.112	0.102	274	1.153
BKK00710	*hslO*	-3.607	1.113	0.099	356	1.155
BKK27020	*yraA*	-3.608	1.134	0.098	379	1.177
BKK14480	*abh*	-3.61	1.121	0.102	535	1.163
BKK17260	*aprX*	-3.611	1.122	0.098	158	1.164
BKK07540	*yfmA*	-3.613	1.118	0.096	627	1.160
BKK28840	*ysdA*	-3.614	1.073	0.116	106	1.113
BKK39950	*yxaJ*	-3.614	1.123	0.101	114	1.165
BKK20080	*yosL*	-3.619	0.940	0.080	38	0.975
BKK29249	*ytzJ*	-3.62	1.073	0.117	108	1.113
BKK05280	*ydeO*	-3.645	1.125	0.088	99	1.167
BKK23880	*yqzJ*	-3.651	1.144	0.098	324	1.187
BKK02930	*yceG*	-3.654	1.106	0.100	616	1.147
BKK19749	*yoyG*	-3.667	1.088	0.152	196	1.129
BKK36250	*ptkA*	-3.668	1.107	0.105	358	1.149
BKK19720	*yodR*	-3.67	1.088	0.093	404	1.129
BKK32150	*paiA*	-3.672	1.106	0.100	272	1.148
BKK13850	*zosA*	-3.676	1.162	0.141	155	1.206
BKK37370	*albA*	-3.678	1.078	0.095	136	1.119
BKK33760	*sdpB*	-3.694	1.123	0.098	97	1.166
BKK37930	*ywdK*	-3.709	1.107	0.090	210	1.149
BKK14460	*ykpC*	-3.721	1.135	0.136	765	1.179
BKK23410	*spoVAD*	-3.724	1.143	0.096	457	1.187
BKK34310	*epsG*	-3.724	1.093	0.119	56	1.135
BKK21580	*yokI*	-3.73	1.112	0.091	566	1.155
BKK24320	*nusB*	-3.732	0.939	0.070	33	0.975
BKK37870	*spsE*	-3.735	1.107	0.098	536	1.149
BKK36590	*clsA*	-3.737	1.073	0.122	124	1.114
BKK32270	*yutH*	-3.738	1.105	0.106	90	1.148
BKK23590	*ansR*	-3.751	1.143	0.095	227	1.187
BKK12660	*xkdM*	-3.759	1.144	0.132	612	1.189
BKK38280	*efeU*	-3.772	1.077	0.104	58	1.119
BKK04750	*ydcF*	-3.778	1.121	0.136	564	1.165
BKK09700	*yheJ*	-3.779	1.133	0.104	105	1.177
BKK32880	*yusP*	-3.779	1.176	0.100	100	1.222
BKK09940	*yhaL*	-3.79	1.132	0.097	159	1.177
BKK00860	*clpC*	-3.799	1.058	0.117	107	1.100
BKK18700	*yoaQ*	-3.803	1.124	0.106	271	1.168
BKK26960	*yraF*	-3.807	1.132	0.108	202	1.177
BKK11250	*argF*	-3.819	1.138	0.104	86	1.183
BKK40450	*yycD*	-3.82	1.122	0.096	163	1.166
BKK02810	*cwlK*	-3.825	0.938	0.082	36	0.975
BKK36520	*glnK*	-3.83	1.072	0.132	124	1.114
BKK10650	*sbcC*	-3.832	1.138	0.096	97	1.183
BKK15310	*spoIIGA*	-3.832	1.160	0.124	93	1.206
BKK21480	*sunA*	-3.836	1.111	0.108	268	1.155
BKK18899	*yozX*	-3.839	1.123	0.110	308	1.168
BKK27170	*bscR*	-3.839	1.132	0.102	83	1.177
BKK15550	*pyrF*	-3.84	1.096	0.098	60	1.139
BKK14660	*ykzI*	-3.854	1.134	0.129	292	1.179
BKK00090	*guaB*	-3.863	1.133	0.135	122	1.179
BKK35180	*csbA*	-3.866	1.091	0.106	540	1.135
BKK37540	*ywhB*	-3.866	1.075	0.114	68	1.119
BKK21600	*yokG*	-3.871	1.110	0.108	369	1.155
BKK28830	*ysdB*	-3.876	1.070	0.094	74	1.113
BKK19840	*yotL*	-3.883	1.085	0.134	281	1.129
BKK02030	*ybdM*	-3.888	1.086	0.090	31	1.130
BKK03420	*nin*	-3.897	1.086	0.104	116	1.130
BKK23300	*ypuD*	-3.899	1.141	0.100	84	1.187
BKK15740	*rsmB*	-3.92	1.090	0.099	114	1.134
BKK14250	*yknT*	-3.924	1.133	0.114	285	1.179
BKK11055	*yitO*	-3.936	1.137	0.110	90	1.183
BKK35550	*tuaG*	-3.947	1.104	0.093	553	1.149
BKK13440	*ykoY*	-3.951	1.117	0.107	228	1.163
BKK37630	*ywfM*	-3.953	1.104	0.104	290	1.149
BKK21830	*ypjQ*	-3.954	1.112	0.097	125	1.158
BKK26200	*yqaS*	-3.957	1.130	0.107	234	1.177
BKK13320	*ykzB*	-3.975	1.120	0.092	92	1.166
BKK13789	*ykzQ*	-3.975	1.159	0.133	121	1.206
BKK24990	*pstS*	-3.978	1.158	0.156	108	1.206
BKK24360	*spoIIIAH*	-3.997	1.158	0.136	37	1.206
BKK16340	*fliZ*	-4.004	1.115	0.095	163	1.162
BKK14071	*fadG*	-4.011	1.116	0.099	391	1.163
BKK40760	*yyaP*	-4.018	1.137	0.098	178	1.184
BKK04790	*ydcK*	-4.026	1.118	0.146	53	1.165
BKK03010	*amhX*	-4.043	1.084	0.074	32	1.130
BKK17559	*ynzI*	-4.045	1.115	0.093	526	1.162
BKK35620	*lytC*	-4.049	1.114	0.103	339	1.161
BKK13540	*ogt*	-4.073	1.119	0.105	204	1.166
BKK04760	*ydcG*	-4.077	1.117	0.152	168	1.165
BKK04950	*yddF*	-4.078	1.120	0.120	192	1.167
BKK25170	*yqfO*	-4.091	1.116	0.097	313	1.164
BKK16610	*ylxR*	-4.097	1.120	0.116	327	1.168
BKK34490	*yvdS*	-4.098	1.089	0.113	297	1.135
BKK37120	*fbaA*	-4.113	1.073	0.088	41	1.119
BKK10400	*yhxC*	-4.116	1.135	0.095	95	1.183
BKK26850	*yrpG*	-4.125	1.128	0.100	173	1.177
BKK15360	*ylmC*	-4.127	1.130	0.156	58	1.179
BKK36360	*mscL*	-4.129	1.113	0.099	373	1.161
BKK04430	*ydbD*	-4.132	1.112	0.098	537	1.160
BKK09030	*yhcC*	-4.143	1.128	0.101	105	1.177
BKK20560	*yoqO*	-4.143	1.083	0.119	63	1.129
BKK00360	*yabC*	-4.151	1.130	0.121	206	1.179
BKK27360	*yrrM*	-4.152	1.111	0.095	162	1.160
BKK11310	*comZ*	-4.157	1.134	0.126	120	1.183
BKK20240	*yorV*	-4.158	1.082	0.152	32	1.129
BKK14020	*ykyB*	-4.169	1.118	0.115	263	1.166
BKK36970	*spoIIR*	-4.174	1.101	0.097	368	1.149
BKK28420	*ysmA*	-4.186	0.934	0.072	45	0.975
BKK10220	*gltT*	-4.205	1.127	0.108	206	1.177
BKK19260	*yocM*	-4.211	1.122	0.115	39	1.172
BKK10290	*yhfN*	-4.215	1.133	0.099	135	1.183
BKK24390	*spoIIIAE*	-4.219	1.156	0.152	36	1.206
BKK04330	*mutT*	-4.22	1.116	0.137	405	1.165
BKK14400	*fruA*	-4.227	1.129	0.124	701	1.179
BKK19910	*yotE*	-4.23	1.082	0.126	111	1.129
BKK15010	*ylbH*	-4.237	1.129	0.093	295	1.179
BKK37050	*maeA*	-4.238	1.067	0.145	60	1.114
BKK25410	*rpsU*	-4.24	1.114	0.102	277	1.164
BKK37600	*ywfO*	-4.243	1.071	0.116	355	1.119
BKK34810	*yvcD*	-4.246	1.087	0.120	38	1.135
BKK05830	*gmuC*	-4.253	1.118	0.098	150	1.167
BKK35319	*yvzG*	-4.258	1.117	0.104	141	1.166
BKK16710	*mlpA*	-4.265	1.114	0.105	137	1.164
BKK09300	*glpD*	-4.267	1.127	0.096	306	1.177
BKK25710	*cwlH*	-4.267	1.114	0.108	127	1.164
BKK18210	*yngE*	-4.273	1.122	0.083	93	1.172
BKK01560	*kbaA*	-4.282	1.126	0.100	278	1.177
BKK09290	*glpK*	-4.282	1.127	0.095	239	1.177
BKK20430	*yorC*	-4.284	1.081	0.134	168	1.129
BKK16560	*rseP*	-4.286	1.112	0.095	344	1.161
BKK20550	*yoqP*	-4.289	1.081	0.113	131	1.129
BKK23920	*yqjD*	-4.299	1.136	0.094	539	1.187
BKK18819	*yozU*	-4.31	1.121	0.121	242	1.172
BKK31720	*degQ*	-4.329	1.111	0.098	262	1.161
BKK16320	*fliY*	-4.343	1.117	0.102	233	1.168
BKK05960	*moaC*	-4.346	1.116	0.143	429	1.167
BKK22370	*aspB*	-4.347	1.052	0.113	246	1.100
BKK09410	*phoA*	-4.354	1.126	0.096	124	1.177
BKK38750	*cydB*	-4.356	1.115	0.093	133	1.166
BKK17560	*ynaI*	-4.358	1.113	0.098	106	1.164
BKK20660	*yoqE*	-4.36	1.111	0.108	325	1.162
BKK34960	*yvoF*	-4.365	1.086	0.132	229	1.135
BKK05099	*ydzM*	-4.366	1.116	0.108	265	1.167
BKK38700	*sigY*	-4.369	1.114	0.126	108	1.165
BKK12430	*rapA*	-4.37	1.137	0.122	337	1.189
BKK37880	*spsD*	-4.37	1.070	0.088	48	1.119
BKK16400	*flhF*	-4.375	1.117	0.097	396	1.168
BKK24130	*prpD*	-4.381	1.135	0.104	362	1.187
BKK23690	*yqjY*	-4.394	1.135	0.091	466	1.187
BKK14050	*ykuE*	-4.402	1.115	0.087	38	1.166
BKK11920	*yjcN*	-4.404	1.137	0.112	461	1.189
BKK11990	*yjdB*	-4.405	0.932	0.069	59	0.975
BKK22240	*ypqA*	-4.438	1.100	0.108	384	1.151
BKK14800	*ylaJ*	-4.439	1.153	0.128	77	1.206
BKK30290	*amyC*	-4.441	1.168	0.100	39	1.222
BKK29030	*ytcD*	-4.444	1.089	0.087	120	1.139
BKK32430	*pucJ*	-4.444	1.097	0.125	117	1.148
BKK04300	*ydaM*	-4.447	1.113	0.116	664	1.165
BKK18370	*yoeA*	-4.451	1.153	0.128	99	1.206
BKK22470	*ypjG*	-4.455	1.051	0.124	189	1.100
BKK36710	*fdhD*	-4.455	1.065	0.100	67	1.114
BKK36260	*ywqC*	-4.467	1.098	0.099	537	1.149
BKK05010	*rapI*	-4.478	1.096	0.098	520	1.147
BKK25310	*dgkA*	-4.491	1.111	0.099	473	1.164
BKK11430	*oppA*	-4.499	1.131	0.091	184	1.184
BKK36030	*ywrK*	-4.499	1.109	0.094	476	1.161
BKK11030	*yitL*	-4.506	1.130	0.105	101	1.183
BKK01910	*skfA*	-4.507	1.063	0.112	125	1.113
BKK21500	*uvrX*	-4.507	1.099	0.087	250	1.151
BKK36190	*ywqJ*	-4.507	1.064	0.100	195	1.114
BKK34540	*clpP*	-4.517	0.931	0.054	36	0.975
BKK26470	*yrkL*	-4.523	1.123	0.100	311	1.177
BKK38210	*ywcD*	-4.527	1.113	0.104	138	1.166
BKK36470	*pucI*	-4.534	1.064	0.108	62	1.114
BKK33790	*sdpR*	-4.55	1.113	0.091	226	1.166
BKK05860	*gmuE*	-4.552	1.114	0.088	305	1.167
BKK10850	*yisS*	-4.558	1.129	0.109	73	1.183
BKK25970	*yqcA*	-4.558	0.931	0.068	64	0.975
BKK34700	*yvcR*	-4.564	1.097	0.098	406	1.149
BKK09270	*glpP*	-4.568	1.123	0.107	161	1.177
BKK01030	*rplA*	-4.57	1.110	0.095	223	1.164
BKK23450	*sigF*	-4.571	1.133	0.090	301	1.187
BKK27630	*yrvD*	-4.583	1.106	0.096	377	1.160
BKK06950	*yesM*	-4.585	1.146	0.096	151	1.201
BKK29470	*ackA*	-4.595	1.062	0.133	35	1.113
BKK02100	*cypC*	-4.597	1.078	0.117	37	1.130
BKK03370	*yckA*	-4.601	1.078	0.094	106	1.130
BKK35380	*fliW*	-4.612	1.083	0.113	531	1.135
BKK23770	*dsdA*	-4.62	1.132	0.095	216	1.187
BKK19310	*dhaS*	-4.642	1.117	0.092	136	1.172
BKK40670	*yybE*	-4.648	1.129	0.094	248	1.184
BKK16150	*clpQ*	-4.649	1.106	0.101	470	1.160
BKK05220	*ydeJ*	-4.679	1.113	0.145	91	1.167
BKK38190	*galT*	-4.685	1.066	0.107	79	1.119
BKK36330	*ywpF*	-4.692	1.107	0.101	437	1.161
BKK19130	*yocA*	-4.707	1.116	0.105	99	1.172
BKK23480	*dacF*	-4.71	1.150	0.165	58	1.206
BKK29120	*mdh*	-4.727	1.060	0.088	105	1.113
BKK12410	*yjoA*	-4.729	1.133	0.143	638	1.189
BKK32260	*hom*	-4.73	1.086	0.079	69	1.139
BKK11150	*yitV*	-4.739	1.127	0.105	113	1.183
BKK34670	*yvdA*	-4.739	1.081	0.111	207	1.135
BKK04530	*ydbN*	-4.74	1.110	0.132	519	1.165
BKK34640	*yvdD*	-4.745	1.081	0.141	94	1.135
BKK24120	*prpB*	-4.757	1.131	0.098	252	1.187
BKK34890	*hisH*	-4.766	1.081	0.123	256	1.135
BKK00650	*yabS*	-4.774	1.075	0.096	189	1.129
BKK09180	*yhcQ*	-4.784	1.121	0.096	245	1.177
BKK41000	*rsmG*	-4.785	1.128	0.101	132	1.184
BKK37820	*spsK*	-4.787	1.094	0.095	637	1.149
BKK37950	*ywdI*	-4.791	1.065	0.104	135	1.119
BKK12690	*xkdP*	-4.799	1.112	0.109	284	1.168
BKK14620	*slp*	-4.824	1.148	0.099	30	1.206
BKK23490	*pupG*	-4.83	1.130	0.095	606	1.187
BKK00200	*yaaK*	-4.842	1.120	0.098	112	1.177
BKK37890	*spsC*	-4.844	1.094	0.109	420	1.149
BKK07240	*yetN*	-4.848	1.122	0.106	358	1.179
BKK12749	*xkzA*	-4.85	1.110	0.111	304	1.166
BKK10050	*ecsB*	-4.852	1.120	0.097	133	1.177
BKK35610	*tuaA*	-4.853	1.094	0.109	551	1.149
BKK08810	*senS*	-4.855	1.103	0.093	320	1.159
BKK09920	*yhaN*	-4.864	1.120	0.104	87	1.177
BKK13910	*ptsI*	-4.878	1.109	0.141	37	1.166
BKK37100	*murAB*	-4.883	1.093	0.107	138	1.149
BKK10820	*yisQ*	-4.889	1.082	0.099	56	1.138
BKK28000	*minC*	-4.891	1.103	0.092	148	1.160
BKK32850	*fadM*	-4.908	1.079	0.092	71	1.134
BKK03990	*mtlD*	-4.91	1.108	0.108	181	1.165
BKK36668	*ywzE*	-4.915	1.060	0.103	164	1.114
BKK12160	*yjgC*	-4.947	1.130	0.138	416	1.189
BKK33540	*yvaB*	-4.95	0.927	0.077	67	0.975
BKK20140	*yosF*	-4.958	1.073	0.118	34	1.129
BKK19660	*yozD*	-4.959	1.073	0.138	455	1.129
BKK32730	*metN*	-4.964	1.161	0.114	157	1.222
BKK11839	*yjzE*	-4.985	1.130	0.103	859	1.189
BKK04240	*ydzA*	-4.989	1.107	0.105	284	1.165
BKK09800	*yheA*	-4.993	1.118	0.103	137	1.177
BKK18910	*rapK*	-5.013	1.113	0.103	123	1.172
BKK19210	*yocH*	-5.019	1.146	0.139	59	1.206
BKK22200	*cotD*	-5.019	1.045	0.142	141	1.100
BKK12860	*ykbA*	-5.021	1.108	0.126	173	1.166
BKK33020	*cssS*	-5.025	1.108	0.098	126	1.166

Sup. Table 4: Cell width of mutants of the BKK collection (continued)

BKK name [1]	gene	screening delta[2] (%)	average width (µm)	+/-	nb	AWP
BKK35490	*degU*	-5.028	1.103	0.094	353	1.161
BKK14220	*ykuU*	-5.047	1.120	0.137	196	1.179
BKK19770	*cgeC*	-5.049	1.072	0.116	409	1.129
BKK00640	*spoIIE*	-5.063	1.072	0.165	177	1.129
BKK32230	*yuxL*	-5.063	1.082	0.082	98	1.139
BKK05210	*ydeI*	-5.069	1.108	0.096	109	1.167
BKK28370	*rph*	-5.069	1.099	0.101	155	1.158
BKK39720	*iolE*	-5.075	1.106	0.118	77	1.165
BKK21850	*ypiP*	-5.086	1.096	0.100	509	1.155
BKK13270	*ykoI*	-5.104	1.107	0.112	287	1.166
BKK04080	*kipI*	-5.122	1.089	0.094	445	1.147
BKK07110	*lplB*	-5.123	1.118	0.096	317	1.179
BKK04360	*mntH*	-5.141	1.100	0.112	447	1.160
BKK29090	*polA*	-5.16	1.056	0.129	60	1.113
BKK37250	*narI*	-5.167	1.061	0.096	52	1.119
BKK34265	*epsK*	-5.179	1.076	0.097	424	1.135
BKK23760	*coaA*	-5.185	1.126	0.092	230	1.187
BKK05900	*thiL*	-5.19	1.107	0.121	370	1.167
BKK05980	*tatAY*	-5.201	1.088	0.096	277	1.147
BKK36210	*ywqH*	-5.201	1.056	0.105	299	1.114
BKK36290	*ywpJ*	-5.219	1.056	0.121	169	1.114
BKK38290	*thiE*	-5.222	1.060	0.102	69	1.119
BKK13350	*ykoN*	-5.223	1.105	0.103	305	1.166
BKK06100	*ydiS*	-5.237	1.106	0.112	395	1.167
BKK00740	*pabB*	-5.238	1.043	0.145	55	1.100
BKK14320	*yknU*	-5.243	1.143	0.143	136	1.206
BKK28130	*hemB*	-5.281	1.098	0.126	56	1.160
BKK37080	*rho*	-5.283	1.060	0.114	150	1.119
BKK24040	*bkdAB*	-5.286	1.124	0.096	57	1.187
BKK16970	*ymdB*	-5.293	1.102	0.099	95	1.164
BKK36780	*ywzB*	-5.303	1.055	0.123	105	1.114
BKK38690	*yxlC*	-5.32	1.138	0.098	184	1.201
BKK32130	*guaC*	-5.331	1.087	0.117	176	1.148
BKK18600	*yozQ*	-5.334	1.077	0.106	38	1.138
BKK31945	*yukJ*	-5.337	1.079	0.103	69	1.139
BKK37470	*phrF*	-5.346	1.088	0.098	284	1.149
BKK28730	*araQ*	-5.352	1.078	0.106	33	1.139
BKK00850	*mcsB*	-5.355	1.041	0.117	384	1.100
BKK03080	*ycgE*	-5.36	1.069	0.115	43	1.130
BKK13890	*ptsG*	-5.372	1.125	0.113	244	1.189
BKK36500	*ywoB*	-5.372	1.054	0.112	54	1.114
BKK22690	*aroH*	-5.383	1.041	0.110	175	1.100
BKK36750	*spoIID*	-5.389	1.054	0.112	82	1.114
BKK12120	*yjfB*	-5.402	1.125	0.150	170	1.189
BKK35850	*ywtE*	-5.418	1.054	0.109	232	1.114
BKK32750	*metQ*	-5.42	1.156	0.076	124	1.222
BKK34500	*yvdR*	-5.445	1.103	0.089	116	1.166
BKK31770	*yueI*	-5.453	1.085	0.111	190	1.148
BKK24810	*yqgV*	-5.456	1.120	0.085	254	1.184
BKK38460	*tyrZ*	-5.457	1.058	0.120	209	1.119
BKK13360	*ykoP*	-5.467	1.103	0.103	133	1.166
BKK06090	*ydiR*	-5.478	1.103	0.116	63	1.167
BKK16900	*ymfK*	-5.503	1.104	0.095	484	1.168
BKK31120	*lytG*	-5.51	1.155	0.114	35	1.222
BKK24770	*mgsR*	-5.511	1.099	0.120	148	1.164
BKK14940	*ylbA*	-5.527	1.114	0.085	267	1.179
BKK20370	*yorI*	-5.528	1.067	0.130	64	1.129
BKK11520	*mecA*	-5.533	1.118	0.103	81	1.183
BKK19950	*sspC*	-5.539	1.067	0.150	182	1.129
BKK03760	*yclK*	-5.543	1.135	0.108	308	1.201
BKK21730	*ypmS*	-5.56	1.091	0.100	321	1.155
BKK18790	*yoaZ*	-5.564	1.106	0.126	34	1.172
BKK23040	*fer*	-5.575	1.039	0.127	159	1.100
BKK12540	*xkdD*	-5.596	1.122	0.146	699	1.189
BKK36480	*ywoD*	-5.598	1.052	0.104	113	1.114
BKK35000	*hprK*	-5.65	1.071	0.123	123	1.135
BKK35870	*pgsE*	-5.661	1.051	0.132	83	1.114
BKK40510	*yybT*	-5.677	1.100	0.103	128	1.166
BKK11040	*yitM*	-5.698	1.116	0.103	82	1.183
BKK19579	*yoyD*	-5.717	1.065	0.104	462	1.129
BKK13080	*ykkB*	-5.729	1.100	0.122	341	1.166
BKK19730	*yodS*	-5.735	1.065	0.085	361	1.129
BKK13740	*queE*	-5.743	1.099	0.117	417	1.166
BKK09930	*yhaM*	-5.753	1.109	0.088	144	1.177
BKK05930	*rimI*	-5.775	1.100	0.114	469	1.167
BKK15340	*ylmA*	-5.795	1.137	0.138	72	1.206
BKK00150	*dgk*	-5.807	1.110	0.110	188	1.179
BKK18650	*pelB*	-5.827	1.103	0.123	37	1.172
BKK37860	*spsF*	-5.842	1.053	0.133	51	1.119
BKK37570	*mmr*	-5.904	1.082	0.096	313	1.149
BKK12370	*exuR*	-5.917	1.119	0.124	591	1.189
BKK36770	*ywmB*	-5.935	1.048	0.115	118	1.114
BKK36680	*ywmF*	-6.051	1.047	0.104	38	1.114
BKK09050	*yhcE*	-6.061	1.106	0.098	147	1.177
BKK15420	*divIVA*	-6.061	0.916	0.082	59	0.975
BKK14610	*pdhD*	-6.065	1.108	0.135	50	1.179
BKK37690	*bacF*	-6.105	1.050	0.091	51	1.119
BKK15580	*cysP*	-6.125	1.078	0.113	180	1.148
BKK21840	*ypjP*	-6.132	1.084	0.092	301	1.155
BKK22230	*ypqE*	-6.15	1.033	0.094	68	1.100
BKK28440	*sdhA*	-6.151	0.915	0.055	47	0.975
BKK35830	*ywtG*	-6.225	1.078	0.098	291	1.149
BKK40620	*yybJ*	-6.234	1.094	0.142	88	1.167
BKK28770	*araL*	-6.238	1.044	0.104	85	1.113
BKK22320	*ponA*	-6.259	1.081	0.128	394	1.153
BKK14629	*ykzW*	-6.274	1.105	0.123	561	1.179
BKK18709	*yozT*	-6.295	1.095	0.097	377	1.168
BKK20510	*yoqU*	-6.361	1.057	0.110	64	1.129
BKK18909	*yozZ*	-6.405	1.097	0.128	169	1.172
BKK19820	*yotN*	-6.465	1.056	0.100	822	1.129
BKK14950	*ylbB*	-6.466	1.103	0.092	122	1.179
BKK34980	*yvoD*	-6.499	1.061	0.109	241	1.135
BKK00470	*purR*	-6.679	1.086	0.103	97	1.164
BKK34900	*hisB*	-6.697	1.059	0.132	224	1.135
BKK12330	*yjmD*	-6.79	1.108	0.132	661	1.189
BKK18480	*proH*	-6.817	1.092	0.113	162	1.172
BKK38780	*yxkI*	-6.825	1.085	0.104	133	1.165
BKK19280	*yocN*	-6.839	1.091	0.131	344	1.172
BKK36300	*glcR*	-6.844	1.038	0.122	159	1.114
BKK29550	*ytcJ*	-6.86	1.037	0.127	55	1.113
BKK22510	*ypjC*	-6.867	1.025	0.117	53	1.100
BKK36900	*glyA*	-6.868	0.908	0.093	50	0.975
BKK20520	*yoqT*	-6.872	1.052	0.130	198	1.129
BKK22520	*ypjB*	-6.873	1.025	0.127	101	1.100
BKK13900	*ptsH*	-6.914	1.107	0.116	41	1.189
BKK04710	*rsbV*	-6.939	1.084	0.114	273	1.165
BKK23130	*resC*	-6.943	1.024	0.116	207	1.100
BKK16070	*ylqG*	-6.983	1.023	0.148	134	1.100
BKK13210	*thiX*	-7.014	1.085	0.142	40	1.166
BKK22820	*yphE*	-7.032	1.023	0.103	46	1.100
BKK04510	*ydbL*	-7.047	1.083	0.126	663	1.165
BKK20290	*yorQ*	-7.086	1.049	0.132	74	1.129
BKK35370	*csrA*	-7.091	1.055	0.099	132	1.135
BKK06110	*ydjA*	-7.15	1.084	0.102	369	1.167
BKK33360	*yvgJ*	-7.2	1.134	0.104	215	1.222
BKK29640	*ytsP*	-7.208	1.033	0.094	123	1.113
BKK28760	*araM*	-7.221	1.033	0.100	80	1.113
BKK36460	*ywoF*	-7.232	1.034	0.090	30	1.114
BKK20270	*yorS*	-7.257	1.047	0.136	40	1.129
BKK28120	*hemL*	-7.261	1.075	0.093	185	1.160
BKK22390	*ypmA*	-7.278	1.020	0.133	164	1.100
BKK37440	*ywhL*	-7.291	1.037	0.104	101	1.119
BKK01920	*skfB*	-7.317	1.032	0.098	110	1.113
BKK29350	*tcyM*	-7.331	1.055	0.089	48	1.138
BKK10980	*yitG*	-7.353	1.119	0.116	43	1.208
BKK05680	*ydgK*	-7.423	1.112	0.082	220	1.201
BKK20260	*yorT*	-7.443	1.045	0.130	38	1.129
BKK29440	*argH*	-7.459	1.030	0.111	30	1.113
BKK36430	*usd*	-7.477	1.031	0.092	84	1.114
BKK11770	*cotW*	-7.497	1.100	0.099	279	1.189
BKK37510	*pbpG*	-7.549	1.063	0.096	228	1.149
BKK18730	*yoaS*	-7.576	1.083	0.120	71	1.172
BKK14230	*ykuV*	-7.642	1.089	0.123	156	1.179
BKK00870	*radA*	-7.706	1.015	0.122	45	1.100
BKK14920	*ctaF*	-7.782	1.087	0.085	361	1.179
BKK37640	*ywfL*	-7.8	0.899	0.073	40	0.975
BKK40490	*yycA*	-7.876	1.073	0.118	255	1.165
BKK15600	*cysC*	-7.935	1.057	0.118	187	1.148
BKK32740	*metP*	-7.952	1.074	0.096	103	1.166
BKK35270	*cccB*	-8.013	1.044	0.129	273	1.135
BKK19290	*yozO*	-8.249	1.075	0.139	549	1.172
BKK18760	*yoaU*	-8.275	1.075	0.115	81	1.172
BKK11720	*fabI*	-8.301	1.090	0.127	90	1.189
BKK26710	*azlC*	-8.397	1.054	0.122	257	1.151
BKK05850	*gmuR*	-8.408	1.069	0.090	154	1.167
BKK22480	*mgsA*	-8.445	1.007	0.132	95	1.100
BKK36690	*rapB*	-8.446	1.020	0.110	88	1.114
BKK10140	*hemY*	-8.489	1.077	0.097	162	1.177
BKK38430	*gspA*	-8.529	1.023	0.108	128	1.119
BKK36910	*ywlG*	-8.643	1.018	0.105	199	1.114
BKK00630	*yabR*	-8.798	1.056	0.101	68	1.158
BKK28170	*hemA*	-8.839	1.057	0.124	323	1.160
**BKK29180**	*pyk*	-8.872	1.062	0.119	123	1.156
**BKK15790**	*rpe*	-8.905	1.034	0.115	35	1.135
**BKK22210**	*yprB*	-8.945	1.002	0.134	110	1.100
**BKK13900**	*ptsH*	-8.969	1.062	0.126	58	1.166
**BKK19320**	*sqhC*	-9.157	1.064	0.134	55	1.172
**BKK34620**	*mdxD*	-9.39	1.028	0.120	61	1.135
**BKK03480**	*srfAA*	-9.413	1.024	0.103	38	1.130
**BKK20300**	*yorP*	-9.42	1.023	0.145	52	1.129
**BKK01889**	*ybzH*	-9.47	0.883	0.054	42	0.975
**BKK22340**	*nth*	-9.547	0.995	0.104	85	1.100
**BKK19020**	*yobN*	-9.585	1.059	0.107	36	1.172
**BKK38660**	*yxlF*	-9.702	1.028	0.068	45	1.138
**BKK06360**	*guaA*	-9.94	0.878	0.055	50	0.975
**BKK22220**	*yprA*	-10.181	0.988	0.116	38	1.100
**BKK22410**	*panD*	-10.738	0.982	0.183	31	1.100
**BKK19180**	*des*	-10.806	1.045	0.088	87	1.172
**BKK04310**	*ydaN*	-10.896	1.038	0.123	218	1.165
**BKK08680**	*ygaC*	-10.965	1.032	0.117	185	1.159
**BKK22360**	*asnS*	-12.67	0.961	0.148	37	1.100
**BKK40390**	*walH*	-13.873	0.973	0.069	43	1.130
BKK00110	*pdxS*	n/a	n/a	n/a	n/a	n/a
BKK00690	*ftsH*	n/a	n/a	n/a	n/a	n/a
BKK01670	*ybbE*	n/a	n/a	n/a	n/a	n/a
BKK27260	*mccA*	n/a	n/a	n/a	n/a	n/a
BKK31290	*yugT*	n/a	n/a	n/a	n/a	n/a
BKK33920	*tpiA*	n/a	n/a	n/a	n/a	n/a
BKK33930	*pgk*	n/a	n/a	n/a	n/a	n/a
BKK38150	*qoxC*	n/a	n/a	n/a	n/a	n/a
BKK41030	*jag*	n/a	n/a	n/a	n/a	n/a

1: underscored and bold names indicate the top 10 % and 1% delta, respectively

2: n/a : 9 mutants have no values because they did not grow after several attempts
