# Supplementary material for: A High-Content Microscopy Screening Identifies New Genes Involved in Cell Width Control in Bacillus subtilis
Source: mSystems. 2021 Nov 30;6(6):e01017-21. doi: 10.1128/mSystems.01017-21 (PMC8631317; doi:10.1128/mSystems.01017-21)
Supplement: TABLE S5 [file msystems.01017-21-st005.pdf]

Sup. Table 5: Width of the 0.5% largest and thinnest selected strains

gene		screening step					checking step				post-backcross step					backcross strains without Mg^{2+} [5]					
name	reference	width (µm)	+/-	delta[1] (%)	nb	AWP (µm)	width (µm)	+/-	delta[2] (%)	nb	width (µm)	+/-	delta[3] (%)	nb	*P* -value[4]	width (µm)	+/-	delta (%)	nb	P value[4]	group
cwlO	BKK34800	1.42	0.14	23.36	301	1.15	1.25	0.13	28.30	265	1.09	0.09	13.07	438	***	1.10	0.08	7.68	411	****	comfirmed positively affected
rodZ	BKK16910	1.29	0.11	11.40	287	1.16	1.12	0.10	14.77	199	1.08	0.08	12.55	331	****	1.07	0.09	5.52	370	**	comfirmed positively affected
rpe	BKK15790	1.03	0.12	-8.91	35	1.14	0.86	0.07	-11.39	136	1.07	0.18	11.02	207	****	1.09	0.11	6.96	357	****	comfirmed positively affected
ftsX	BKK35250	1.33	0.18	15.59	286	1.15	1.18	0.12	20.97	167	1.06	0.08	9.95	342	****	1.10	0.08	7.66	341	****	comfirmed positively affected
yaaA	BKK00030	1.28	0.12	9.94	189	1.17	1.02	0.07	4.87	188	1.06	0.09	9.72	349	***	1.13	0.09	11.38	294	****	comfirmed positively affected
ftsE	BKK35260	1.38	0.15	19.51	109	1.16	1.21	0.11	24.20	90	1.05	0.08	9.43	431	****	1.07	0.08	5.03	335	**	comfirmed positively affected
dacA	BKK00100	1.34	0.15	15.94	107	1.16	1.03	0.10	5.94	260	1.05	0.08	9.33	409	****	1.08	0.09	6.06	370	****	comfirmed positively affected
yuaC	BKK31070	1.29	0.13	11.33	238	1.16	0.99	0.08	2.04	150	1.02	0.08	5.48	405		1.01	0.07	-0.88	281		
natA	BKK02750	1.31	0.12	9.10	127	1.20	0.96	0.06	-0.86	129	1.00	0.07	4.03	331		1.03	0.09	1.63	90		
ymfD	BKK16825	1.31	0.17	12.67	82	1.16	0.93	0.07	-4.42	53	1.00	0.07	3.92	383		1.04	0.07	1.79	102		
comFC	BKK35450	1.24	0.10	11.47	90	1.11	1.03	0.06	6.07	238	1.00	0.07	3.64	351		1.06	0.07	3.60	97		
yvyF	BKK35440	1.22	0.11	9.58	161	1.11	0.99	0.07	2.07	131	1.00	0.07	3.30	367		1.02	0.08	-0.10	324		
greA	BKK27320	1.10	0.10	12.65	40	0.98	n/a	n/a	n/a	n/a	0.99	0.06	3.20	154		0.99	0.06	-2.32	154		
mdxD	BKK34620	1.03	0.12	-9.39	61	1.14	0.95	0.07	-2.70	174	0.99	0.07	3.08	405		0.99	0.07	-2.56	295		
yprB	BKK22210	1.00	0.13	-8.95	110	1.10	0.97	0.09	-0.22	127	0.99	0.08	3.06	180		0.99	0.07	-3.06	246		
srfAA	BKK03480	1.02	0.10	-9.41	38	1.13	0.92	0.08	-4.89	123	0.99	0.07	3.06	441		1.00	0.08	-1.46	287		
yobN	BKK19020	1.06	0.11	-9.59	36	1.17	0.98	0.09	1.31	124	0.99	0.08	3.03	324		0.97	0.07	-4.43	305		
yxlF	BKK38660	1.03	0.07	-9.70	45	1.14	0.97	0.06	-0.12	127	0.99	0.07	2.83	394		1.00	0.08	-1.91	324		
ypzH	BKK22849	1.28	0.13	15.99	401	1.10	1.02	0.08	4.54	78	0.99	0.08	2.73	356		0.99	0.07	-2.57	116		
ykhA	BKK13030	1.27	0.18	9.17	109	1.17	0.99	0.06	1.31	155	0.99	0.09	2.71	355		1.04	0.10	1.93	138		
yoqC [6]	BKK20680	1.32	0.18	13.23	411	1.17	0.94	0.07	-3.46	101	n/a	n/a	n/a	n/a		n/a	n/a	n/a	n/a		
yorP [6]	BKK20300	1.02	0.15	-9.42	52	1.13	1.01	0.07	3.90	189	n/a	n/a	n/a	n/a		n/a	n/a	n/a	n/a		
des	BKK19180	1.04	0.09	-10.81	87	1.17	0.98	0.07	0.72	165	0.99	0.09	2.47	424		1.02	0.07	0.29	204		
yprA	BKK22220	0.99	0.12	-10.18	38	1.10	0.99	0.08	2.14	107	0.99	0.07	2.46	276		0.99	0.07	-3.10	183		
xkdX	BKK12770	1.29	0.12	10.49	135	1.17	0.96	0.07	-0.86	224	0.98	0.07	2.04	283		1.02	0.08	-0.30	155		
yqiW	BKK23990	1.26	0.14	9.50	142	1.15	0.97	0.07	-0.31	237	0.98	0.07	1.61	324		0.97	0.08	-4.31	131		
nth	BKK22340	1.00	0.10	-9.55	85	1.10	1.00	0.08	2.44	160	0.98	0.07	1.33	267		0.99	0.08	-2.52	277		
ygaC	BKK08680	1.03	0.12	-10.97	185	1.16	0.94	0.07	-3.16	121	0.98	0.07	1.24	366		1.01	0.08	-0.55	236		
xkdW	BKK12760	1.42	0.12	21.84	150	1.17	0.99	0.08	2.14	79	0.97	0.07	1.14	316		0.98	0.06	-4.25	64		
sqhC	BKK19320	1.06	0.13	-9.16	55	1.17	0.97	0.07	-0.06	163	0.97	0.08	1.07	322		0.98	0.07	-3.88	270		
ydaN	BKK04310	1.04	0.12	-10.90	218	1.16	0.97	0.07	-0.04	119	0.97	0.06	0.74	376		1.01	0.08	-0.87	173		
kbl	BKK17000	1.28	0.16	9.83	152	1.16	0.96	0.07	-1.70	161	0.97	0.07	0.62	333		0.99	0.06	-2.71	75		
asnS	BKK22360	0.96	0.15	-12.67	37	1.10	0.97	0.07	-0.24	103	0.97	0.07	0.17	322		0.98	0.08	-3.34	100		
minJ	BKK35220	1.24	0.13	9.38	104	1.14	0.98	0.08	0.49	211	0.95	0.09	-1.02	677		0.97	0.11	-4.60	225		
walH	BKK40390	0.97	0.07	-13.87	43	1.13	0.89	0.06	-8.04	126	0.92	0.07	-4.54	366		0.92	0.07	-9.55	361		
pyk	BKK29180	1.06	0.12	-8.87	123	1.16	0.82	0.04	-15.67	105	0.88	0.06	-8.32	314	***	0.94	0.08	-7.47	289	****	comfirmed negatively
ybzH	BKK01889	0.88	0.05	-9.47	42	0.98	n/a	n/a	n/a	n/a	0.87	0.06	-9.30	369	****	0.86	0.06	-15.19	115	****	comfirmed negatively
panD	BKK22410	0.98	0.18	-10.74	31	1.10	0.82	0.05	-15.65	135	0.85	0.06	-11.39	301	****	0.87	0.06	-15.04	256	****	comfirmed negatively
guaA	BKK06360	0.88	0.05	-9.94	50	0.98	n/a	n/a	n/a	n/a	0.84	0.05	-12.73	351	****	0.81	0.05	-20.58	182	****	comfirmed negatively
ptsH	BKK13900	1.06	0.13	-8.97	58	1.17	0.83	0.06	-14.44	163	0.84	0.07	-13.30	392	****	0.91	0.06	-10.27	390	****	comfirmed negatively

1: δ relative to AWP
2: δ relative to wild type width
3: δ relative to wild type strain, average of 3 independant replicates
4: *P* -value (summary) of nested t-tests, comparing the widths of mutants with that of the wild type cells (**** = P<0.0001; *** = 0.0001<P<0.001; ** = 0.001<P<0.01; * = 0.01<P<0.05; ns = P>0.05)
5: width values for confirmed affected mutants are the average of 3 independent replicates
6: PCR checking revealed that the BKK strains are wt for the tested loci. Deletions were therefore not backcrossed into the 168 strain
