# Supplementary material for: A High-Content Microscopy Screening Identifies New Genes Involved in Cell Width Control in Bacillus subtilis
Source: mSystems. 2021 Nov 30;6(6):e01017-21. doi: 10.1128/mSystems.01017-21 (PMC8631317; doi:10.1128/mSystems.01017-21)
Supplement: TABLE S6 [file msystems.01017-21-st006.pdf]

Sup. Table 6. *B. subtilis* strains used in this study

Strain (*B. subtilis*)	Relevant genotype	Source or reference[1]
168	(wt)	Laboratory stock
PY79	(wt)	Laboratory stock
RCL413	Ω*neo3427* - Δ*mreB*	Billaudeau, 2019
PS2062	Δ*ponA* ::*spc*	Popham, 1995
CcBs351	Δ*rodZ* ::*cat*	Gibson assembly → 168
CcBs628	Δ*rodZ* ::*cat*	CcBs351→ PY79
BKK00030	Δ*yaaA* ::*km*	Koo, 2017
BKK00100	Δ*dacA* ::*km*	Koo, 2017
BKK01889	Δ*ybzH* ::*km*	Koo, 2017
BKK02750	Δ*natA* ::*km*	Koo, 2017
BKK03480	Δ*srfAA* ::*km*	Koo, 2017
BKK04310	Δ*ydaN* ::*km*	Koo, 2017
BKK06360	Δ*guaA* ::*km*	Koo, 2017
BKK08680	Δ*ygaC* ::*km*	Koo, 2017
BKK12760	Δ*xkdW* ::*km*	Koo, 2017
BKK12770	Δ*xkdX* ::*km*	Koo, 2017
BKK13030	Δ*ykhA* ::*km*	Koo, 2017
BKK13900	Δ*ptsH* ::*km*	Koo, 2017
BKK15790	Δ*rpe* ::*km*	Koo, 2017
BKK16825	Δ*ymfD* ::*km*	Koo, 2017
BKK16910	Δ*rodZ* ::*km*	Koo, 2017
BKK17000	Δ*kbl* ::*km*	Koo, 2017
BKK19020	Δ*yobN* ::*km*	Koo, 2017
BKK19180	Δ*des* ::*km*	Koo, 2017
BKK19320	Δ*sqhC* ::*km*	Koo, 2017
BKK20300	Δ*yorP* ::*km*	Koo, 2017
BKK20680	Δ*yoqC* ::*km*	Koo, 2017
BKK22210	Δ*yprB* ::*km*	Koo, 2017
BKK22220	Δ*yprA* ::*km*	Koo, 2017
BKK22340	Δ*nth* ::*km*	Koo, 2017
BKK22360	Δ*asnS* ::*km*	Koo, 2017
BKK22410	Δ*panD* ::*km*	Koo, 2017
BKK22849	Δ*ypzH* ::*km*	Koo, 2017
BKK23990	Δ*yqiw* ::*km*	Koo, 2017
BKK27320	Δ*greA* ::*km*	Koo, 2017
BKK29180	Δ*pyk* ::*km*	Koo, 2017
BKK31070	Δ*yuaC* ::*km*	Koo, 2017
BKK34620	Δ*mdxD* ::*km*	Koo, 2017
BKK34800	Δ*cwlo* ::*km*	Koo, 2017
BKK35220	Δ*minJ* ::*km*	Koo, 2017
BKK35250	Δ*ftsX* ::*km*	Koo, 2017
BKK35260	Δ*ftsE* ::*km*	Koo, 2017
BKK35440	Δ*yvyF* ::*km*	Koo, 2017
BKK35450	Δ*comFC* ::*km*	Koo, 2017
BKK38660	Δ*yxlF* ::*km*	Koo, 2017
BKK40390	Δ*walH* ::*km*	Koo, 2017
RCL0820	Δ*cwlo* ::*km*	BKK34800 DNA → 168
RCL0821	Δ*xkdW* ::*km*	BKK12760 DNA → 168
RCL0822	Δ*ftsE* ::*km*	BKK35260 DNA → 168
RCL0823	Δ*ypzH* ::*km*	BKK22849 DNA → 168
RCL0824	Δ*dacA* ::*km*	BKK00100 DNA → 168
RCL0825	Δ*ftsX* ::*km*	BKK35250 DNA → 168
RCL0826	Δ*ymfD* ::*km*	BKK16825 DNA → 168
RCL0827	Δ*comFC* ::*km*	BKK35450 DNA → 168
RCL0828	Δ*rodZ* ::*km*	BKK16910 DNA → 168
RCL0829	Δ*yuaC* ::*km*	BKK31070 DNA → 168
RCL0830	Δ*xkdX* ::*km*	BKK12770 DNA → 168
RCL0831	Δ*yaaA* ::*km*	BKK00030 DNA → 168
RCL0832	Δ*kbl* ::*km*	BKK17000 DNA → 168
RCL0833	Δ*yvyF* ::*km*	BKK35440 DNA → 168

RCL0834	Δ*minJ* ::*km*	BKK35220 DNA → 168
RCL0835	Δ*yqiw* ::*km*	BKK23990 DNA → 168
RCL0837	Δ*ykhA* ::*km*	BKK13030 DNA → 168
RCL0838	Δ*natA* ::*km*	BKK02750 DNA → 168
RCL0839	Δ*ywqA* ::*km*	BKK36280 DNA → 168
RCL0840	Δ*walH* ::*km*	BKK40390 DNA → 168
RCL0841	Δ*asnS* ::*km*	BKK22360 DNA → 168
RCL0842	Δ*ygaC* ::*km*	BKK08680 DNA → 168
RCL0843	Δ*ydaN* ::*km*	BKK04310 DNA → 168
RCL0844	Δ*des* ::*km*	BKK19180 DNA → 168
RCL0845	Δ*panD* ::*km*	BKK22410 DNA → 168
RCL0846	Δ*yprA* ::*km*	BKK22220 DNA → 168
RCL0848	Δ*yobN* ::*km*	BKK19020 DNA → 168
RCL0849	Δ*nth* ::*km*	BKK22340 DNA → 168
RCL0850	Δ*srfAA* ::*km*	BKK03480 DNA → 168
RCL0851	Δ*mdxD* ::*km*	BKK34620 DNA → 168
RCL0852	Δ*sqhC* ::*km*	BKK19320 DNA → 168
RCL0853	Δ*pyk* ::*km*	BKK29180 DNA → 168
RCL0854	Δ*ptsH* ::*km*	BKK13900 DNA → 168
RCL0855	Δ*yprB* ::*km*	BKK22210 DNA → 168
RCL0856	Δ*rpe* ::*km*	BKK15790 DNA → 168
RCL0858	Δ*greA* ::*km*	BKK27320 DNA → 168
RCL0859	Δ*ybzH* ::*km*	BKK01889 DNA → 168
RCL0860	Δ*guaA* ::*km*	BKK06360 DNA → 168

1: Arrows indicate construction by transformation with chromosomal DNA
