# Supplementary material for: A High-Content Microscopy Screening Identifies New Genes Involved in Cell Width Control in Bacillus subtilis
Source: mSystems. 2021 Nov 30;6(6):e01017-21. doi: 10.1128/mSystems.01017-21 (PMC8631317; doi:10.1128/mSystems.01017-21)
Supplement: TEXT S1 [file msystems.01017-21-s0001.pdf]

Supplementary information:

A high content microscopy screening identifies new genes involved in cell width control in *Bacillus subtilis*

Dimitri Juillot[a], Charlène Cornilleau[a], Nathalie Deboosere[b], Cyrille Billaudeau[a], Parfait Evouna-Mengue[c], Véronique Lejard[c], Priscille Brodin[b], Rut Carballido-López[a,#] and Arnaud Chastanet[a,#].

Comparison of width measurements obtained with different methods.

Because MicrobeJ was designed to determine cell width based on phase contrast images (an option not available on the HCS microscopes at our disposal) and not on fluorescent images of membrane-stained cells, we first tested the feasibility of using the plugin to our needs. Using the settings reported in Table S2, we compared the estimation of width on a population of wild type *B. subtilis* cells obtained with MicrobeJ (1) with another Fiji/ImageJ plugin, ChainTracer (2), and with a manual measurement of mid-cell widths. The measures were made on both live and fixed cells stained with the FM1-43FX membrane dye, on a Nikon N-Ti epifluorescence microscope. Additional phase contrast images were acquired as they are mandatory for the ChainTracer process (the main reason why this plugin could not be used in our screen). As seen on Fig. S1A, the distribution of cell width differs between methods, ChainTracer slightly minimizing and MicrobeJ maximizing the estimates. Although the average width measured with ChainTracer is closer to that obtained with a manual measurement than the width measured with MicrobeJ, we noticed that a large majority of cells were excluded from the automatic measurement with ChainTracer (~60 %), and that the width distribution was unexpectedly bimodal (Fig. S1A). Conversely, both MicrobeJ and the manual measures gave similar Gaussian distribution of cell widths. We also noticed a slight reduction of widths when measuring fixed cells, this time with all three methods. We concluded that, despite a larger estimation of the width, MicrobeJ is an efficient method for a relative estimation of the width, able to discriminate between average widths varying of only a few percent.

RodZ, a non-essential protein involved in cell shape control.

The *rodZ* gene was originally reported to be essential in *B. subtilis* (3). However, a more recent report indicated that its inactivation leads to robust growth and only mild shape defects (4). Furthermore, three available *B. subtilis* knockout libraries include a Δ*rodZ* mutant (5, 6). To address this discrepancy, we decided to construct by homologous recombination a new, independent *rodZ* deletion mutant (strain CcBs351).

In this new *rodZ* knockout mutant most of the open reading frame (863/914 bp) was replaced, by double cross-over recombination, with a chloramphenicol resistance cassette (*cat*). For this, the upstream and downstream flanking regions of *rodZ* were PCR amplified using chromosomal DNA of *B. subtilis* 168 as a template and oligonucleotides cc295/cc292 and cc293/cc296, respectively. The *cat* cassette was PCR amplified using cc291/cc294 as primers and pAH328 as DNA template (7). The three generated DNA fragments were combined by isothermal "Gibson" assembly (8) and transformed into wild type 168 *B. subtilis* strain, competent for natural genetic transformation. To prevent the potential appearance of suppressor mutations, transformants were selected on LB medium supplemented with 20 mM magnesium, in addition to the selection pressure. Isolated clones were subsequently checked, by sequencing the complete area that was subjected to PCR amplification. This new mutant was readily constructed. Similarly, the backcross of the Δ*rodZ* from the BKK library (BKK16910) into the wild type 168 strain was performed and gave countless positive transformants (strain RCL828), further suggesting the non-essentiality of the *rodZ* gene in *B. subtilis* in the 168 genetic background.

We then assayed for growth and shape in different conditions the three *rodZ* mutants to our disposal: the newly constructed strain (CcBs351), the original mutant from the BKK library (BKK16910) and its backcross (RCL828). In rich LB medium, the Δ*rodZ* strains displayed no difference of growth (Fig. S2A) or cell shape (Fig. S2C), but were slightly wider than their parental wild type strain (Fig. S2E). We also confirmed the presence of minicells (Fig. S3C), indicative of the perturbation of the division process as previously reported (9). When grown on the poorer MSM medium, Δ*rodZ* cells were significantly wider and frequently divided asymmetrically (Fig. S2C, E) and displayed a solid but slightly reduced growth rate compared with the wild type strain (Fig. S2A). This reduction of growth in

MSM might not be directly linked to the richness of the medium since all strains grew almost identically in the much poorer citrate/glucose-based S medium (Fig. S2A). Notably, the three *rodZ null* mutants did not grow identically. The strain BKK16910 displayed the highest reduction of maximum cell density and highest growth lag compared to the two other mutant strains. Since the backcrossed deletion of the BKK16910 (RCL828) and our independently constructed deletion mutant CcBs351 did not display this growth defect, we inferred that some unknown genetic differences in the BKK16910 strain rather than the *rodZ* deletion itself could be at play.

The mutants previously published in the literature were constructed in different genetic backgrounds, namely the 168 (4-6) and PY79 (3) wild type laboratory strains. The 168 and PY79 strains share a common origin, the ancestral 3610 "Marburg" wild isolate of *B. subtilis*, but are the product of different histories (mutagenesis and selection cycles) that drove to significant genomic differences (mutations, deletions and rearrangements) (10). We thus wondered if the impossibility to obtain a *rodZ* null mutant originally reported could have been be due to the use of the PY79 (3) instead of the 168 wild type used by us and others. We then transferred our newly constructed *rodZ* deletion into the PY79 background (strain CcBs628). The transformation caused no difficulties and the cell shape defects of the resulting CcBs628 mutant appeared minimal in rich medium, with only a slight increase of cell width (Fig. S2D, F). Again, the increased width was more pronounced when the cells were grown in poorer media (Fig. S2D, F), but overall the shapes of the Δ*rodZ* mutants were similar in both the 168 and PY79 background. The only notable difference between the 2 genetic backgrounds was a strong growth lag of the *rodZ* null in PY79, in the poor S medium (Fig. S2B).

Taken together, these results indicate that (i) *rodZ* is not essential for growth in *B. subtilis*, (ii) cells lacking *rodZ* display division defects and limited width and growth alterations, which are accentuated in poor growth medium, and (iii) that the genetic parental background influences this sensitivity to the growth medium.

List (names and sequences) of oligonucleotides:

cc291 CAAAGAAGCCAGAGAGGAAAAAGCAATGAACTTTAATAAAATTGATTTAGACAATTGG
cc292 CCAATTGTCTAAATCAATTTTATTAAAGTTCATTGCTTTTTCCTCTCTGGCTTCTTT
cc293 TAGGCCTAATGACTGGCTTTTATAATTACCAGATGACTTTTCTTCACG
cc294 GTGAAGAAAAGTCATCTGGTAATTATAAAAGCCAGTCATTAGGC
cc295 GCACTCACTAGGAAGAGAGGG
cc296 CACGTCAGAGCCTTCGATCAC

References

1. Ducret A, Quardokus EM, Brun YV. 2016. MicrobeJ, a tool for high throughput bacterial cell detection and quantitative analysis. Nature Microbiology 1.
2. Syvertsson S, Vischer NO, Gao Y, Hamoen LW. 2016. When Phase Contrast Fails: ChainTracer and NucTracer, Two ImageJ Methods for Semi-Automated Single Cell Analysis Using Membrane or DNA Staining. PLoS One 11:e0151267.
3. Muchová K, Chromiková Z, Barák I. 2013. Control of *Bacillus subtilis* cell shape by RodZ. Environmental Microbiology 15:3259-3271.
4. van Beilen J, Blohmke CJ, Folkerts H, de Boer R, Zakrzewska A, Kulik W, Vaz FM, Brul S, Ter Beek A. 2016. RodZ and PgsA Play Intertwined Roles in Membrane Homeostasis of *Bacillus subtilis* and Resistance to Weak Organic Acid Stress. Front Microbiol 7:1633.
5. Kobayashi K, Ehrlich SD, Albertini A, Amati G, Andersen KK, Arnaud M, Asai K, Ashikaga S, Aymerich S, Bessieres P, Boland F, Brignell SC, Bron S, Bunai K, Chapuis J, Christiansen LC, Danchin A, Debarbouille M, Dervyn E, Deuerling E, Devine K, Devine SK, Dreesen O, Errington J, Fillinger S, Foster SJ, Fujita Y, Galizzi A, Gardan R, Eschevins C, Fukushima T, Haga K, Harwood CR, Hecker M, Hosoya D, Hullo MF, Kakeshita H, Karamata D, Kasahara Y, Kawamura F, Koga K, Koski P, Kuwana R, Imamura D, Ishimaru M, Ishikawa S, Ishio I, Le Coq D, Masson A, Mauel C, et al. 2003. Essential *Bacillus subtilis* genes. Proc Natl Acad Sci U S A 100:4678-83.
6. Koo BM, Kritikos G, Farelli JD, Todor H, Tong K, Kimsey H, Wapinski I, Galardini M, Cabal A, Peters JM, Hachmann AB, Rudner DZ, Allen KN, Typas A, Gross CA. 2017. Construction and Analysis of Two Genome-Scale Deletion Libraries for *Bacillus subtilis*. Cell Syst 4:291-305 e7.
7. Chen Y, Cao S, Chai Y, Clardy J, Kolter R, Guo JH, Losick R. 2012. A *Bacillus subtilis* sensor kinase involved in triggering biofilm formation on the roots of tomato plants. Mol Microbiol 85:418-30.
8. Gibson DG, Young L, Chuang RY, Venter JC, Hutchison CA, 3rd, Smith HO. 2009. Enzymatic assembly of DNA molecules up to several hundred kilobases. Nat Methods 6:343-5.
9. Muchová K, Chromiková Z, Valenčíková R, Barák I. 2018. Interaction of the Morphogenic Protein RodZ with the *Bacillus subtilis* Min System. Frontiers in Microbiology 8.
10. Zeigler DR, Pragai Z, Rodriguez S, Chevreux B, Muffler A, Albert T, Bai R, Wyss M, Perkins JB. 2008. The origins of 168, W23, and other *Bacillus subtilis* legacy strains. J Bacteriol 190:6983-95.
