# Supplementary material for: A High-Content Microscopy Screening Identifies New Genes Involved in Cell Width Control in Bacillus subtilis
Source: mSystems. 2021 Nov 30;6(6):e01017-21. doi: 10.1128/mSystems.01017-21 (PMC8631317; doi:10.1128/mSystems.01017-21)
Supplement: FIG S3 [file msystems.01017-21-sf003.pdf]

Fig. Sup. 3

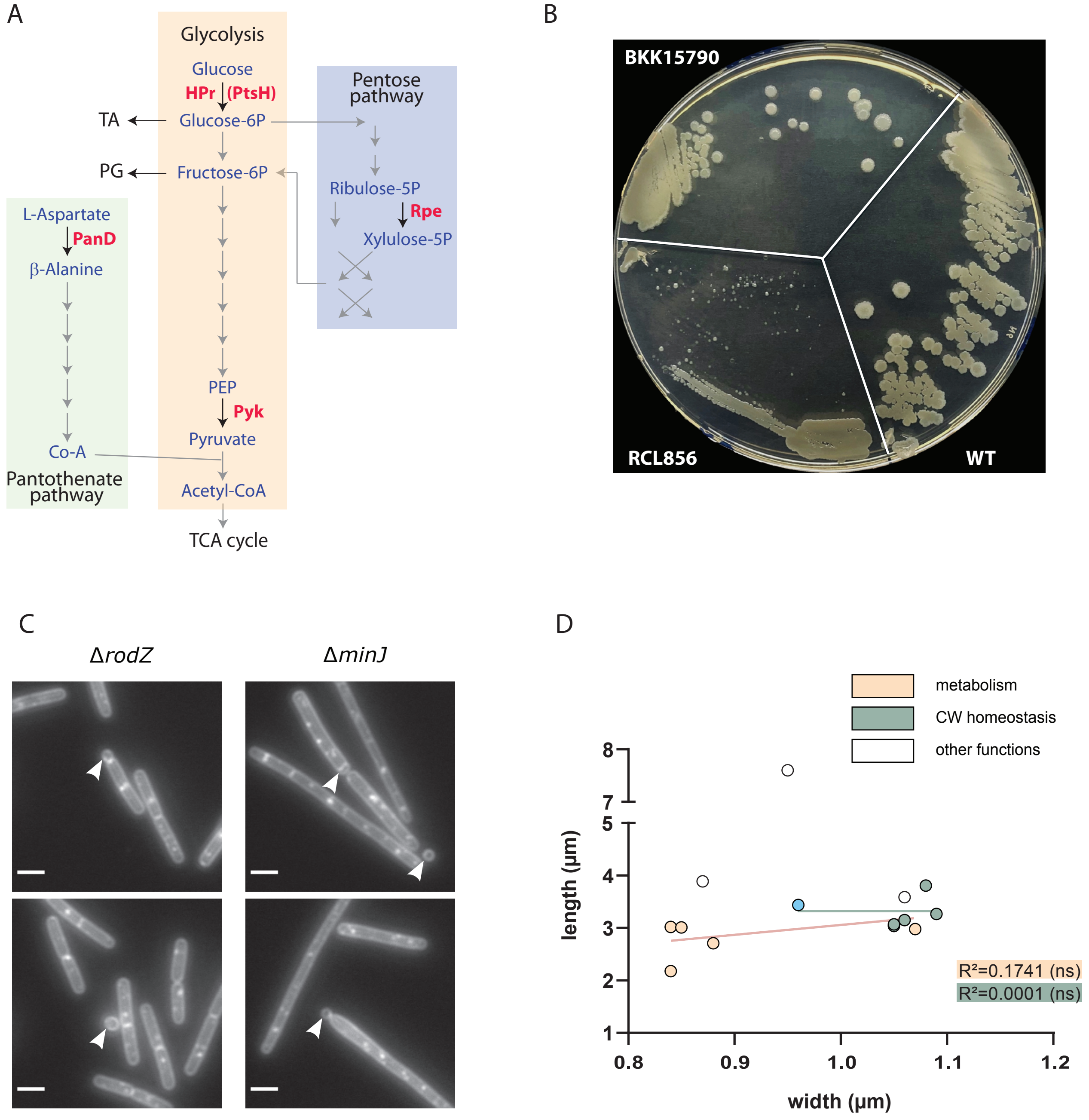

A
Glycolysis
Glucose
HPr
(PtsH)
Glucose-6P
TA
PG
Fructose-6P
Pentose pathway
Ribulose-5P
Rpe
Xylulose-5P
L-Aspartate
PanD
β-Alanine
PEP
Pyk
Pyruvate
Co-A
Acetyl-CoA
Pantothenate pathway
TCA cycle
B
BKK15790
RCL856
WT
C
ΔrodZ
ΔminJ
D
metabolism
CW homeostasis
other functions
length (μm)
width (μm)
R²=0.1741 (ns)
R²=0.0001 (ns)
